# Thirty-one-year trends in diarrheal mortality and disability-adjusted life years attributable to lack of handwashing facilities

**DOI:** 10.1186/s41182-026-00903-z

**Published:** 2026-01-17

**Authors:** Fengming Li, Zhiyong Yang, Zhifeng Lin, Xiaozhen Chen, Baiwei Yang, Shiqian Lan

**Affiliations:** 1 Gastrointestinal Endoscopy Center, Longyan First Affiliated Hospital of Fujian Medical University, Longyan, Fujian China; 2 Fujian Medical University, Fuzhou, Fujian China

**Keywords:** Lack of access to handwashing facilities, Diarrhea, GBD, DALY, SDI, Age-standardized rate, Health inequality

## Abstract

**Background:**

Diarrheal diseases remain a major global public health challenge. Hand hygiene is one of the most cost-effective interventions for preventing the transmission of diarrheal diseases. However, billions of people around the world still lack access to soap and handwashing facilities.

**Methods:**

Using the Global Burden of Disease (GBD) 2021 database, we quantified the burden of diarrhea attributable to a lack of access to handwashing facilities across 204 countries and territories from 1990 to 2021. We assessed disability-adjusted life years (DALYs), years of life lost (YLLs), and years lived with disability (YLDs), stratified by age, sex, Sociodemographic Index (SDI), and GBD region. Long-term trends were analyzed using age-standardized rates (ASRs) and estimated annual percentage changes (EAPCs). Potential non-linear associations were explored through locally weighted scatterplot smoothing (LOESS) regression.

**Results:**

Globally, the age-standardized death rate (ASDR) from diarrhea attributable to the lack of handwashing facilities declined from 14.20 (2.10–26.04) to 3.04 (0.42–5.64) per 100,000 between 1990 and 2021, with an average annual decrease of 4.89% (4.64 to 5.14). During the same period, the DALY rate decreased by 76.6%, the YLL rate by 77.9%, and the YLD rate by 32.0%. In 2021, West and East Africa remained high-burden regions, with DALY rates exceeding 600 per 100,000. South Asia recorded the largest absolute number of deaths, with nearly 90,000 fatalities. Countries with low SDI exhibited an ASDR of 17.8 (2.51–33.44) per 100,000, approximately 60 times higher than that of high-SDI countries. Mortality risk was highest among boys under five, and the absolute number of deaths increased among adults aged ≥ 70 years. YLDs were consistently higher in females than in males. Projections suggest continued declines in burden through 2035, although at a slower pace, especially in low-SDI settings.

**Conclusions:**

Although global diarrhea burdens tied to unavailable handwashing facilities have declined markedly since 1990, stark inequities persist across regions, age groups and development levels. Sustained expansion of WASH infrastructure, targeted hygiene promotion and strengthened surveillance are essential to accelerate progress toward zero preventable diarrheal deaths and universal health coverage.

**Supplementary Information:**

The online version contains supplementary material available at 10.1186/s41182-026-00903-z.

## Introduction

As the primary interface between the human body and the environment, hands often harbor a wide variety of microorganisms [1]. Insufficient handwashing practices and poor hygiene habits contribute to the cross-transmission of pathogens, resulting in illnesses such as diarrhea. Handwashing with soap is considered one of the most cost-effective interventions to mitigate the global burden of infectious diseases, particularly diarrheal diseases and acute respiratory infections [2]. A meta-analysis confirms that it reduces acute respiratory infections by 17% [3], while another indicates that hand hygiene interventions lower childhood diarrhoea risk by 33% during dry seasons [4]. By concurrently addressing these two major disease burdens with a single, low-cost behavior, handwashing with soap constitutes a fundamental and highly efficient component of primary disease prevention strategies. It is a simple, convenient, and economically efficient method to reduce the transmission of pathogens. Therefore, improving handwashing practices is crucial for decreasing hygiene-related morbidity and mortality, particularly in low-income and developing countries [5]. Recent global estimates reveal that approximately 3 billion people lack access to soap and water at home; 6 billion have limited access to handwashing facilities, lacking either soap or water; and 1.4 billion have no access to any handwashing facilities [6]. The availability of basic handwashing services varies significantly both between and within countries. In low-income settings, only one in four individuals has access to such facilities [7]. A range of contextual factors, including socio-economic conditions and hygiene education, influence access to handwashing facilities, leading to substantial disparities across regions and nations.

Despite the decline in incidence and mortality rates over recent decades, diarrhea remains a leading cause of childhood morbidity and mortality worldwide [8]. The disease is influenced by a range of environmental and physiological factors, with bacterial pathogens (e.g., *Campylobacter, Escherichia coli, Salmonella, Shigella*) and *viral infections* (e.g., norovirus, rotavirus) transmitted primarily via the fecal–oral route [9]. Although transmission occurs through multiple pathways, the fecal–oral route is most significant, with hands acting as the primary vector. While diarrheal diseases can be life-threatening, many cases are preventable through basic hygiene practices [10]. Handwashing, the core element of hand hygiene, involves washing hands with soap (either medicated or plain) and running water to remove dirt, debris, and pathogens [11, 12]. Evidence suggests that washing hands more than four times a day reduces the risk of illness [13], and promoting handwashing with soap has been shown to decrease the risk of diarrheal morbidity by 42%-47% [14]. A substantial body of research demonstrates that proper handwashing prevents the transmission of specific infectious diseases [15–18]. Increasing handwashing frequency can prevent approximately 30% of diarrhea-related illnesses [19] and around 20% of respiratory infections [20]. Community-based handwashing education programs can reduce diarrhea by 23%-40% [20–22]. For immunocompromised populations, appropriate hand hygiene can reduce the incidence of certain infectious diseases by 58% [23] and decrease the occurrence of common colds and other respiratory illnesses by 16%-21% [24]. These simple preventive actions can have enduring benefits; for example, school absenteeism due to gastrointestinal illness has been shown to decrease by 29%-57% following handwashing interventions [25]. Diarrhea remains a leading cause of death among children under five, with significant long-term consequences for growth and cognitive development [26]. The lack of access to proper handwashing facilities continues to be a major driver of diarrheal transmission, with the highest burden in low- and middle-income countries (LMICs), particularly among children under five in sub-Saharan Africa and South Asia. While the burden is greater in low-income populations, acute infectious diarrhea remains a common cause of outpatient visits and hospitalizations in high-income countries, highlighting its ongoing global health impact [27]. Risk factors for diarrheal transmission include poor living conditions, overcrowding, inadequate sanitation, and suboptimal hand hygiene practices. Within the DALY framework, diarrheal deaths occurring at young ages generate a large number of years of life lost (YLLs), which typically account for the majority of diarrheal DALYs, whereas non‑fatal episodes contribute mainly through years lived with disability (YLDs) related to acute illness and long‑term sequelae such as growth faltering.

This report provides a comprehensive assessment of the global burden of diarrheal diseases attributed to the lack of handwashing facilities. Using the Global Burden of Disease (GBD) framework, particularly the key metrics of disability-adjusted life years (DALYs), years of life lost (YLLs), and years lived with disability (YLDs).we systematically quantify the health impact associated with this risk factor.

In addition, we identify a significant data gap in current global health surveillance: authoritative sources like the GBD often subsume "inadequate hand hygiene" under the broader category of unsafe water, sanitation, and hygiene (WASH), without providing precise, standalone quantification of the specific behavioral risk associated with the lack of access to handwashing facilities. This ambiguity limits the effectiveness of targeted interventions. Therefore, we advocate for more detailed monitoring of hygiene-related behaviors and recommend policies that strengthen basic infrastructure, promote behavior change, and support further research in this area.

The Global Burden of Disease (GBD) study is a leading global public health data system that provides comprehensive, multidimensional evidence on epidemiological patterns, including prevalence, incidence, mortality, and Disability-Adjusted Life Years (DALYs). To accurately assess health loss, this report utilizes the GBD framework, as advocated by the World Health Organization (WHO) and the Institute for Health Metrics and Evaluation (IHME). By incorporating the latest disease-specific data released in 2021, we have developed a multidimensional analytical framework, which includes an age‑stratified analysis using the above five policy‑relevant age categories, with infants (< 1 year) and adults aged ≥ 75 years highlighted as key high‑risk subgroups: sex differences, geographic variation, and Sociodemographic Index (SDI) levels. This framework allows us to examine both the distribution and temporal evolution of diarrhea attributable to the lack of handwashing facilities.

DALYs, which capture the total number of healthy years of life lost due to disease onset and death, represent the gold-standard composite metric for assessing the burden of diseases, injuries, and risk factors for diarrheal transmission. By emphasizing the spatiotemporal relationship between SDI and disease burden, our aim is to inform both epidemiological research and policy decisions related to health resource allocation. Through dynamic monitoring, this approach aims to support the development of targeted, evidence-based public health interventions. Specifically, our forecasts can identify countries, subnational regions, and age-sex groups that combine high diarrheal burden with poor access to basic handwashing facilities, thereby informing screening and outreach strategies that proactively identify households and communities without functional handwashing stations, and high‑risk clusters of severe or recurrent diarrheal disease, particularly among children under five and older adults.

## Methods

### Study population and data sources

Data on the diarrheal burden attributable to the lack of access to handwashing facilities from 1990 to 2021 were obtained from the Global Health Data Exchange (GHDx) Results Tool, based on the Global Burden of Disease 2021 (GBD 2021) study. Using the latest epidemiological data and validated, standardized GBD methodologies, we conducted a comprehensive assessment of the hand-hygiene–attributable burden of diarrhea across 204 countries and territories over the period 1990–2021. The extracted indicators included the number of deaths, disability-adjusted life years (DALYs), years of life lost (YLLs), and years lived with disability (YLDs).

Measures of access to handwashing facilities were derived from the WASH exposure database used in GBD 2021. This database compiles nationally representative household surveys (e.g., Demographic and Health Surveys, Multiple Indicator Cluster Surveys, Living Standards Measurement Studies, and other country‑specific WASH surveys) that report the proportion of households with a basic handwashing facility with soap and water available on premises. Diarrheal mortality and morbidity data were drawn from vital registration systems, verbal autopsy studies, hospital discharge records, and epidemiological studies, which are synthesized in the GBD cause-of-death and non‑fatal outcome models. These primary data sources are heterogeneous in quality and coverage, particularly in low-income settings, where under-reporting, misclassification, and non-sampling error may lead to over- or under-estimation of the true burden in specific age-sex-location strata.

In accordance with GBD 2021 specifications, we obtained estimates for five‑year age groups and then collapsed them into the following policy‑relevant categories for presentation: <5 years (including infants <1 year), 5-14 years, 15-49 years, 50-74 years, and ≥75 years. Additional analyses for more granular older‑age groups (75-84 and ≥85 years) are provided in the Supplementary Tables.

In line with standard GBD definitions, years of life lost (YLLs) were calculated by multiplying the number of deaths in each age group by the corresponding standard life expectancy at that age. Years lived with disability (YLDs) were estimated by multiplying the prevalence of diarrheal sequelae by their disability weight and summing across all sequelae. Disability-adjusted life years (DALYs) represent the sum of YLLs and YLDs and reflect the total health loss due to diarrheal disease attributable to lack of handwashing facilities.

### Statistical analysis

*Sociodemographic Index (SDI)* The Sociodemographic Index (SDI) component indicators were first computed, and their geometric mean was used to summarize each location’s socioeconomic development related to health. The SDI ranges from 0 to 1, with higher values reflecting greater socioeconomic development. In the GBD 2021 study, 204 countries and territories were classified into five SDI bands—low, low-middle, middle, high-middle, and high—further grouped into 21 GBD regions based on geographic proximity, epidemiological similarities, and cause-of-death patterns.

*Stratification and Uncertainty* The analysis was stratified by age group (< 5 years, 5–14 years, 15–49 years, 50–74 years, and ≥ 75 years), SDI band (low to high), and country/territory. Over-dispersion at the survey level was accounted for in the variance. Core outcomes included the number of deaths, DALYs, YLLs, and YLDs. Uncertainty was quantified using 4,000 posterior draws, accounting for age, sex, SDI, and regional clustering. The point estimate for each stratum is the posterior median (50th percentile), and the 95% uncertainty interval (UI) is defined by the 2.5th–97.5th percentiles. All point estimates are accompanied by 95% uncertainty intervals (UIs), defined as the 2.5th-97.5th percentiles of 4,000 posterior draws from the GBD model. We report UIs for all deaths, DALYs, YLLs, and YLDs, as well as for age‑standardized rates and EAPCs.

*Age Standardization* Using the GBD standard population, we calculated age-standardized incidence rates (ASIRs), prevalence rates (ASPRs), death rates (ASDRs), and DALY rates (per 100,000 population) as: ${\mathrm{ASR}} = \frac{{\sum \nolimits_{{{\mathrm{i}} = 1}}^{{\mathrm{A}}} \alpha_{{\mathrm{i}}} {\mathrm{w}}_{{\mathrm{i}}} }}{{\sum_{{{\mathrm{i}} = 1}}^{{\mathrm{A}}} {\mathrm{w}}_{{\mathrm{i}}} }}$, where α i is the rate (e.g., incidence, DALY) in age group i, wi is the corresponding standard-population weight, and A is the total number of age groups.

Relative change and long-term trends: For 1990–2021, relative change in mortality and DALY rates attributable to lack of handwashing facilities was defined as $\frac{{{\mathrm{Number}}\,2021 - {\mathrm{Number}}\,1990}}{{{\mathrm{Number}}\,1990}} \times 100{{\% }}$. To characterize long-term trends and average annual changes over the study period, we estimated the Estimated Annual Percentage Change (EAPC) for ASIR, ASPR, ASDR, and age-standardized DALY rate using the standard GBD approach. After verifying model assumptions (see below), we fitted a log-linear regression: y = α + βx + ε, with y = ln(ASR), x = calendar year, where ε is a homoscedastic, mean-zero normal error term and β represents the geometric mean ratio per year. EAPC and its 95% confidence interval (CI) were obtained as EAPC = 100 × (eβ–1). An ASR was deemed to have a statistically significant increasing (or decreasing) trend if the entire 95% CI for EAPC lay above (or below) zero; if the 95% CI included zero, the trend was considered not statistically significant.

*Attributable burden estimation* In GBD, the burden attributable to lack of access to handwashing facilities is estimated using a comparative risk assessment framework. Exposure to 'no access to basic handwashing facilities' is defined as living in a household without a handwashing station with both soap and water available. Relative risks for diarrheal disease associated with this exposure are obtained from meta-analyses of intervention and observational studies. For each age-sex-location-year stratum, population attributable fractions (PAFs) are computed and applied to the underlying diarrheal deaths, YLLs, and YLDs to derive the burden specifically attributable to handwashing. To avoid double‑counting with other WASH risk factors for diarrheal transmission (unsafe water and sanitation), GBD uses a hierarchical attribution structure, and we followed this standard approach in our analyses.

*Model diagnostics* Prior to regression, we confirmed: (1) linearity between ln(ASR) and year via scatterplots, fitted trend lines, and residual diagnostics; (2) independence of residuals using the Durbin–Watson statistic; (3) normality via residual histograms and Q–Q plots; and (4) homoscedasticity via residual-versus-fitted plots. The data satisfied all standard linear-model assumptions. All data satisfied the standard assumptions of the linear regression model.

*Non-linear associations* To explore potential non-linear relationships among the age-standardized incidence rate (ASIR), age-standardized prevalence rate (ASPR), age-standardized death rate (ASDR), age-standardized disability-adjusted life years (DALY) rate, and Socio-Demographic Index (SDI), we employed locally weighted scatterplot smoothing (LOESS) with a smoothing span of 0.75. A tricube weighting kernel was applied, ensuring that observations closer to the target point received greater weight, thus enabling a more flexible depiction of complex, non-linear patterns across various age groups, SDI strata, and Global Burden of Disease (GBD) regions. Software: All analyses were conducted using R (version 4.2.1).

*Forecasting future trends* For global projections of age‑standardized deaths, DALYs, YLDs, and YLLs attributable to lack of handwashing facilities, we fitted autoregressive integrated moving average (ARIMA) models to annual rates from 1990 to 2020. Stationarity was assessed using standard unit‑root tests, and differencing was applied when necessary. Candidate models with different autoregressive (p), differencing (d), and moving average (q) orders were compared using the Akaike information criterion, and the best‑fitting specification for each outcome was selected. We generated forecasts to 2035 with 95% prediction intervals based on parameter uncertainty, treating 2021 as an internal validation year. These projections are intended to illustrate the expected continuation of historical trends under a 'business‑as‑usual' scenario rather than to provide precise predictions.

## Results

### Diarrheal burden and epidemiological trends

From 1990 to 2021, the global health burden attributable to the lack of access to handwashing facilities steadily declined, although the magnitude of this decline varied across different metrics, and the pace of improvement progressively slowed. The global number of deaths decreased from 690,020 (101,778–1,254,779) to 225,455 (30,707–425,941) over this period, with an estimated annual percentage change (EAPC) of −4.89% (−5.14 to −4.64), reflecting an average annual decrease of approximately 4.9% (Table 1). The age-standardized DALY rate decreased from 781.73 (114.54–1402.75) to 183.30 (25.71–330.38) per 100,000, marking a 76.6% reduction and an average annual decline of 4.63% (4.37 to 4.89) (Table 1). Similarly, the age-standardized death rate (ASDR) decreased from 14.20 (2.10–26.04) to 3.04 (0.42–5.64) per 100,000, a cumulative reduction of 78.6% over 31 years, corresponding to an average annual decline of 4.89% (4.64 to 5.14) (Table 2). The age-standardized years of life lost (YLL) rate decreased from 758(111.01–1361.24) to 167(23.48–303.39) per 100,000, a reduction of 77.9% (average − 4.8%(− 5.08 to − 4.52) per year; Table 3). None of these trends showed signs of plateauing, with continued declines through 2021. In contrast, the age-standardized years lived with disability (YLD) rate decreased from 23.45 (3.64− 45.11) to 15.95 (2.51–31.00) per 100,000, a 32% reduction (average − 1.26%(− 1.38 to − 1.14) per year). Globally, the YLD rate fell at a slower pace of − 1.26% (− 1.38 to -1.14) per year, much less than the decline in YLL (4.80% (4.52 to 5.08)). The proportion of DALYs attributable to YLDs increased from approximately 3% in 1990 to over 10% in 2021, reflecting slower improvements in disability outcomes relative to mortality (Table 4).

**Table 1:** The death cases and ASDR of no acccess to handwashing facility in 1990 and 2021, with Temporal Trends from 1990 to 2021

	Deaths cases(95% CI)		ASDR(95% CI)		1990–2021 EAPCs(95%CI)
	1990	2021	1990	2021	
Global	690,020 (101,778–1,254,779)	225,455 (30,707–425,941)	14.20 (2.10–26.04)	3.04 (0.42–5.64)	−4.89 (−5.14 to −4.64)
SDI					
High SDI	275 (32–577)	342 (34–760)	0.04 (0.00–0.08)	0.01 (0.00–0.03)	−1.60 (−2.17 to −1.03)
High-middle SDI	4,532 (520–9,266)	573 (55–1,315)	0.50 (0.06–1.03)	0.04 (0.00–0.09)	−8.27 (−8.60 to −7.93)
Middle SDI	74,901 (9,279–153,739)	14,753 (1,556–30,731)	6.24 (0.80–13.02)	0.70 (0.07–1.43)	−6.87 (−7.03 to −6.72)
Low-middle SDI	351,611 (53,095–642,894)	82,192 (10,353–167,007)	44.67 (6.74–82.63)	6.41 (0.80–13.38)	−6.13 (−6.36 to −5.90)
Low SDI	258,390 (38,992–459,092)	127,465 (17,785–230,283)	64.34 (10.07–117.45)	17.81 (2.51–33.44)	−4.07 (−4.31 to −3.82)
Central Europe, eastern Europe, and central Asia					
Central Asia	1,144 (151–2,389)	162 (20–357)	0.10 (0.08–0.13)	0.04 (0.03–0.05)	−3.24 (−3.55 to −2.93)
Central Europe	45 (4–93)	47 (4–104)	0.06 (0.06–0.07)	0.01 (0.01–0.01)	−5.73 (−6.12 to −5.33)
Eastern Europe	92 (10–205)	11 (1–26)	0.05 (0.01–0.12)	0.01 (0.00–0.01)	−9.16 (−9.87 to −8.45)
High income region					
High-income Asia Pacific	26 (2–63)	48 (4–114)	0.02 (0.00–0.04)	0.01 (0.00–0.02)	−1.67 (−2.02 to −1.32)
High-income North America	7 (0–18)	78 (7–179)	0.00 (0.00–0.01)	0.01 (0.00–0.03)	6.62 (4.80–8.47)
Western Europe	70 (7–153)	255 (26–570)	0.01 (0.00–0.03)	0.02 (0.00–0.05)	2.63 (1.75–3.52)
Australasia	2 (0–5)	3 (0–7)	0.01 (0.00–0.03)	0.01 (0.00–0.01)	−0.30 (−1.48–0.89)
Latin America and Caribbean					
Andean Latin America	1,334 (167–2,620)	124 (14–288)	3.25 (0.41–6.47)	0.21 (0.02–0.49)	−9.02 (−9.32 to −8.73)
Caribbean	3,622 (503–6,547)	1,354 (196–2,556)	9.19 (1.27–16.73)	3.31 (0.49–6.20)	−3.05 (−3.51 to −2.60)
Southern Latin America	78 (7–168)	19 (2–44)	0.17 (0.02–0.37)	0.02 (0.00–0.05)	−5.28 (−5.92 to −4.63)
Tropical Latin America	5,863 (790–11,240)	418 (41–920)	4.24 (0.57–8.12)	0.18 (0.02–0.40)	−9.99 (−10.36 to −9.62)
Central Latin America	6,098 (755–12,217)	710 (87–1,559)	3.87 (0.47–7.75)	0.31 (0.04–0.69)	−7.70 (−8.25 to −7.14)
North Africa and Middle East					
North Africa and Middle East	15,746 (2,153–29,327)	1,797 (228—3,779)	3.49 (0.48–6.67)	0.33 (0.04–0.70)	−7.67 (−7.92 to −7.43)
South Asia					
South Asia	364,099 (54,428–676,173)	89,681 (11,133–194,929)	56.50 (8.37–106.78)	7.40 (0.92–16.11)	−6.38 (−6.57 to −6.20)
Southeast Asia, east Asia, and Oceania					
East Asia	11,078 (1,248–22,763)	164 (16–371)	1.08 (0.12–2.26)	0.01 (0.00–0.03)	−14.69 (−15.17 to −14.22)
Oceania	761 (96–1,407)	751 (97–1,486)	18.66 (2.43–36.67)	8.53 (1.06–16.77)	−2.12 (−2.31 to −1.92)
Southeast Asia	41,193 (5,095–90,813)	5,340 (512–12,891)	12.59 (1.57–29.45)	1.00 (0.10–2.39)	−7.72 (−7.88 to −7.56)
Sub-Saharan Africa					
Central Sub-Saharan Africa	23,483 (3,730–41,541)	8,401 (1,131–15,926)	44.32 (7.27–78.83)	10.95 (1.46–20.95)	−4.46 (−5.16 to −3.76)
Eastern Sub-Saharan Africa	90,734 (14,014–166,662)	44,246 (6,342–78,687)	55.24 (8.81–107.59)	17.65 (2.37–32.58)	−3.76 (−3.91 to −3.61)
Southern Sub-Saharan Africa	9,660 (1,412–17,501)	4,978 (622–9,710)	20.90 (3.16–40.72)	8.25 (1.02–16.25)	−2.69 (−3.02 to −2.36)
Western Sub-Saharan Africa	114,873 (17,431–205,103)	66,860 (9,490–121,797)	56.95 (9.08–106.54)	16.49 (2.44–29.66)	−4.00 (−4.30 to −3.70)

**Table 2:** The DALYs and age-standardized DALY rate of no acccess to handwashing facility in 1990 and 2021, with Temporal Trends from 1990 to 2021

	DALYs(95% CI)		Age-standardized DALY rate(95% CI)			1990–2021 EAPCs(95%CI)
	1990	2021	1990		2021	
Global	45,081,641 (6,585,935–80,545,793)	12,719,108 (1,780,877–22,953,925)	781.73 (114.54–1402.75)		183.30 (25.71–330.38)	−4.63 (−4.89 to −4.37)
SDI						
High SDI	22,263 (2,662–44,504)	10,433 (1,065—22,824)	3.26 (0.39–6.62)	0.88 (0.09–1.96)		−3.41 (−3.71 to −3.11)
High-middle SDI	367,674 (42,362–741,093)	27,312 (2,949—59,149)	40.01 (4.60–80.58)	2.63 (0.28–5.69)		−8.76 (−8.99 to −8.53)
Middle SDI	4,997,672 (622,073–9,873,652)	714,131 (81,009—1,407,793)	288.35 (35.72–579.64)	35.44 (4.05–70.79)		−6.61 (−6.76 to −6.46)
Low-middle SDI	21,299,090 (3,218,980–38,286,228)	3,544,667 (463,365—6,998,970)	1659.44 (250.96–3010.42)	212.65 (27.57–412.06)		−6.44 (−6.68 to −6.21)
Low SDI	18,371,442 (2,723,191–32,430,245)	8,413,698 (1,213,121—15,083,022)	2670.47 (406.93–4785.16)	703.08 (99.37–1247.26)		−4.23 (−4.48 to −3.98)
Central Europe, eastern Europe, and central Asia						
Central Asia	103,811 (13,665–215,713)	14,844 (1,853–32,572)	111.00 (14.61–230.70)	14.97 (1.87–32.82)		−7.37 (−7.78 to −6.97)
Central Europe	3,751 (407–7,635)	1,090 (107–2,384)	4.27 (0.46–8.69)	1.03 (0.10–2.24)		−4.57 (−5.79 to −3.33)
Eastern Europe	10,381 (1,266–22,603)	1,276 (152–2,927)	6.17 (0.75–13.41)	0.82 (0.10–1.87)		−7.25 (−7.66 to −6.85)
High income region						
High-income Asia Pacific	1,855 (213–4,316)	1,574 (160–3,773)	1.29 (0.15–2.97)	0.86 (0.09–2.10)		−0.67 (−0.98 to −0.36)
High-income North America	698 (83–1,680)	1,378 (138–3,176)	0.28 (0.03–0.66)	0.24 (0.02–0.57)		1.33 (0.40–2.28)
Western Europe	5,503 (604–12,255)	6,917 (721–14,854)	1.66 (0.18–3.67)	1.32 (0.13–2.87)		−0.14 (−0.72–0.45)
Australasia	154 (16–358)	82 (8–197)	0.80 (0.09–1.88)	0.22 (0.02–0.52)		−3.76 (−4.25 to −3.28)
Latin America and Caribbean						
Andean Latin America	112,992 (14,048–218,483)	7,815 (899–17,665)	228.13 (28.56–440.23)	12.63 (1.45–28.51)		−9.56 (−9.81 to −9.30)
Caribbean	302,560 (42,394–545,665)	103,691 (15,617–194,680)	728.92 (101.78–1318.50)	263.88 (39.94–495.22)		−2.99 (−3.45 to −2.52)
Southern Latin America	6,633 (690–14,328)	648 (71–1,466)	13.24 (1.38–28.62)	1.04 (0.12–2.35)		−6.96 (−7.33 to −6.59)
Tropical Latin America	490,279 (66,348–937,320)	17,066 (1,717–38,676)	311.95 (42.24–595.79)	8.25 (0.83–18.71)		−11.56 (−11.79 to −11.32)
Central Latin America	469,269 (59,260–944,583)	35,755 (4,483–75,788)	228.06 (28.57–460.94)	16.34 (2.04–34.80)		−8.13 (−8.59 to −7.67)
North Africa and Middle East						
North Africa and Middle East	1,402,129 (191,933–2,623,972)	166,175 (22,594–338,869)	280.48 (38.42–519.46)	28.28 (3.83–57.67)		−7.46 (−7.70 to −7.22)
South Asia						
South Asia	20,316,366 (3,061,932–36,794,866)	3,173,048 (398,862–6,523,499)	1864.52 (279.23–3408.22)	209.83 (26.30–435.73)		−6.84 (−7.02 to −6.66)
Southeast Asia, east Asia, and Oceania						
East Asia	935,649 (108,525–1,889,286)	11,784 (1,348–25,405)	82.60 (9.57–167.20)	1.11 (0.12–2.29)		−14.39 (−14.92 to −13.87)
Oceania	53,683 (6,954–97,896)	49,719 (6,931–102,314)	713.79 (91.27–1318.10)	331.45 (44.82–654.48)		−2.03 (−2.22 to −1.84)
Southeast Asia	2,658,878 (323,101–5,663,629)	235,142 (25,329–514,962)	548.28 (67.35–1185.85)	39.68 (4.24–87.08)		−8.09 (−8.22 to −7.97)
Sub-Saharan Africa						
Central Sub-Saharan Africa	1,848,736 (300,067–3,260,214)	569,343 (82,216–1,084,599)	2147.62 (338.42–3867.62)	421.09 (60.23–775.65)		−5.15 (−5.90 to −4.40)
Eastern Sub-Saharan Africa	6,798,327 (1,010,283–12,535,860)	2,941,673 (440,855–5,162,789)	2479.31 (394.36–4652.75)	686.50 (99.80–1207.23)		−4.18 (−4.31 to −4.06)
Southern Sub-Saharan Africa	717,395 (106,447–1,276,906)	294,919 (40,251–568,594)	1133.79 (167.01–2034.39)	394.50 (53.40–761.63)		−3.12 (−3.48 to −2.76)
Western Sub-Saharan Africa	8,842,582 (1,333,523–15,625,723)	5,085,160 (717,466–9,308,015)	2910.01 (445.54–5173.78)	805.34 (115.81–1450.48)		−4.06 (−4.39 to −3.74)

**Table 3:** The YLLs and age-standardized YLLs rate of no acccess to handwashing facility in 1990 and 2021, with Temporal Trends from 1990 to 2021

	YLLs(95% CI)						Age-standardized YLLs rate(95% CI)					1990–2021 EAPCs(95%CI)
	1990		2021				1990		2021			
Global	43,734,491 (6,382,801–77,973,309)		11,497,773 (1,603,941–20,825,427)				758.28 (111.01–1361.24)		167.35 (23.48–303.39)			−4.80 (−5.08 to −4.52)
SDI												
High SDI	15,293 (1,757–33,189)		5,379 (528–11,943)			2.34 (0.27–5.07)		0.35 (0.03–0.78)			−5.33 (−5.74 to −4.93)	
High-middle SDI	330,276 (37,816–668,002)		16,624 (1,707–37,340)			36.22 (4.14–73.38)		1.65 (0.17–3.56)			−9.98 (−10.24 to −9.73)	
Middle SDI	4,710,168 (584,306–9,285,915)		570,773 (63,411–1,146,765)			272.76 (33.66–551.58)		29.34 (3.30–58.67)			−7.00 (−7.17 to −6.82)	
Low-middle SDI	20,682,291 (3,124,264–37,170,067)		3,059,337 (396,117–5,786,558)			1612.76 (243.81–2921.99)		187.42 (24.07–358.27)			−6.71 (−6.97 to −6.44)	
Low SDI	17,973,547 (2,657,831–31,720,862)		7,837,357 (1,118,526–14,148,761)			2604.99 (396.21–4669.27)		652.51 (91.20–1176.32)			−4.36 (−4.63 to −4.10)	
Central Europe, eastern Europe, and central Asia												
Central Asia	100,525 (13,260–209,482)		13,993 (1,735–30,985)			107.24 (14.14–223.71)		14.08 (1.75–31.17)			−7.49 (−7.90 to −7.08)	
Central Europe	3,470 (377–7,096)		1,022 (99–2,248)			3.98 (0.43–8.13)		0.96 (0.09–2.09)			−4.61 (−6.03 to −3.17)	
Eastern Europe	6,871 (781–15,209)		430 (48–979)			4.26 (0.48–9.43)		0.29 (0.03–0.65)			−10.09 (−10.77 to −9.41)	
High income region												
High-income Asia Pacific	705 (76–1,682)		640 (59–1,521)		0.52 (0.06–1.23)			0.18 (0.02–0.43)			−2.56 (−2.86 to −2.26)	
High-income North America	279 (33–676)		1,317 (131–3,035)		0.11 (0.01–0.27)			0.23 (0.02–0.53)			3.46 (2.28–4.65)	
Western Europe	1,347 (144–2,921)		3,323 (342–7,372)		0.35 (0.04–0.76)			0.40 (0.04–0.87)			1.63 (0.79–2.48)	
Australasia	54 (5–125)		48 (4–115)		0.29 (0.03–0.67)			0.11 (0.01–0.26)			−0.85 (−2.02–0.33)	
Latin America and Caribbean												
Andean Latin America	103,547 (12,856–203,920)		5,991 (680–13,544)		207.00 (25.77–407.67)			9.83 (1.12–22.14)			−9.96 (−10.23 to −9.68)	
Caribbean	298,574 (41,811–538,927)		100,784 (15,187–188,208)		718.56 (100.26–1299.17)			257.37 (38.98–480.63)			−3.04 (−3.51 to −2.56)	
Southern Latin America	4,701 (452–10,120)		431 (47–970)		9.43 (0.91–20.32)			0.68 (0.08–1.54)			−7.44 (−8.02 to −6.86)	
Tropical Latin America	470,016 (63,391–905,299)		12,427 (1,188–27,347)		299.61 (40.41–576.24)			6.04 (0.57–13.34)			−12.26 (−12.47 to −12.06)	
Central Latin America	449,990 (56,556–903,595)		30,589 (3,786–66,048)		217.69 (27.14–436.54)			14.23 (1.75–30.65)			−8.38 (−8.86 to −7.90)	
North Africa and Middle East												
North Africa and Middle East	1,354,335 (185,131–2,550,542)		142,710 (18,721–295,832)		270.32 (36.98–504.49)			24.56 (3.20–50.91)			−7.83 (−8.10 to −7.57)	
South Asia												
South Asia	19,686,522 (2,968,916–35,624,242)		2,634,697 (329,545–5,530,138)	1812.41 (271.53–3314.28)				180.48 (22.51–375.96)				
Southeast Asia, east Asia, and Oceania												
East Asia	871,377 (100,180–1,782,488)	6,037 (598–13,090)		77.18 (8.86–158.14)				0.66 (0.07–1.40)			−15.80 (−16.32 to −15.27)	
Oceania	51,425 (6,612–95,133)	46,141 (6,315–95,622)		683.92 (86.71–1271.09)				307.38 (40.63–622.33)			−2.09 (−2.29 to −1.89)	
Southeast Asia	2,562,825 (307,300–5,446,775)	195,671 (20,202–431,046)		529.58 (64.21–1149.88)				33.66 (3.46–74.42)			−8.48 (−8.60 to −8.37)	
Sub-Saharan Africa												
Central Sub-Saharan Africa	1,811,977 (293,381–3,200,137)		508,661 (69,944–997,347)	2097.48 (329.28–3785.68)				378.42 (50.88–712.66)		−5.37 (−6.16 to −4.57)		
Eastern Sub-Saharan Africa	6,622,537 (979,951–12,198,532)		2,686,157 (396,728–4,785,655)	2404.30 (381.45–4535.53)				628.11 (89.80–1116.05)		−4.35 (−4.48 to −4.21)		
Southern Sub-Saharan Africa	672,933 (99,422–1,207,592)		263,871 (35,042–514,439)	1058.01 (155.04–1914.78)				356.54 (47.08–695.32)		−3.20 (−3.58 to −2.81)		
Western Sub-Saharan Africa	8,660,470 (1,300,634–15,359,201)		4,842,823 (676,202–8,932,207)	2830.36 (431.15–5051.06)				758.20 (107.75–1378.46)		−4.15 (−4.48 to −3.81)

**Table 4:** The YLDs and age-standardized YLDs rate of no acccess to handwashing facility in 1990 and 2021, with Temporal Trends from 1990 to 2021

	YLDs(95% CI)				Age-standardized YLDs rate(95% CI)											1990–2021 EAPCs(95%CI)
	1990	2021			1990					2021						
Global	1,347,149 (209,551–2,609,686)	1,221,335 (192,400–2,368,561)			23.45 (3.64–45.11)					15.95 (2.51–31.00)						−1.26 (−11.38 to −11.14)
SDI																
High SDI	6,969 (844–15,421)		5,053 (523–11,529)		0.92 (0.11–2.05)				0.53 (0.05–1.18)						−11.21 (−11.60 to −110.81)	
High-middle SDI	37,397 (4,690–80,508)		10,687 (1,284–24,212)		3.78 (0.47–8.15)				0.97 (0.11–2.19)						−14.30 (−14.43 to −14.18)	
Middle SDI	287,503 (41,099–577,332)		143,357 (18,937–297,820)		15.59 (2.26–31.35)				6.10 (0.81–12.62)						−13.09 (−13.19 to −12.99)	
Low-middle SDI	616,799 (96,895–1,170,257)		485,329 (73,558–963,256)		46.68 (7.35–88.48)				25.23 (3.83–50.40)						−12.05 (−12.15 to −11.95)	
Low SDI	397,895 (67,850–729,264)		576,341 (97,690–1,080,462)		65.49 (11.11–119.98)				50.57 (8.58–95.95)						−10.91 (−11.00 to −10.82)	
Central Europe, eastern Europe, and central Asia																
Central Asia	3,285 (406–6,903)		851 (116–1,920)		3.75 (0.46–7.86)				0.89 (0.12–2.00)					−14.80 (−15.14 to −14.46)		
Central Europe	281 (33–626)		67 (7–151)		0.29 (0.03–0.65)				0.07 (0.01–0.16)					−14.37 (−14.56 to −14.17)		
Eastern Europe	3,510 (428–8,059)		845 (101—1,962)		1.91 (0.23–4.37)				0.52 (0.06–1.22)					−13.97 (−14.28 to −13.65)		
High income region																
High-income Asia Pacific	1,149 (128–2,806)		934 (92—2,294)		0.76 (0.08–1.87)				0.68 (0.07–1.67)					0.14 (−10.21–0.50)		
High-income North America	419 (49–1,039)		60 (6—143)		0.16 (0.02–0.41)				0.02 (0.00–0.04)					−17.02 (−17.86 to −16.17)		
Western Europe	4,155 (462–9,479)		3,593 (372–8,176)		1.31 (0.14–3.03)				0.92 (0.10–2.11)					−10.68 (−11.25 to −10.10)		
Australasia	99 (11–233)		34 (3–83)		0.51 (0.06–1.22)				0.11 (0.01–0.26)					−15.32 (−16.11 to −14.53)		
Latin America and Caribbean																
Andean Latin America	9,444 (1,221–20,056)		1,824 (221–4,117)		21.13 (2.73–44.81)			2.80 (0.34–6.29)					−17.18 (−17.63 to −16.74)			
Caribbean	3,986 (624–7,939)		2,906 (406–5,832)		10.36 (1.62–20.67)			6.51 (0.91–13.01)					−10.76 (−11.44 to −10.07)			
Southern Latin America	1,931 (216–4,456)		217 (24–512)		3.81 (0.43–8.80)			0.35 (0.04–0.83)					−16.42 (−17.42 to −15.42)			
Tropical Latin America	20,262 (3,216–43,702)		4,638 (512–11,150)		12.34 (1.96–26.48)			2.21 (0.24–5.35)					−16.14 (−16.40 to −15.88)			
Central Latin America	19,278 (2,634–41,076)		5,166 (690–11,611)		10.37 (1.42–22.26)			2.12 (0.28–4.76)					−15.39 (−15.60 to −15.18)			
North Africa and Middle East																
North Africa and Middle East	47,794 (6,583–99,565)		23,465 (3,208–52,277)	10.16 (1.40–21.22)			3.71 (0.51–8.27)					−12.65 (−12.94 to −12.36)				
South Asia																
South Asia	629,844 (99,059–1,205,775)		538,350 (77,911–1,108,302)	52.11 (8.19–99.49)			29.35 (4.25–60.36)					−11.92 (−11.97 to −11.87)				
Southeast Asia, east Asia, and Oceania																
East Asia	64,272 (8,377–136,203)	5,747 (702–12,879)		5.43 (0.71–11.48)		0.45 (0.05–1.00)					−18.58 (−18.99 to −18.17)					
Oceania	2,258 (364–4,307)	3,578 (568–7,064)		29.88 (4.82–57.26)		24.07 (3.82–47.29)					−10.97 (−11.04 to −10.90)					
Southeast Asia	96,053 (13,131–199,830)	39,471 (4,904–84,297)		18.70 (2.55–38.58)		6.02 (0.74–12.86)					−13.53 (−13.64 to −13.41)					
Sub-Saharan Africa																
Central Sub-Saharan Africa	36,758 (6,363–66,980)	60,681 (10,456–113,226)		50.14 (8.72–91.44)		42.67 (7.49–79.45)					−10.55 (−10.68 to −10.42)					
Eastern Sub-Saharan Africa	175,789 (30,725–317,386)	255,516 (44,217–468,316)		75.02 (13.26–135.91)		58.40 (10.25–108.09)					−10.91 (−10.99 to −10.83)					
Southern Sub-Saharan Africa	44,461 (6,928–86,242)	31,048 (4,619–62,941)		75.78 (11.92–146.05)		37.95 (5.66–77.04)					−12.24 (−12.33 to −12.14)					
Western Sub-Saharan Africa	182,111 (31,585–331,747)	242,336 (41,208–448,480)		79.64 (14.01–145.27)		47.15 (7.93–87.08)					−11.91 (−12.02 to −11.79)					

Stratification by SDI revealed substantial disparities. In 2021, countries with high and high-middle SDI recorded age-standardized death rates (ASDR) of less than 0.5 per 100,000, with average annual declines ranging from 7 to 9%. Middle-SDI countries had an ASDR of 6.24 (0.80–13.02) per 100,000, with an average annual decline of approximately 6.87% (6.72 to 7.03); low-middle SDI countries had an ASDR of 6.41 (0.80–13.38) per 100,000 (average decline ~ 6.13% (5.90 to 6.36)); and low-SDI countries had an ASDR of 17.8(2.51–33.44) per 100,000, with an average annual decline of around 4.07% (3.82 to 4.31), approximately two-thirds of the global mean. The gap between low-SDI countries and the global average widened from 3.8-fold in 1990 to 5.9-fold in 2021 (Table 1).

**Fig. 1:**
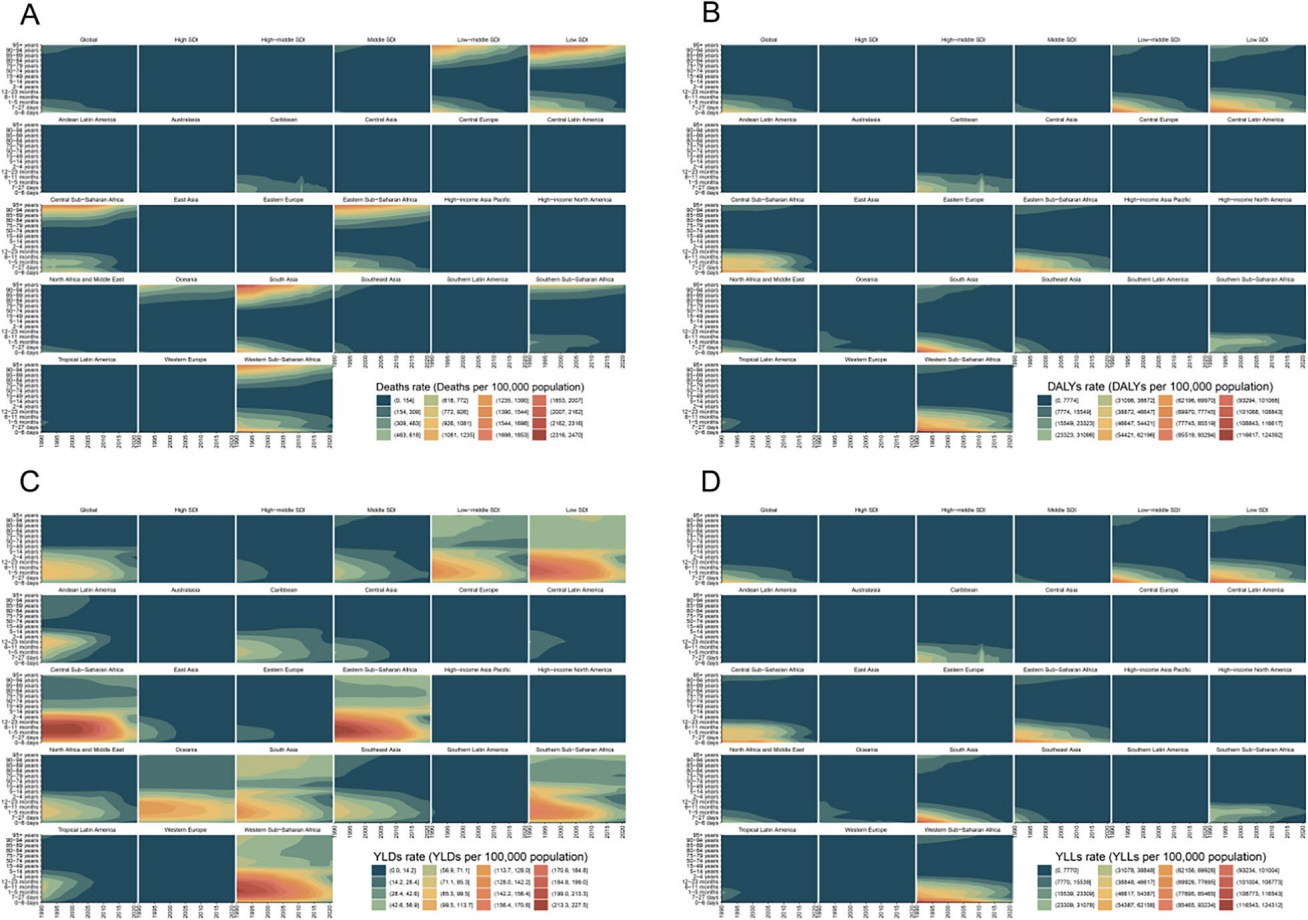
A comparative analysis of the distribution characteristics of no access to handwashing facility deaths rate, disability-adjusted life years (DALY) rate, YLDs rate, YLLs rate, and across different sociodemographic index (SDI) regions and global burden of disease (GBD) regions was performed. deaths rate (**A**), DALYs rate (**B**), YLDs rate (**C**) and YLLs rate (D) across different age groups in various SDI regions and GBD regions

By age, all groups experienced improvements, with declines in DALYs, deaths, YLDs, and YLLs across all regions. The largest relative reductions were observed in infants and young children. However, absolute rates remained highest in older adults, and population aging led to an increase in both the absolute number and share of deaths among the elderly. Mortality was highest among individuals aged ≥ 75 years, followed by children aged < 5 years, with infants under 1 year exhibiting particularly high mortality. Among individuals aged 5–74 years, death rates and rates of DALYs, YLDs, and YLLs were the lowest. (Fig. 1A–D).

**Fig. 2:**
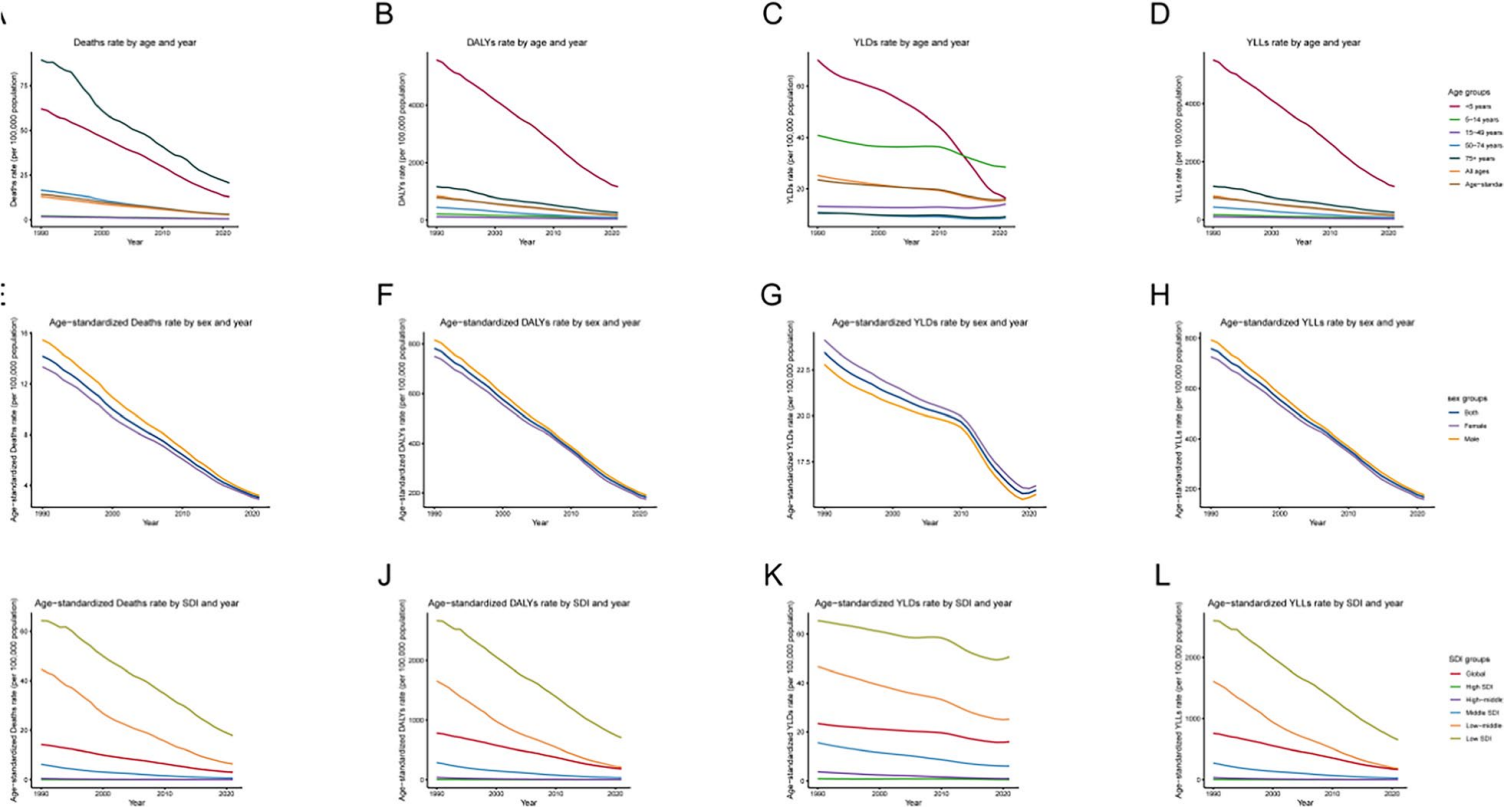
The change trends of age-standardized rates of no access to handwashing facility different SDI quintiles and sex: the Deaths rate from 1990 to 2021 and the YLDs rate from 1990 to 2021 and the YLLs rate from 1990 to 2021 and the age-standardized Deaths rate from 1990 to 2021 and the age-standardized YLDs rate from 1990 to 2021 and the age-standardized YLDs rate from 1990 to 2021

Sex patterns remained consistent over the three decades: males exhibited higher mortality, DALYs, and YLLs compared to females, while females had slightly higher YLD rates. In recent years, the mortality and DALY rates for both males and females have converged, with clear downward trends from 1990 to 2021 (Fig. 2E–H).

### Age-specific burden and prevalence trends by SDI region

Globally, the burden of diarrheal diseases attributable to a lack of access to handwashing facilities declined significantly, with substantial reductions in both the number of deaths and the age-standardized death rate (ASDR). The estimated annual percentage changes (EAPCs) were negative across all Socio-Demographic Index (SDI) strata, indicating improvements at all levels of development.

In high-SDI regions, both deaths and the ASDR remained low, showing minimal changes over time. From 1990 to 2021, the number of deaths increased slightly from 275 (32–577) to 342(34–760), while the ASDR decreased from 0.04 (0.00–0.08) to 0.01 (0.00–0.03) per 100,000, reflecting the effectiveness of public health measures. The modest increase in deaths likely relates to population aging and improved case ascertainment. In high‑SDI regions, absolute numbers of attributable deaths remained very low throughout the study period; therefore, estimates for some age-sex strata should be interpreted with caution.

In high-middle SDI regions, deaths decreased sharply from 4,532(520–9,266) to 573 (55–1,315), and the ASDR dropped from 0.50 (0.06–1.03) to 0.04(0.00–0.09), with an EAPC of -8.27% (95% CI − 8.60 to − 7.93), indicating significant progress in improving access to handwashing facilities.

In middle-SDI regions, deaths fell from 74,901(9,279–153,739) to 14,753(1,556–30,731), and the ASDR dropped from 6.24(0.80–13.02) to 0.70 (0.07–1.43), with an EAPC of − 6.87% (95% CI − 7.03 to − 6.72), signaling substantial, though still incomplete, improvement.

In low-middle and low-SDI regions, both deaths and ASDRs also declined, but from higher baselines and with relatively smaller proportional gains. Specifically, in low-middle SDI regions, deaths fell from 351,611(53,095–642,894) to 82,192 (10,353–167,007), and the ASDR decreased from 44.67 (6.74–82.63) to 6.41 (0.80–13.38). In low-SDI regions, deaths declined from 258,390 (38,992–459,092) to 127,465(17,785–230,283), and the ASDR decreased from 64.34 (10.07–117.45) to 17.81(2.51–33.44). Although low-SDI regions saw the largest absolute declines, their residual burden remains the highest, warranting intensified public health action. In low-middle and middle-SDI regions, while declines were notable, ASDRs remain elevated, requiring continued attention (Fig. 1).

By age, the prevalence attributable to inadequate handwashing facilities declined over time across all groups, with the extent of reduction varying according to the Socio‑Demographic Index (SDI). The decrease was more pronounced in high and high‑middle SDI regions, while progress was slower in low and low‑middle SDI regions. Children under 5 and those aged 5–14 years faced the highest risk, particularly in low and low‑middle SDI settings. As SDI rises, childhood diarrheal mortality drops significantly, underscoring the crucial role of socioeconomic development and improved public health conditions in protecting child health.Among adults aged 15–49 and 50–74 years, mortality rates remained relatively low, though the issue continues to be a notable concern in low and low‑middle SDI regions. For individuals aged 75 years and older, mortality was relatively high across all SDI categories, yet decreased with higher SDI levels—highlighting the need for enhanced public health infrastructure and clinical care for the elderly. (Fig. 1A–D).

**Fig. 3:**
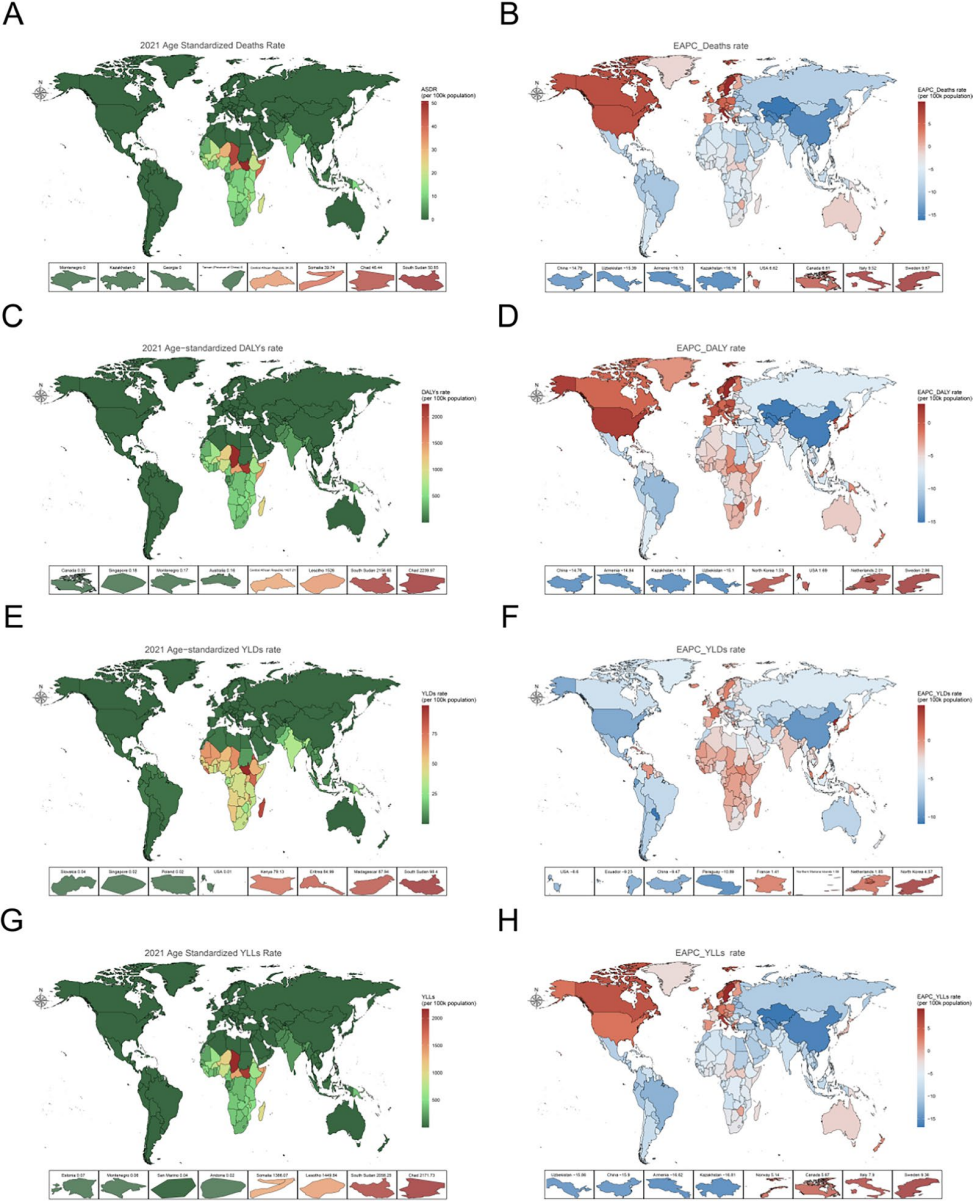
The age-standardized rates of no access to handwashing facility in 204 countries or territories in 2021: (**A**) The deaths rate of 204 countries or territories in 2021; (**B**) The EAPCs of deaths rate in 2021; (**C**) the age-standardized DALYs rate of 204 countries or territories in 2021; (**D**) the EAPCs of DALYs rate in 2021; (**E**) the YLDs rate of 204 countries or territories in 2021; (F) the EAPCs of YLDs in 2021; (**G**) the YLLs rate of 204 countries or territories in 2021; (**H**) the EAPCs of YLLs in 2021;. DALYs: disability-adjusted life years; YLDs: years lived with disability;YLL:yearsof life lost;EAPCs: estimated annual percentage changes

**Fig. 4:**
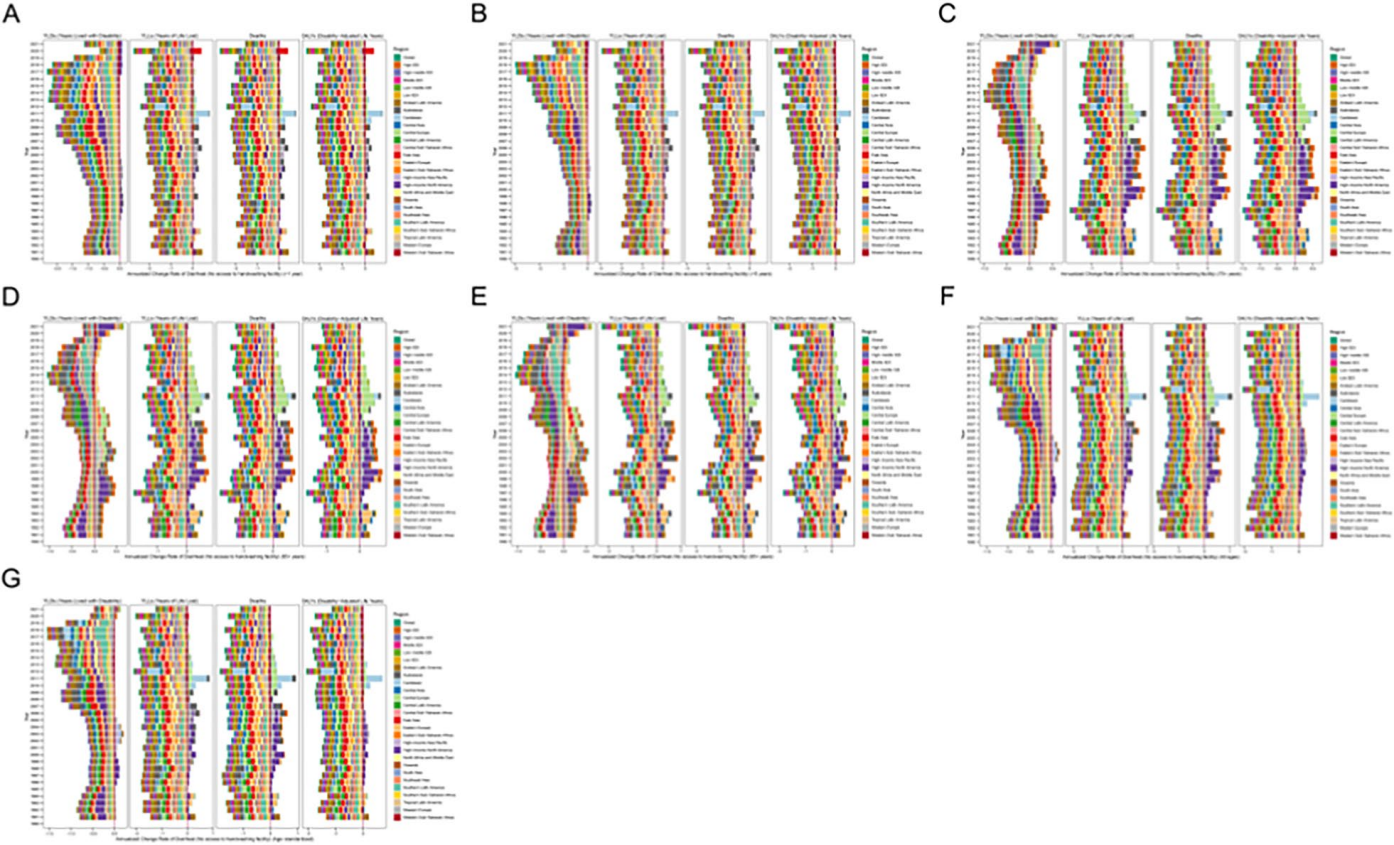
Annualized change rate of no access to handwashing facility incidence among population grouped by age across regions from 1990 to 2021. Annualized change rate of no access to handwashing facility incidence among population under 1 year of age (**A**), under 5 years of age (**B**), above 75 years of age (**C**) and above 85 years of age (**D**) and above 95 years of age (**E**) and age-standardized (**F**). DALYs, disability-adjusted life-years

Geographic patterns further confirm the concentration of the burden associated with the lack of handwashing facilities. In 2021, regions in Africa, South Asia, and Southeast Asia exhibited notably higher age-standardized rates for death, DALY (Disability-Adjusted Life Years), YLD (Years Lived with Disability), and YLL (Years of Life Lost), with certain locations showing positive EAPCs (Estimated Annual Percent Change), indicating either deterioration or very slow improvement, reflecting a persistently heavy burden. In contrast, high-income regions in North America, Europe, and Oceania showed more favorable indicators, with comparatively low mortality and DALY rates (Fig. 3B, D, F, H). High-SDI regions generally displayed stable or slowly improving trends, with Australia, West Asia, and high-income North America showing modest changes across most metrics. However, low-SDI regions—such as sub-Saharan Africa and South Asia—demonstrated greater volatility and overall poorer performance (Fig. 4A–G).

### Regional disparities in diarrheal burden attributable to lack of handwashing facilities

Although the global burden of diarrhea attributable to inadequate access to handwashing facilities has decreased substantially, GBD 2021 reveals pronounced heterogeneity across regions. Both absolute burden and rates of decline are highly uneven, highlighting significant geographic inequities.

Regional differences in mortality are particularly striking. In 2021, South Asia reported 89,681 (11,133–194,929) deaths, representing the largest share of the global mortality and DALY burden (Table 1). In terms of age-standardized death rates (ASDR), West Africa (16.49 (2.44–29.66) per 100,000) and East Africa (17.65(2.37–32.58) per 100,000) were the highest. Tropical Latin America experienced the fastest decline (EAPC − 9.99 (− 10.36 to − 9.62)), whereas high-income North America showed a positive EAPC (+ 6.62 (4.80–8.47)) from a very low baseline (Table 1). Using the 2021 age-standardized DALY rate as a benchmark, the 21 regions can be classified into four tiers:

- (1) Very high burden (> 600 per 100,000): West Africa, East Africa
- (2) High burden (200–600 per 100,000): Central Africa, South Asia
- (3) Moderate burden (50–200 per 100,000): Andean Latin America, the Caribbean, Southeast Asia, Central Latin America, North Africa/Middle East, Oceania
- (4) Low burden (< 50 per 100,000): Central Asia, Central Europe, Eastern Europe, all high-income regions, East Asia

Disparities have remained substantial over time. The DALY-rate gap between West Africa and Western Europe was 55-fold in 1990 and still 52-fold in 2021. While global deaths fell by 67%, West Africa’s decline was only 42%, causing its share of global deaths to rise from 17 to 30%.

These burden patterns are mirrored in YLL and YLD metrics. In 2021, global YLLs totaled 11,497,773 (1,603,941–20,825,427), accounting for 90.4% of DALYs. West Africa exhibited the highest YLL rate (758 (107.75–1378.46) per 100,000), whereas high-income North America had a YLL rate of 0.23 (0.02–0.53) per 100,000 but showed an increasing trend (+ 3.46 (2.28–4.65)), consistent with the mortality data. Global YLDs were 1,221,335 (192,400–2,368,561) in 2021, with an age-standardized rate of 15.95 (2.51–31.00) per 100,000 and an average annual decline of 1.26% (1.14 to 1.38) over 31 years. Only West and East Africa experienced annual increases in YLD rates (≈0.5–0.9%), likely reflecting population growth and a rising prevalence of post-diarrheal sequelae in some countries (Tables 3 and 4). All other regions experienced declines, with the largest reduction observed in East Asia (− 8.58% (− 8.99 to − 8.17)). These patterns underscore differences in access to handwashing facilities, demographic dynamics, and the intensity of interventions across regions.

Overall, from 1990 to 2021, the global burden attributable to the lack of handwashing facilities decreased significantly, but regional disparities remain substantial. Western and Eastern Sub-Saharan Africa continue to experience the highest per-capita mortality and disability-adjusted life year (DALY) burdens, with DALY rates exceeding 600 per 100,000 as of 2021 (Tables 1 and 2). Notably, South Asia accounts for the largest number of absolute cases, suggesting that current interventions may not adequately reach high-risk populations. While the burden is minimal in high-income regions, the recent rebound in deaths and years of life lost (YLL) in North America indicates the need for continued surveillance of vulnerable groups.

### Country- and territory-level trends in diarrheal burden attributable to lack of handwashing facilities

**Fig. 5:**
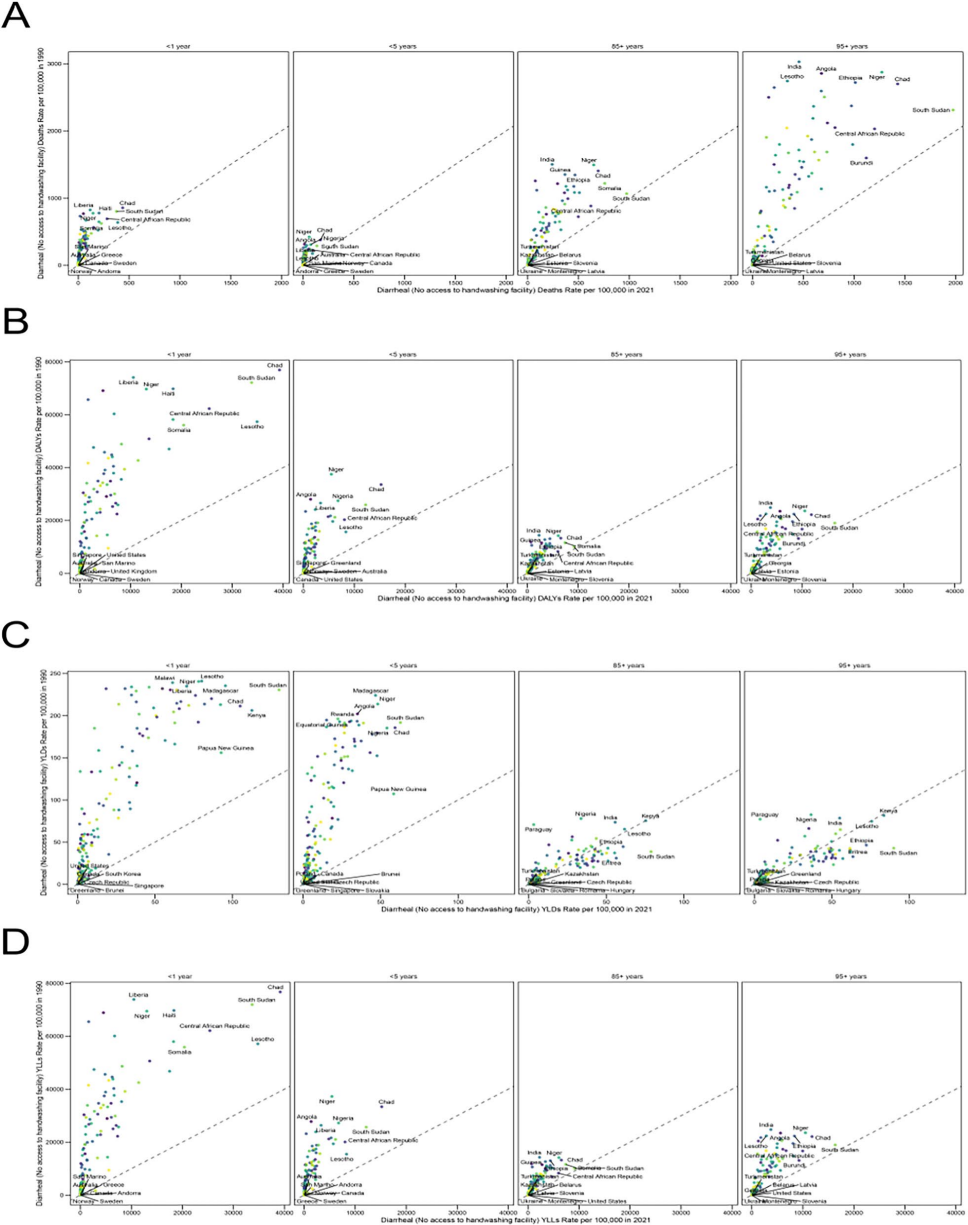
Comparison of trends in no access to handwashing facility deaths, DALYs, YLDs, YLLs rates across different age groups in multiple countries between 1990 and 2021. The death rates (**A**), DALY rates (**B**), YLD rates (**C**) and YLL rates (**D**) of no access to handwashing facility in different age groups and regions in 1990 and 2021 per 100,000 people. DALYs, disability-adjusted life-years

**Fig. 6:**
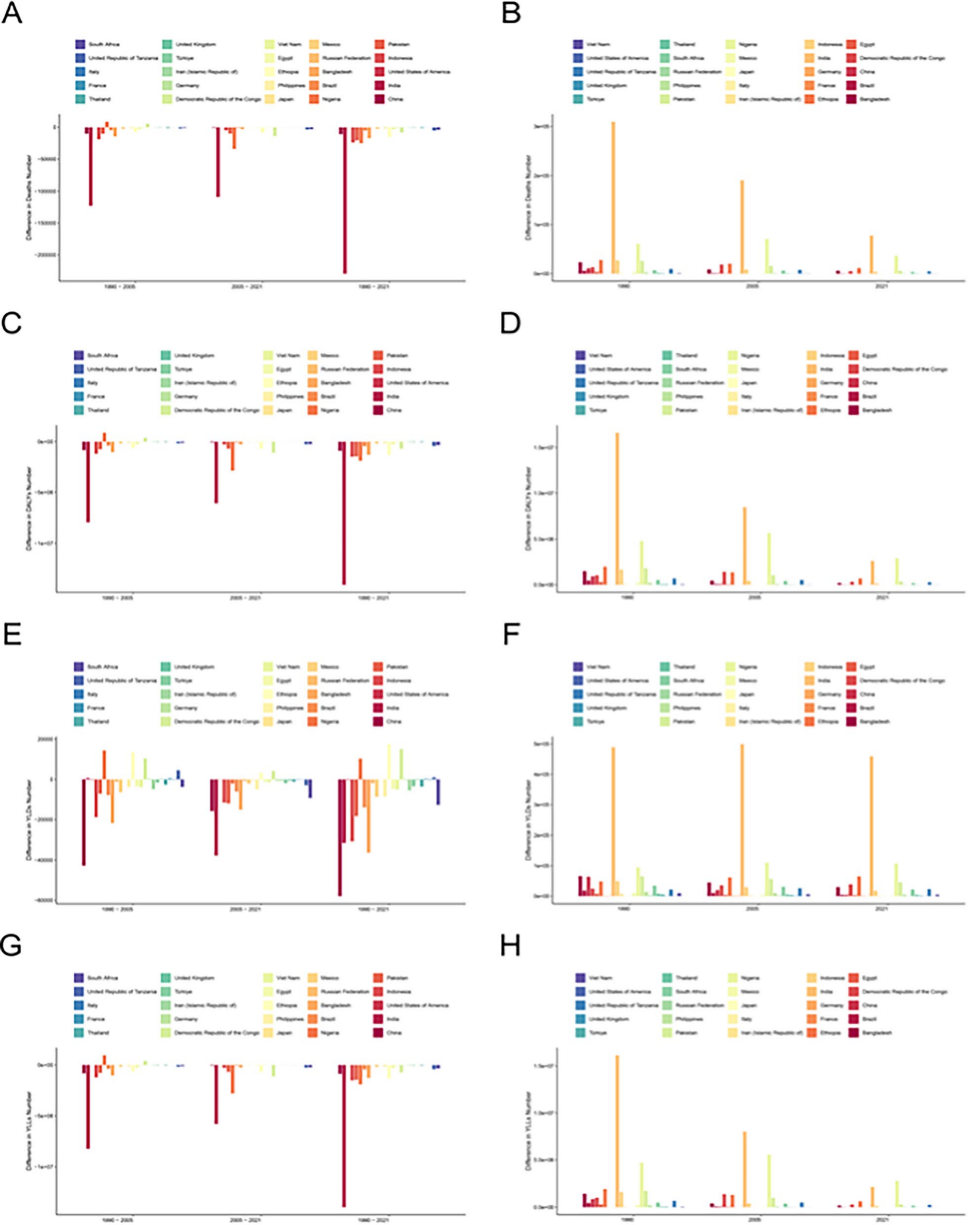
Deaths, DALYs, YLDs, and YLLs number in the 25 most populous countries in 1990, 2005, and 2021, and absolute changes from 1990 to 2005, 2005 to 2018, and 1990 to 2021. Deaths number in the 25 most populous countries absolute changes from 1990 to 2005, 2005 to 2018, and 1990 to 2021(**A**), Deaths number in the 25 most populous countries in 1990, 2005, and 2021(**B**),DALYs number in the 25 most populous countries absolute changes from 1990 to 2005, 2005 to 2018, and 1990 to 2021(**C**), DALYs number in the 25 most populous countries in 1990, 2005, and 2021(**D**),YLDs number in the 25 most populous countries absolute changes from 1990 to 2005, 2005 to 2018, and 1990 to 2021(**E**), YLDs number in the 25 most populous countries in 1990, 2005, and 2021(**F**),YLLS number in the 25 most populous countries absolute changes from 1990 to 2005, 2005 to 2018, and 1990 to 2021(**G**), YLLs number in the 25 most populous countries in 1990, 2005, and 2021(H). DALYs, disability-adjusted life-years

The Global Burden of Disease (GBD) 2021 report reveals that 99 countries/territories reported indicators related to diarrhea attributable to a lack of handwashing facilities between 1990 and 2021 (Supplementary Tables S1–S4; Figs. 5 and 6). This provides a comprehensive dataset for characterizing epidemiological patterns across regions.

At the national level, countries can be grouped into three distinct trajectories with respect to deaths and overall burden:

- *(1) Rapid Convergence* This pattern is observed in countries such as China, Brazil, Iran, Vietnam, and Peru, where deaths and YLLs decreased by over 90%, with estimated annual percentage changes (EAPCs) typically ranging from -9% to -15%. For example, in China, deaths dropped from 934,019 (108,347–1,886,528) in 1990 to 10,318 (1,108–22,067) in 2021, while the age-standardized death rate (ASDR) decreased from 85.51 (9.91–173.13) to 1.02 (0.11–2.11) per 100,000 (Supplementary Table S1).
- *(2) High Baseline—Slow Decline* This trend is seen in many sub-Saharan African countries (e.g., Niger, Chad, Nigeria, the Democratic Republic of the Congo, Madagascar), where the ASDRs in 1990 often exceeded 1,000–3,000 per 100,000 and remained above 200–800 per 100,000 in 2021. The EAPCs in these regions were generally between -2% and -4%, well below the global average. For instance, in Niger, DALYs still totaled 2.9 million in 2021, with an ASR exceeding 900 per 100,000—comparable to China’s 1990 level (Table S1).
- *(3) Low Baseline—Fluctuating Trend (High-Income OECD)* Represented by countries such as the United States, the United Kingdom, Germany, Japan, and Australia, where ASDRs were already low (< 5–20 per 100,000) in 1990 and remained mostly below 1–3 per 100,000 by 2021. EAPCs followed a "U-shaped" trajectory: a rapid decline between 1990 and 2010, followed by a deceleration or partial rebound thereafter (Supplementary Table S1). For example, in the United States, there were 52 (6–124) deaths in 2021, with an ASDR of 1.4 (0.17–3.36) per 100,000, but years lived with disability (YLD) declined by only -5.19% (-6.35 to -4.01), suggesting that “mild/recurrent” illnesses continue to impact vulnerable populations.

Trends in DALYs, YLDs, and YLLs generally followed the same patterns as death rates, although with varying magnitudes. Several countries experienced significant reductions in deaths during both the 1990–2005 and 2005–2021 periods. For instance, some European countries, such as Germany, maintained low and stable death rates, with progressively smaller—and sometimes negative—changes in death counts over time. In contrast, countries like Pakistan and Egypt saw more considerable fluctuations between periods, reflecting challenges such as uneven economic development, regional disparities, and inconsistent health policy implementation. These factors collectively hindered progress in improving sanitation. In nations such as Chad, the Central African Republic, and South Sudan, mortality rates remained high, with 2021 death rates showing little to no improvement—or even increases—compared to 1990. Notably, regions such as East Asia, Oceania, and Southeast Asia experienced significant declines in mortality rates (Fig. 6).

When geographic distribution is analyzed alongside age- and sex-stratified data, clearer patterns emerge. Overall, children under 5 years old accounted for 68% of DALYs, with boys contributing 55% and girls 45% (Table S1). For example, using DALY rates from 2021, the curve for ages 0–1 declines steeply, with male rates consistently higher than female rates throughout early childhood. The largest sex gap occurs during the neonatal period. After age 5, the curves “cross,” with female DALY rates surpassing male rates, and this trend continues into older adulthood. By age 65 and beyond, DALY rates increase, and since women generally have longer life expectancy, their absolute numbers are higher (Supplementary S2).

YLDs exhibit a bimodal distribution, with school-age children (10–14 years) and women aged 30–39 years contributing approximately 12% and 19% of global YLDs, respectively (Table S3). These patterns indicate that women in their reproductive years (approximately 15–49 years) account for a substantial share of disability related to diarrheal disease attributable to lack of handwashing facilities.

Integrating country, age, and sex dimensions results in four illustrative profiles: *High Burden + High Fertility (e.g., Niger)* In Niger, children aged 0–4 years had mortality rates of 2,360 per 100,000 in males and 1,980 per 100,000 in females, and together they accounted for approximately 88% of all diarrheal deaths attributable to lack of handwashing facilities across all age groups in 2021. Among women aged 15–49 years, the Disability-Adjusted Life Year (DALY) rate was 1,180 per 100,000—seven times the global mean for the same age group (see Supplementary Table S2). *Rapid Decline + Narrowing Sex Gap (e.g., India)* From 1990 to 2021, the male infant age-standardized death rate (ASDR) decreased from 1,260 to 82 per 100,000 (− 12.3%/year), while the female child ASDR declined at − 12.0%/year. The male-to-female ratio shrank from 1.40 to 1.12. However, among women aged 15–49 years, the Years Lived with Disability (YLD) rate declined by only -3.2%/year, compared to a − 4.6%/year reduction in men (see Tables 2 and 3), indicating a narrowing “mortality gap” but a persistent pattern of higher disability in females than in males. *Plateau + Emerging Older-Age Burden (e.g., China)* Mortality in children aged 0–4 years is now less than 1 per 100,000, yet the share of deaths among those aged ≥ 70 years has risen from 3% in 1990 to 19% in 2021. Mortality in older men is 1.6 times higher than in older women, and YLD shows a secondary peak among women aged 15–49 years. With pediatric issues largely controlled, hand-hygiene interventions should shift to a “dual-end” strategy targeting both the very young and the elderly (see Tables S1–S3 and Fig. 6). *Low Overall Burden + Vulnerable Subgroups (e.g., United States)* The national age-standardized death rate (ASDR) is 14.20 (2.10—26.04) per 100,000; the DALY rate is 1.3; and the YLD rate is 0.7 (higher in females), consistent with global trends (see Tables 1–3), indicating substantial heterogeneity within “low-burden” countries.

**Fig. 7:**
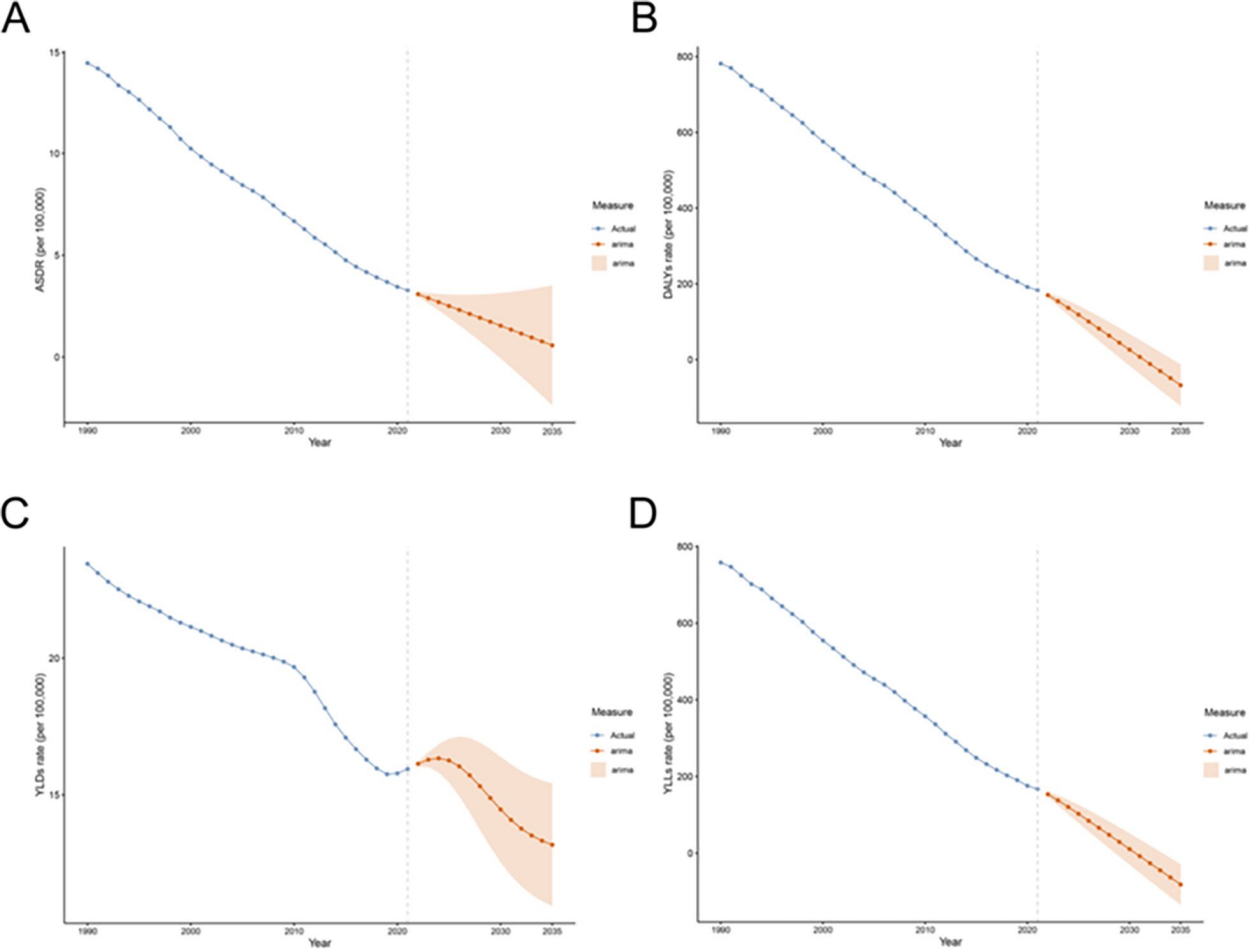
The trends and projected numbers of no access to handwashing facility from 1990 to 2050 at the global level. (**A**) ASDR; (**B**) DALYs rate; (**C**) YLDs rate; (**D**) YLLs rate

From 1990 to 2020, indicators related to diarrhea due to insufficient handwashing facilities declined steadily at a relatively consistent pace, though further gains may be harder to achieve. ARIMA-based forecasts suggest continued reductions through 2035, albeit at a decelerating rate with widening uncertainty bands. Specifically, the ASDR is projected to continue decreasing, though more slowly (see Fig. 7A), with prediction intervals (orange shading) expanding over time. DALY rates are expected to decrease further, again with slowing improvement and increasing uncertainty (see Fig. 7B). YLD rates are projected to re-enter a downward trajectory after 2020, but with less stability and wider intervals, suggesting increased sensitivity to factors such as population aging and emerging threats (see Fig. 7C). Years of Life Lost (YLL) rates should also continue to decline, albeit at a reduced pace and with growing uncertainty, indicating that further reductions in premature mortality will require additional efforts and resources (see Fig. 7D).

When comparing country‑level access to basic handwashing facilities with age‑standardized diarrheal death rates in 2021, we observed the expected pattern of higher burden in settings with poorer access. However, a small number of countries displayed discordant patterns, including those with relatively high coverage but persistently elevated ASDRs, and those with low coverage but comparatively low ASDRs. The former pattern may reflect residual confounding by other WASH dimensions, health‑care access, and surveillance intensity, whereas the latter may arise from data limitations, context-specific protective factors, or recent rapid improvements not fully captured in the underlying survey data.

## Discussion

Using the most recent Global Burden of Disease (GBD) data, we analyzed mortality and age-standardized disability-adjusted life years (DALYs), quantified 31-year epidemiologic trends via the estimated annual percentage change (EAPC), and examined the burden of diarrhea, temporal trends, and spatial heterogeneity associated with the lack of access to handwashing facilities. Our analysis revealed that, from 1990 to 2021, the global burden of diarrhea declined significantly; however, both the absolute burden and the rate of decline varied considerably across different regions. Western and Eastern sub-Saharan Africa continue to bear the highest per-capita mortality and DALY burden, with DALY rates still exceeding 600 per 100,000 in 2021. In contrast, South Asia accounts for the largest absolute number of cases, highlighting the urgent need for large-scale infrastructure investment tailored to the region’s population size. While the burden of diarrhea has greatly decreased in high-income settings, a recent increase in deaths and YLL rates in North America underscores the need to monitor vulnerable subgroups. We also observed a rapid decline in YLLs but only modest reductions in YLDs. While diarrhea-related mortality attributable to the absence of handwashing facilities remains concentrated in boys under the age of five, the burden of disability is increasingly shifting towards women of reproductive age and adolescents. Despite projections suggesting continued declines in both incidence and mortality, the pace of improvement is likely to slow.

Although the World Health Organization (WHO) established World Hand Hygiene Day in 2009 to promote disease prevention, national-level messaging has frequently been ineffective in conveying the importance of hand hygiene, contributing to persistently low coverage of facilities and limited public knowledge in many countries [28]. To address gaps in coverage, weak surveillance, and financing shortfalls, sustained global investment in WASH infrastructure, broader dissemination of hygiene knowledge, and advancements in relevant technologies are urgently needed.

### Regional disparities persist despite global progress

Marked regional disparities persist despite global progress in health outcomes. In high-SDI (socio-demographic index) settings, increasing death counts alongside decreasing age-standardized death rates (ASDRs) are likely attributed to population aging and improved case ascertainment. In contrast, low- and lower-middle-SDI regions show declines in burden, but these reductions start from much higher baseline levels, with persistently elevated deaths, disability-adjusted life years (DALYs), years lived with disability (YLDs), and years of life lost (YLLs)—particularly in sub-Saharan Africa and South Asia. In these regions, multiple factors such as low socioeconomic development, inadequate public health infrastructure, and limited medical resources continue to impede rapid improvements. Given the very small absolute numbers of deaths in high‑SDI regions, the corresponding estimates should be interpreted with caution, and the uncertainty intervals reported in Table 1 and Fig. 1 highlight this limitation.

Broadly, low-income regions (e.g., sub-Saharan Africa, South Asia) exhibit a high but declining burden, upper-middle-income regions (e.g., northern Latin America, Southeast Asia) show stable to slowly decreasing burden, and high-income regions (e.g., Western Europe, North America) maintain a low, stable burden. Addressing infrastructure and hygiene-education deficits in low-income settings, alongside closing urbanization-related sanitation gaps in lower-middle-income settings, remains central to achieving sustained reductions in the diarrheal burden.

Age-specific patterns provide further clarity on risk. Mortality is highest among infants (< 1 year) and young children (< 5 years), highlighting their heightened vulnerability when handwashing facilities are absent. Mortality rates remain elevated in adults aged ≥ 70 years, and as populations age, both the absolute number of deaths and the proportion of deaths in this group increase. Several mechanisms contribute to these trends: immunologic immaturity renders children, particularly infants, more susceptible to bacterial and viral enteropathogens; climatic factors (e.g., extreme heat and precipitation) exacerbate the risks of bacterial and viral diarrhea, disproportionately affecting young children [29]; and a lack of access to clean water and handwashing facilities in households is strongly associated with increased diarrheal incidence in children under five, the likelihood of reporting diarrhea among the children decreases with the age of children [30]. Older adults face elevated risks due to comorbidities and frailty.

Sex-specific analyses reveal a significantly higher incidence and mortality from diarrheal disease attributable to lack of handwashing facilities in men compared to women, although recent trends indicate a narrowing of male–female differences in mortality and Disability-Adjusted Life Years (DALYs). Notably, however, women exhibit higher Years Lived with Disability (YLDs). These gender disparities may be attributed to gendered roles within households and society. On one hand, men tend to bear a slightly greater burden of diarrheal diseases, potentially linked to hygiene practices during outdoor activities and increased opportunities for pathogen exposure [31]. On the other hand, in many low- and middle-income countries, women often bear the primary responsibility for water collection and maintaining household sanitation (e.g., latrine cleaning), tasks that are time-consuming, restrict other opportunities, and pose both health and safety risks. Pregnancy and lactation further amplify women's dependence on reliable hygiene resources [32]. The elevated YLDs among women of reproductive age likely reflect the cumulative impact of recurrent and sometimes persistent diarrheal episodes during years when women often shoulder multiple roles, including income generation, childcare, food preparation, and water collection. Diarrheal illness in this group may lead to reduced work productivity, lost wages, school absenteeism, and interruptions in caregiving responsibilities, as well as increased risk of undernutrition and anemia. These consequences can in turn affect pregnancy outcomes and child health, underscoring the broader intergenerational implications of inadequate handwashing facilities for women. Beyond diarrheal disease, numerous qualitative and quantitative studies have documented that women and girls face disproportionate WASH‑related risks, including musculoskeletal strain and injury from water carrying, anxiety and harassment associated with using shared or unsafe sanitation facilities, and challenges managing menstruation safely and with dignity. Although these outcomes were not captured in our burden estimates, they further underscore the need for gender‑sensitive WASH and handwashing interventions. A study by Lee (2017) highlights a critical situation in certain regions of India, where surface water is scarce, rainfall is minimal, and droughts occur frequently. As a result, women and girls often have to walk 3–5 km each day to collect water for their families. Additionally, due to unsanitary conditions, women are more vulnerable to health issues and may face risks of violence when engaging in open defecation [33].

As the implementation literature on WASH has shown, handwashing behaviour is often constrained more by structural barriers than by individual knowledge or willingness. In many settings, unreliable or distant water supply, competition between drinking, cooking, and hygiene uses of water, lack of convenient space for handwashing stations, crowding, and poverty‑related opportunity costs (e.g., time spent collecting water) make it difficult to practice handwashing consistently, even when people understand its benefits. Social norms and intra‑household power dynamics may further limit women's ability to allocate water and soap for hygiene. Several large‑scale handwashing promotion programmes that relied primarily on mass media campaigns or didactic health education have demonstrated modest or short‑lived effects when they did not adequately address these structural and social barriers.

Our findings demonstrate a strong association between the Sociodemographic Index (SDI) and the diarrheal burden attributable to the lack of handwashing facilities. In particular, regions with high and high-middle SDI have been successful in reducing incidence and mortality rates through enhanced public health infrastructure, improved clinical care, and robust health education. In contrast, coverage of handwashing facilities remains alarmingly low in many areas, particularly sub-Saharan Africa, where it directly undermines diarrheal disease control efforts.

Projections suggest that the Age-Standardized Death Rate (ASDR), DALY rate, and Years of Life Lost (YLL) attributable to the absence of handwashing facilities will continue to decline, albeit at a decelerating rate. To sustain progress and further improve global health, several priorities emerge: (1) expand the coverage and consistent use of handwashing facilities—bolstered by workplace cues (e.g., reminders, posters), visible role-modeling by household heads/managers, and the provision of necessary supplies [34, 35]—while scaling up health education campaigns to raise public awareness about basic hygiene; special attention should be given to low-SDI regions, integrating infrastructure, education, and emergency preparedness and response for outbreaks of diarrheal disease, with a focus on infant and young-child health services to reduce early mortality and promote health equity, including context‑specific nudges, participatory approaches that align with local routines, and the provision of conveniently located, durable handwashing stations in homes, schools, and health‑care facilities; (2) strengthen international collaboration to share successful strategies and technologies, providing targeted support to low- and lower-middle-SDI regions to enhance public health systems and clinical capacity; (3) prioritize the management of nonfatal diseases by reducing disability through health education, the promotion of healthy behaviors, and improvements in the quality of care; and (4) establish robust, routine global health monitoring and evaluation systems to track trends and policy impacts, facilitating timely interventions to further reduce disability. High-income countries should continue to optimize their established public health systems to ensure sustained reductions in disease burden.

This study examines the global diarrheal burden attributable to lack of access to handwashing facilities from 1990 to 2021. The findings indicate that infants (< 1 year) and older adults (≥ 70 years) remain disproportionately affected, with low-SDI regions continuing to account for a disproportionately large share of the burden. Additionally, the control of diarrhea remains a major challenge in Southern and Central sub-Saharan Africa.

Several limitations merit consideration: (1) Although the GBD database covers many countries and territories, data completeness and accuracy vary by location. The absence of urban–rural disaggregation may underestimate localized high-risk areas. (2) Case definitions in the GBD database may differ from those used in other international sources. Moreover, the definition of "lack of handwashing facilities" considers only fixed installations and excludes behaviors involving flowing water and soap, which may skew the burden estimates. (3) Because our analyses rely on the GBD comparative risk assessment framework, which assigns diarrheal burden to individual WASH risk factors for diarrheal transmission according to hierarchical rules, some deaths that are jointly influenced by multiple WASH dimensions may not be fully captured under 'lack of handwashing facilities'. As a result, our estimates should be interpreted as conservative, risk‑factor‑specific burdens. (4) Our use of ARIMA models for forecasting does not incorporate potential future shocks, such as major policy changes, pandemics, or climate‑related disasters; projected trends should therefore be interpreted as illustrative.

## Conclusion

Drawing on GBD 1990–2021 data, global deaths, DALYs, and YLLs from diarrhea attributable to lack of handwashing facilities have declined by 67%, 76%, and 78%, respectively. Despite these substantial reductions, significant inequities persist: in 2021, the DALY rate in West Africa remained 52 times higher than in Western Europe. While YLLs decreased by an average of 4.8% (4.52 to 5.08) per year, YLDs declined by only 1.26% (1.14 to 1.38), resulting in an increase in the disability share to over 10%. This shift in burden is most evident among women of reproductive age and adolescents. Mortality risk remains highest among boys under five, while the absolute number of deaths is rising among adults aged 70 years and older. Additionally, female YLDs consistently exceed those of males. At the country level, trajectories are categorized into three distinct patterns: rapid convergence, high-baseline/slow decline, and low-baseline/volatile. Forecasts suggest continued reductions in diarrhea burden, albeit at a decelerating rate, through 2035.

In sum, despite substantial global progress, pronounced gradients by region, age, sex, and socioeconomic development endure. Further gains will require sustained expansion of WASH infrastructure, deeper behavior-change interventions, strengthened surveillance, and robust health financing to narrow disparities and advance toward zero preventable diarrheal deaths and universal health coverage (UHC).

## Supplementary Information

Supplementary file 1.

## Data Availability

The data used in this study are available free of charge online at https://vizhub.healthdata.org/gbd-results on request. The datasets used and/or analysed during the current study available from the corresponding author on reasonable request.
