# Supplementary material for: Thirty-one-year trends in diarrheal mortality and disability-adjusted life years attributable to lack of handwashing facilities
Source: Trop Med Health. 2026 Jan 17;54:21. doi: 10.1186/s41182-026-00903-z (PMC12829192; doi:10.1186/s41182-026-00903-z)
Supplement: Supplementary file 1 [file 41182_2026_903_MOESM1_ESM.docx]

Thirty-One-Year Trends in Diarrheal Mortality and Disability-Adjusted Life Years Attributable to Lack of Handwashing Facilities

Supplementary Material

[Table S1. The death cases and ASDR of no access to handwashing facility in 1990 and 2021, with Temporal Trends from 1990 to 2021 in 204 countries or territories. 1](#_Toc209651915)

[Table S2. The DALYs and age-standardized DALYs Rate of no access to handwashing in 1990 and 2021, with Temporal Trends from 1990 to 2021 in 204 countries or territories. 7](#_Toc209651916)

[Table S3. The YLLs and age-standardized YLLs Rate of no access to handwashing facility in 1990 and 2021, with Temporal Trends from 1990 to 2021 in 204 countries or territories. 13](#_Toc209651917)

[Table S4. The YLDs and age-standardized YLDs Rate ofno access to handwashing facility in 1990 and 2021, with Temporal Trends from 1990 to 2021 in 204 countries or territories. 19](#_Toc209651918)

| Table S1. The death cases and ASDR of no access to handwashing facility in 1990 and 2021, with Temporal Trends from 1990 to 2021 in 204 countries or territories. | | | | | |
| --- | --- | --- | --- | --- | --- |
| **location** | **Num_1990** | **ASDR_1990** | **Num_2021** | **ASDR_2021** | **EAPC_ASDR** |
| Afghanistan | 162,801 (21,950 - 305,666) | 870.26 (117.13 - 1661.61) | 81,595 (12,219 - 167,178) | 148.89 (22.66 - 302.04) | -5.98 (-6.70 to -5.25) |
| Albania | 594 (58 - 1,384) | 15.36 (1.51 - 35.76) | 28 (3 - 65) | 1.72 (0.19 - 4.12) | -7.69 (-8.30 to -7.08) |
| Algeria | 12,156 (1,523 - 28,244) | 35.27 (4.46 - 80.57) | 2,833 (344 - 6,271) | 6.58 (0.80 - 14.54) | -5.01 (-5.13 to -4.89) |
| American Samoa | 16 (2 - 36) | 42.33 (4.24 - 94.44) | 14 (1 - 31) | 32.15 (3.31 - 74.74) | -0.68 (-1.15 to -0.22) |
| Andorra | 0 (0 - 1) | 1.10 (0.12 - 2.71) | 1 (0 - 1) | 0.96 (0.09 - 2.37) | -0.12 (-0.56 - 0.32) |
| Angola | 615,934 (99,262 - 1,141,017) | 3826.79 (595.85 - 7075.55) | 137,651 (19,124 - 265,095) | 438.01 (62.03 - 841.01) | -6.91 (-7.41 to -6.41) |
| Antigua and Barbuda | 17 (2 - 36) | 29.63 (3.54 - 61.82) | 7 (1 - 16) | 9.50 (1.11 - 20.58) | -3.70 (-4.22 to -3.18) |
| Argentina | 5,565 (573 - 12,619) | 16.69 (1.72 - 37.76) | 467 (50 - 1,103) | 1.13 (0.12 - 2.68) | -7.26 (-7.69 to -6.82) |
| Armenia | 2,919 (358 - 6,234) | 79.26 (9.71 - 169.32) | 27 (3 - 63) | 1.27 (0.13 - 2.93) | -14.84 (-15.51 to -14.17) |
| Australia | 114 (12 - 282) | 0.71 (0.07 - 1.78) | 52 (5 - 132) | 0.16 (0.01 - 0.40) | -4.58 (-5.20 to -3.96) |
| Austria | 167 (19 - 394) | 2.70 (0.30 - 6.37) | 86 (9 - 197) | 0.94 (0.09 - 2.22) | -3.66 (-4.57 to -2.73) |
| Azerbaijan | 9,527 (1,096 - 21,906) | 108.99 (12.53 - 250.21) | 677 (70 - 1,679) | 9.57 (0.99 - 23.62) | -8.82 (-9.38 to -8.25) |
| Bahamas | 46 (6 - 103) | 18.76 (2.50 - 41.90) | 16 (2 - 36) | 4.94 (0.54 - 11.10) | -4.42 (-4.93 to -3.90) |
| Bahrain | 64 (8 - 143) | 12.02 (1.47 - 26.25) | 29 (3 - 72) | 2.77 (0.27 - 7.00) | -4.63 (-4.81 to -4.45) |
| Bangladesh | 1,494,677 (233,205 - 2,898,485) | 1121.47 (171.00 - 2090.65) | 195,417 (27,396 - 451,494) | 139.51 (19.17 - 323.85) | -6.61 (-6.76 to -6.45) |
| Barbados | 32 (4 - 68) | 13.57 (1.56 - 28.93) | 19 (2 - 44) | 7.54 (0.86 - 17.50) | -1.80 (-2.17 to -1.43) |
| Belarus | 350 (40 - 797) | 4.39 (0.50 - 9.98) | 51 (5 - 132) | 0.73 (0.08 - 1.86) | -7.10 (-7.73 to -6.47) |
| Belgium | 168 (19 - 373) | 1.88 (0.21 - 4.18) | 334 (37 - 782) | 1.82 (0.19 - 4.30) | 0.36 (-0.29 - 1.01) |
| Belize | 542 (75 - 1,017) | 198.82 (27.53 - 371.36) | 118 (14 - 232) | 30.34 (3.67 - 60.21) | -5.74 (-6.26 to -5.22) |
| Benin | 165,631 (27,220 - 298,888) | 2388.71 (376.52 - 4286.53) | 82,516 (11,429 - 172,834) | 582.16 (78.69 - 1137.04) | -4.45 (-4.60 to -4.31) |
| Bermuda | 5 (1 - 11) | 10.41 (1.25 - 21.78) | 3 (0 - 7) | 6.19 (0.67 - 14.28) | -1.69 (-2.01 to -1.36) |
| Bhutan | 13,256 (1,594 - 30,358) | 1856.43 (219.27 - 4302.12) | 391 (43 - 1,049) | 63.66 (7.06 - 170.70) | -11.35 (-11.67 to -11.03) |
| Bolivia (Plurinational State of) | 32,786 (4,061 - 69,250) | 344.46 (42.44 - 720.68) | 3,175 (362 - 7,197) | 28.15 (3.20 - 64.18) | -8.25 (-8.39 to -8.11) |
| Bosnia and Herzegovina | 117 (13 - 258) | 3.36 (0.36 - 7.43) | 20 (2 - 54) | 0.83 (0.09 - 2.05) | -4.30 (-4.75 to -3.85) |
| Botswana | 21,695 (3,160 - 38,719) | 1422.63 (196.64 - 2685.85) | 9,480 (1,301 - 18,150) | 429.19 (58.25 - 823.89) | -3.73 (-4.04 to -3.43) |
| Brazil | 478,968 (65,043 - 916,557) | 316.41 (42.97 - 604.83) | 16,451 (1,643 - 37,024) | 8.18 (0.81 - 18.44) | -11.60 (-11.83 to -11.37) |
| Brunei Darussalam | 2 (0 - 5) | 0.91 (0.10 - 2.28) | 1 (0 - 4) | 0.43 (0.04 - 1.10) | -1.87 (-2.05 to -1.68) |
| Bulgaria | 96 (10 - 225) | 1.77 (0.19 - 4.15) | 56 (5 - 134) | 1.10 (0.10 - 2.63) | -1.38 (-2.56 to -0.19) |
| Burkina Faso | 455,010 (62,826 - 816,737) | 2985.82 (429.71 - 5426.19) | 250,972 (41,439 - 456,040) | 837.07 (133.37 - 1466.38) | -4.10 (-4.27 to -3.93) |
| Burundi | 153,001 (21,147 - 288,374) | 1965.92 (283.16 - 3666.29) | 97,494 (13,306 - 200,920) | 767.98 (100.48 - 1497.99) | -2.85 (-3.07 to -2.63) |
| Cabo Verde | 111,749 (14,793 - 231,405) | 765.87 (101.27 - 1561.12) | 6,619 (765 - 14,473) | 44.91 (5.18 - 98.92) | -9.83 (-10.28 to -9.39) |
| Cambodia | 237,467 (35,688 - 465,876) | 1537.60 (237.46 - 2972.38) | 154,122 (19,516 - 340,046) | 437.65 (57.76 - 883.97) | -3.76 (-4.27 to -3.25) |
| Cameroon | 101 (11 - 251) | 0.40 (0.04 - 1.00) | 132 (14 - 320) | 0.25 (0.03 - 0.59) | -0.62 (-1.65 - 0.42) |
| Canada | 5,285 (773 - 9,434) | 1050.94 (154.33 - 1883.39) | 644 (103 - 1,207) | 133.49 (21.26 - 251.65) | -7.08 (-7.68 to -6.47) |
| Central African Republic | 115,183 (19,125 - 209,780) | 2704.96 (432.77 - 4958.00) | 88,590 (12,076 - 178,144) | 1427.21 (191.64 - 2882.36) | -1.75 (-1.90 to -1.60) |
| Chad | 471,873 (72,832 - 882,152) | 4612.11 (700.17 - 8571.84) | 632,314 (92,632 - 1,264,787) | 2239.97 (315.74 - 4315.41) | -2.24 (-2.41 to -2.07) |
| Chile | 825 (91 - 1,890) | 6.31 (0.70 - 14.44) | 130 (14 - 316) | 0.71 (0.08 - 1.74) | -6.41 (-6.56 to -6.25) |
| China | 934,019 (108,347 - 1,886,528) | 85.51 (9.91 - 173.13) | 10,318 (1,108 - 22,067) | 1.02 (0.11 - 2.11) | -14.76 (-15.28 to -14.24) |
| Colombia | 31,845 (3,963 - 67,265) | 80.27 (10.02 - 169.40) | 2,668 (313 - 6,315) | 6.31 (0.74 - 14.93) | -8.78 (-9.30 to -8.26) |
| Comoros | 7,702 (1,120 - 14,800) | 1165.24 (168.98 - 2199.25) | 2,386 (315 - 4,658) | 344.82 (44.28 - 663.61) | -3.89 (-4.17 to -3.62) |
| Congo | 44,784 (5,899 - 88,264) | 1393.04 (193.56 - 2733.21) | 13,069 (1,573 - 28,776) | 278.82 (33.05 - 608.94) | -4.98 (-5.42 to -4.54) |
| Cook Islands | 2 (0 - 5) | 13.40 (1.57 - 30.85) | 2 (0 - 4) | 11.28 (1.18 - 25.63) | -0.46 (-0.88 to -0.04) |
| Costa Rica | 784 (88 - 1,787) | 24.30 (2.75 - 55.72) | 216 (23 - 508) | 4.90 (0.53 - 11.59) | -4.55 (-5.06 to -4.03) |
| Côte d'Ivoire | 24 (2 - 57) | 0.74 (0.08 - 1.77) | 27 (3 - 65) | 0.68 (0.08 - 1.67) | 0.87 (-0.77 - 2.54) |
| Croatia | 2,491 (324 - 5,273) | 26.12 (3.39 - 55.37) | 824 (93 - 1,924) | 7.65 (0.85 - 17.83) | -4.21 (-4.70 to -3.72) |
| Cuba | 32 (3 - 73) | 4.87 (0.52 - 11.22) | 28 (3 - 65) | 2.08 (0.25 - 4.90) | -2.26 (-2.46 to -2.05) |
| Cyprus | 105 (12 - 254) | 1.46 (0.17 - 3.55) | 163 (18 - 391) | 1.41 (0.15 - 3.43) | 1.29 (-0.64 - 3.27) |
| Czechia | 263,222 (38,469 - 477,074) | 1443.40 (210.17 - 2663.98) | 125,086 (16,265 - 246,649) | 402.70 (52.48 - 766.07) | -3.61 (-3.93 to -3.30) |
| Democratic People's Republic of Korea | 80 (7 - 198) | 1.68 (0.14 - 4.08) | 162 (15 - 402) | 2.03 (0.19 - 5.03) | 1.43 (0.88 - 1.98) |
| Democratic Republic of the Congo | 9,521 (1,513 - 17,624) | 1778.55 (278.55 - 3295.47) | 3,829 (496 - 7,461) | 347.63 (43.86 - 663.30) | -5.28 (-5.52 to -5.05) |
| Denmark | 33 (4 - 67) | 41.61 (4.60 - 84.61) | 8 (1 - 19) | 17.98 (2.08 - 42.80) | -2.57 (-3.03 to -2.10) |
| Djibouti | 40,271 (5,954 - 78,261) | 408.37 (59.91 - 790.96) | 5,704 (759 - 12,277) | 55.05 (7.32 - 118.44) | -6.23 (-6.60 to -5.85) |
| Dominica | 1,038,410 (157,965 - 1,863,657) | 1721.71 (261.44 - 3112.51) | 325,932 (46,152 - 656,384) | 375.64 (52.11 - 756.53) | -4.80 (-5.77 to -3.82) |
| Dominican Republic | 24,577 (3,267 - 51,598) | 200.31 (26.42 - 419.36) | 1,029 (123 - 2,322) | 6.12 (0.73 - 13.86) | -11.45 (-11.87 to -11.02) |
| Ecuador | 300,641 (36,946 - 639,524) | 354.08 (43.63 - 752.29) | 16,104 (1,594 - 38,208) | 13.76 (1.39 - 32.26) | -9.89 (-10.35 to -9.42) |
| Egypt | 26,322 (3,160 - 53,947) | 361.18 (42.94 - 732.60) | 885 (87 - 2,066) | 14.40 (1.41 - 33.43) | -10.47 (-11.18 to -9.75) |
| El Salvador | 22,004 (3,250 - 41,780) | 3258.37 (495.06 - 6345.27) | 1,871 (247 - 3,832) | 148.94 (18.68 - 307.97) | -11.09 (-11.60 to -10.58) |
| Equatorial Guinea | 149,744 (21,124 - 266,657) | 3365.52 (488.57 - 6336.77) | 48,972 (6,335 - 98,808) | 820.80 (101.16 - 1776.92) | -4.55 (-4.66 to -4.43) |
| Eritrea | 34 (4 - 84) | 2.78 (0.30 - 6.81) | 6 (1 - 15) | 0.60 (0.06 - 1.46) | -5.20 (-5.59 to -4.81) |
| Estonia | 22,757 (3,457 - 42,231) | 2075.05 (313.72 - 3894.25) | 6,480 (992 - 13,165) | 556.12 (83.64 - 1095.25) | -4.09 (-4.57 to -3.61) |
| Eswatini | 1,979,630 (299,172 - 3,994,569) | 2994.03 (507.88 - 6013.25) | 695,027 (115,326 - 1,199,754) | 683.97 (108.12 - 1211.91) | -5.04 (-5.20 to -4.88) |
| Ethiopia | 172 (21 - 358) | 181.61 (21.89 - 385.93) | 44 (5 - 95) | 59.21 (6.67 - 132.79) | -3.44 (-4.30 to -2.56) |
| Fiji | 840 (101 - 1,736) | 149.75 (17.63 - 315.86) | 486 (54 - 1,128) | 65.71 (7.27 - 152.26) | -2.20 (-2.54 to -1.86) |
| Finland | 67 (7 - 166) | 1.52 (0.15 - 3.79) | 51 (6 - 122) | 0.82 (0.10 - 2.01) | -1.86 (-2.59 to -1.13) |
| France | 1,222 (146 - 2,774) | 2.21 (0.26 - 4.99) | 1,448 (145 - 3,409) | 2.30 (0.23 - 5.52) | 0.53 (0.12 - 0.94) |
| Gabon | 12,421 (1,639 - 25,751) | 997.20 (141.39 - 2030.79) | 2,231 (297 - 5,000) | 138.00 (18.05 - 306.65) | -6.24 (-6.45 to -6.03) |
| Gambia | 947 (95 - 2,198) | 21.39 (2.14 - 49.40) | 24 (2 - 58) | 0.82 (0.09 - 1.97) | -11.23 (-11.94 to -10.51) |
| Georgia | 1,088 (114 - 2,617) | 1.63 (0.17 - 3.88) | 1,265 (143 - 2,906) | 0.98 (0.11 - 2.26) | -0.71 (-1.63 - 0.22) |
| Germany | 269,168 (44,297 - 507,700) | 1408.63 (241.68 - 2644.05) | 91,643 (13,744 - 168,435) | 303.72 (45.56 - 568.18) | -4.80 (-4.96 to -4.63) |
| Ghana | 142 (12 - 376) | 1.67 (0.14 - 4.41) | 91 (11 - 220) | 1.05 (0.12 - 2.65) | -0.81 (-1.33 to -0.27) |
| Greece | 0 (0 - 1) | 0.72 (0.07 - 1.76) | 0 (0 - 0) | 0.31 (0.03 - 0.84) | -2.53 (-2.67 to -2.38) |
| Greenland | 46 (6 - 92) | 44.94 (5.85 - 87.72) | 13 (2 - 29) | 14.14 (1.68 - 30.85) | -3.43 (-3.90 to -2.95) |
| Grenada | 16 (2 - 37) | 14.52 (1.64 - 33.69) | 17 (2 - 41) | 11.01 (1.37 - 25.63) | -0.87 (-1.49 to -0.25) |
| Guam | 119,212 (15,989 - 233,318) | 1072.84 (143.71 - 2080.49) | 12,614 (1,610 - 27,125) | 88.88 (11.32 - 191.33) | -7.48 (-7.81 to -7.15) |
| Guatemala | 274,911 (45,968 - 515,917) | 3175.57 (510.53 - 6048.40) | 70,835 (9,744 - 149,017) | 559.16 (75.99 - 1143.12) | -5.43 (-5.63 to -5.23) |
| Guinea | 38,882 (6,319 - 72,241) | 2797.73 (441.21 - 5255.84) | 11,002 (1,654 - 20,878) | 593.08 (82.25 - 1119.35) | -5.10 (-5.64 to -4.56) |
| Guinea-Bissau | 3,401 (440 - 6,787) | 330.34 (42.72 - 652.42) | 369 (51 - 779) | 51.93 (7.17 - 109.53) | -5.79 (-6.03 to -5.54) |
| Guyana | 240,629 (34,412 - 428,903) | 2285.17 (322.75 - 4075.32) | 92,194 (13,959 - 172,249) | 616.73 (92.00 - 1149.85) | -3.94 (-4.42 to -3.46) |
| Haiti | 25,565 (3,037 - 51,189) | 379.34 (44.97 - 770.02) | 3,254 (337 - 8,002) | 36.72 (3.79 - 92.40) | -7.43 (-7.67 to -7.19) |
| Honduras | 128 (14 - 306) | 1.86 (0.20 - 4.46) | 146 (13 - 374) | 1.64 (0.15 - 4.23) | 0.76 (-0.99 - 2.53) |
| Hungary | 3 (0 - 9) | 1.40 (0.14 - 3.58) | 5 (0 - 11) | 1.31 (0.12 - 3.26) | 0.38 (-0.15 - 0.90) |
| Iceland | 16,632,207 (2,507,161 - 30,196,315) | 2066.59 (308.65 - 3858.03) | 2,608,970 (311,636 - 5,530,816) | 224.44 (26.74 - 477.96) | -7.00 (-7.28 to -6.72) |
| India | 1,659,094 (201,723 - 3,537,707) | 934.20 (114.66 - 2055.65) | 140,761 (14,440 - 330,702) | 65.80 (6.46 - 154.02) | -7.92 (-8.14 to -7.69) |
| Indonesia | 41,237 (4,041 - 95,307) | 55.17 (5.52 - 125.53) | 2,274 (230 - 5,400) | 3.07 (0.31 - 7.35) | -8.71 (-8.95 to -8.48) |
| Iran (Islamic Republic of) | 18,875 (1,913 - 44,243) | 62.59 (6.38 - 146.16) | 2,343 (226 - 5,511) | 5.81 (0.56 - 14.03) | -7.92 (-8.16 to -7.69) |
| Iraq | 54 (6 - 140) | 1.57 (0.17 - 4.06) | 57 (6 - 134) | 1.22 (0.13 - 2.89) | 1.01 (0.09 - 1.95) |
| Ireland | 106 (13 - 261) | 2.11 (0.25 - 5.22) | 205 (22 - 473) | 1.80 (0.20 - 4.22) | 0.27 (-0.46 - 1.01) |
| Israel | 473 (47 - 1,149) | 1.06 (0.11 - 2.59) | 724 (93 - 1,650) | 0.78 (0.10 - 1.77) | -0.46 (-0.83 to -0.08) |
| Italy | 2,693 (352 - 5,390) | 103.62 (13.52 - 206.81) | 342 (38 - 706) | 13.85 (1.53 - 28.45) | -5.49 (-6.58 to -4.39) |
| Jamaica | 1,280 (146 - 3,167) | 1.23 (0.14 - 3.04) | 1,335 (128 - 3,255) | 1.11 (0.11 - 2.78) | 0.19 (-0.18 - 0.56) |
| Japan | 913 (106 - 1,996) | 16.35 (1.88 - 36.00) | 399 (45 - 938) | 3.59 (0.40 - 8.41) | -4.58 (-4.83 to -4.33) |
| Jordan | 4,198 (425 - 9,574) | 23.21 (2.35 - 52.95) | 74 (8 - 177) | 0.38 (0.04 - 0.92) | -14.90 (-15.68 to -14.11) |
| Kazakhstan | 570,801 (83,415 - 1,077,888) | 1787.44 (268.77 - 3350.17) | 240,367 (36,482 - 426,970) | 535.28 (79.39 - 952.71) | -3.45 (-3.78 to -3.12) |
| Kenya | 750 (102 - 1,537) | 1004.66 (135.99 - 2076.53) | 289 (35 - 629) | 329.43 (39.48 - 713.74) | -3.31 (-3.55 to -3.07) |
| Kiribati | 85 (10 - 192) | 4.82 (0.54 - 10.80) | 53 (6 - 132) | 1.47 (0.17 - 3.61) | -2.23 (-2.67 to -1.79) |
| Kuwait | 4,340 (507 - 10,286) | 69.70 (8.13 - 165.11) | 273 (32 - 664) | 3.60 (0.42 - 8.73) | -10.16 (-10.91 to -9.41) |
| Kyrgyzstan | 114,852 (16,026 - 229,531) | 1791.92 (253.37 - 3740.37) | 8,650 (1,078 - 18,335) | 119.99 (15.02 - 254.64) | -8.76 (-9.01 to -8.51) |
| Lao People's Democratic Republic | 57 (6 - 132) | 2.77 (0.27 - 6.35) | 6 (1 - 16) | 0.45 (0.05 - 1.11) | -6.24 (-6.60 to -5.88) |
| Latvia | 994 (129 - 2,259) | 27.11 (3.52 - 61.32) | 302 (32 - 755) | 6.25 (0.65 - 15.43) | -4.75 (-4.92 to -4.58) |
| Lebanon | 53,464 (8,866 - 94,952) | 2883.27 (466.44 - 5149.57) | 27,106 (4,380 - 48,521) | 1526.00 (237.86 - 2841.37) | -1.86 (-2.12 to -1.61) |
| Lesotho | 133,849 (20,565 - 231,098) | 3164.34 (495.37 - 5428.92) | 38,306 (5,295 - 78,834) | 660.42 (93.13 - 1260.83) | -5.44 (-5.73 to -5.15) |
| Liberia | 3,914 (474 - 8,660) | 67.72 (8.19 - 148.08) | 340 (41 - 794) | 6.40 (0.77 - 15.21) | -6.76 (-7.74 to -5.77) |
| Libya | 118 (13 - 270) | 3.94 (0.44 - 8.99) | 16 (2 - 42) | 0.72 (0.08 - 1.92) | -6.43 (-7.05 to -5.80) |
| Lithuania | 5 (1 - 12) | 1.51 (0.17 - 3.70) | 9 (1 - 21) | 1.13 (0.11 - 2.73) | -0.60 (-1.24 - 0.05) |
| Luxembourg | 487,612 (76,544 - 845,838) | 2625.17 (415.45 - 4575.07) | 342,746 (45,646 - 660,735) | 1066.80 (142.22 - 1988.12) | -2.44 (-2.68 to -2.20) |
| Madagascar | 525,676 (70,905 - 953,335) | 3343.99 (483.04 - 6089.94) | 128,728 (18,762 - 230,719) | 752.21 (106.81 - 1344.12) | -4.89 (-4.98 to -4.80) |
| Malawi | 2,566 (276 - 6,334) | 14.67 (1.53 - 36.28) | 1,395 (148 - 3,580) | 4.94 (0.52 - 12.60) | -3.10 (-3.78 to -2.41) |
| Malaysia | 1,092 (132 - 2,631) | 314.27 (37.15 - 757.70) | 28 (3 - 67) | 7.69 (0.80 - 18.21) | -11.48 (-12.38 to -10.57) |
| Maldives | 366,512 (61,522 - 696,475) | 2851.70 (455.98 - 5354.53) | 168,999 (25,834 - 310,173) | 659.43 (91.37 - 1290.86) | -4.61 (-4.84 to -4.37) |
| Mali | 6 (1 - 14) | 1.76 (0.17 - 4.23) | 5 (1 - 13) | 1.33 (0.16 - 3.36) | -0.42 (-0.85 - 0.02) |
| Malta | 77 (10 - 159) | 207.27 (25.84 - 442.42) | 31 (4 - 67) | 78.78 (10.15 - 174.18) | -2.80 (-3.32 to -2.29) |
| Marshall Islands | 42,865 (7,061 - 82,379) | 1497.88 (247.12 - 2850.42) | 14,108 (1,957 - 28,376) | 309.82 (43.15 - 591.02) | -5.11 (-5.48 to -4.74) |
| Mauritania | 112 (12 - 271) | 11.15 (1.18 - 26.85) | 27 (3 - 62) | 2.48 (0.25 - 5.62) | -3.28 (-3.72 to -2.84) |
| Mauritius | 207,976 (28,170 - 450,188) | 197.25 (26.56 - 428.48) | 9,880 (1,059 - 22,292) | 8.88 (0.95 - 19.94) | -9.42 (-10.03 to -8.80) |
| Mexico | 1,094 (127 - 2,329) | 27.08 (3.14 - 57.58) | 48 (5 - 106) | 2.22 (0.24 - 4.95) | -8.52 (-8.89 to -8.16) |
| Micronesia (Federated States of) | 0 (0 - 1) | 1.03 (0.10 - 2.57) | 0 (0 - 1) | 0.95 (0.09 - 2.31) | 0.09 (-0.33 - 0.51) |
| Monaco | 2,992 (392 - 6,800) | 88.44 (11.60 - 199.76) | 260 (23 - 719) | 6.95 (0.61 - 19.20) | -8.22 (-8.51 to -7.94) |
| Mongolia | 3 (0 - 7) | 0.56 (0.06 - 1.28) | 1 (0 - 2) | 0.17 (0.02 - 0.40) | -3.59 (-4.05 to -3.13) |
| Montenegro | 173,148 (24,067 - 358,364) | 480.39 (66.71 - 995.30) | 5,900 (643 - 14,512) | 18.38 (2.00 - 45.40) | -9.99 (-10.25 to -9.73) |
| Morocco | 479,804 (75,893 - 892,395) | 2377.23 (377.07 - 4542.22) | 172,100 (25,615 - 344,868) | 543.03 (71.55 - 1103.99) | -4.60 (-4.78 to -4.42) |
| Mozambique | 410,720 (51,307 - 961,874) | 893.73 (108.76 - 2071.40) | 25,345 (2,600 - 54,057) | 50.20 (5.14 - 106.92) | -9.40 (-9.60 to -9.20) |
| Myanmar | 26,144 (3,677 - 50,141) | 1577.60 (208.70 - 3082.71) | 10,577 (1,467 - 21,944) | 448.96 (61.78 - 908.00) | -3.88 (-4.12 to -3.64) |
| Namibia | 10 (1 - 20) | 99.75 (11.41 - 207.24) | 4 (0 - 10) | 48.47 (5.66 - 110.58) | -2.16 (-2.52 to -1.79) |
| Nauru | 370,529 (58,089 - 665,237) | 1424.04 (215.62 - 2650.38) | 25,879 (3,656 - 53,374) | 98.89 (13.82 - 208.27) | -8.47 (-8.71 to -8.24) |
| Nepal | 206 (24 - 499) | 1.64 (0.19 - 3.96) | 423 (49 - 1,012) | 2.61 (0.31 - 6.36) | 2.01 (1.34 - 2.68) |
| Netherlands | 41 (4 - 96) | 1.25 (0.13 - 2.95) | 30 (3 - 73) | 0.51 (0.05 - 1.23) | -1.78 (-2.43 to -1.12) |
| New Zealand | 28,981 (3,772 - 58,473) | 465.42 (60.27 - 945.00) | 1,224 (144 - 2,838) | 19.97 (2.34 - 46.05) | -9.94 (-10.33 to -9.56) |
| Nicaragua | 701,226 (110,864 - 1,244,902) | 5086.07 (802.16 - 9028.20) | 361,657 (51,720 - 699,651) | 1137.30 (158.53 - 2179.24) | -5.15 (-5.48 to -4.81) |
| Niger | 4,800,679 (731,783 - 8,576,773) | 3316.62 (513.99 - 5937.12) | 2,904,526 (404,014 - 5,290,919) | 915.28 (128.76 - 1635.41) | -4.02 (-4.41 to -3.62) |
| Nigeria | 1 (0 - 3) | 57.61 (6.74 - 123.84) | 1 (0 - 1) | 39.16 (4.84 - 93.56) | -2.33 (-2.97 to -1.68) |
| Niue | 1,022 (102 - 2,311) | 4.48 (0.44 - 10.23) | 1,182 (141 - 2,867) | 5.11 (0.61 - 12.37) | 1.53 (1.15 - 1.91) |
| North Macedonia | 425 (43 - 938) | 25.77 (2.63 - 56.89) | 24 (2 - 60) | 1.77 (0.16 - 4.54) | -7.45 (-8.39 to -6.51) |
| Northern Mariana Islands | 5 (1 - 12) | 19.25 (2.23 - 46.59) | 8 (1 - 18) | 19.53 (1.99 - 44.43) | 0.61 (0.08 - 1.15) |
| Norway | 52 (6 - 134) | 1.19 (0.13 - 3.16) | 125 (15 - 299) | 1.34 (0.15 - 3.21) | 0.80 (0.42 - 1.18) |
| Oman | 1,218 (151 - 2,844) | 43.15 (5.28 - 96.63) | 136 (16 - 335) | 3.75 (0.44 - 9.34) | -7.53 (-8.27 to -6.77) |
| Pakistan | 1,805,699 (251,216 - 3,451,302) | 1201.77 (163.43 - 2331.96) | 342,390 (41,122 - 680,920) | 153.50 (17.77 - 311.77) | -6.05 (-6.38 to -5.72) |
| Palau | 10 (1 - 23) | 84.53 (9.47 - 202.70) | 6 (1 - 15) | 41.97 (4.75 - 102.23) | -1.92 (-2.42 to -1.42) |
| Palestine | 2,519 (309 - 5,386) | 68.21 (8.22 - 143.24) | 226 (26 - 516) | 4.15 (0.46 - 9.40) | -7.81 (-8.17 to -7.44) |
| Panama | 1,988 (209 - 4,473) | 77.08 (8.10 - 172.56) | 478 (55 - 1,083) | 12.31 (1.41 - 27.75) | -5.04 (-5.42 to -4.67) |
| Papua New Guinea | 46,301 (6,073 - 85,047) | 966.69 (123.30 - 1789.83) | 45,615 (6,412 - 93,608) | 407.07 (54.95 - 800.69) | -2.33 (-2.53 to -2.14) |
| Paraguay | 11,311 (1,481 - 22,801) | 208.15 (27.10 - 416.59) | 616 (71 - 1,465) | 9.69 (1.11 - 23.21) | -10.52 (-10.92 to -10.11) |
| Peru | 55,629 (6,446 - 112,311) | 201.55 (23.77 - 409.51) | 3,612 (390 - 8,451) | 10.57 (1.14 - 24.64) | -9.82 (-10.25 to -9.39) |
| Philippines | 197,579 (26,293 - 423,588) | 240.90 (32.60 - 510.47) | 28,699 (3,333 - 64,496) | 27.62 (3.15 - 60.82) | -6.47 (-6.62 to -6.32) |
| Poland | 283 (31 - 653) | 0.99 (0.11 - 2.28) | 316 (33 - 764) | 0.74 (0.07 - 1.80) | 0.70 (-1.25 - 2.69) |
| Portugal | 192 (21 - 433) | 2.58 (0.28 - 5.80) | 161 (18 - 393) | 1.08 (0.13 - 2.68) | -1.89 (-2.20 to -1.58) |
| Puerto Rico | 390 (46 - 834) | 11.36 (1.32 - 24.37) | 203 (22 - 443) | 6.29 (0.68 - 13.78) | -1.98 (-2.27 to -1.69) |
| Qatar | 22 (2 - 51) | 5.13 (0.52 - 11.87) | 27 (3 - 62) | 1.51 (0.14 - 3.37) | -3.86 (-3.99 to -3.73) |
| Republic of Korea | 1,716 (189 - 3,825) | 10.82 (1.19 - 24.16) | 237 (23 - 544) | 1.44 (0.13 - 3.27) | -6.78 (-7.94 to -5.60) |
| Republic of Moldova | 7,199 (896 - 16,394) | 6.34 (0.79 - 14.44) | 856 (104 - 2,065) | 0.75 (0.09 - 1.82) | -7.35 (-7.78 to -6.92) |
| Romania | 204,198 (30,755 - 369,278) | 1841.58 (281.00 - 3383.09) | 54,502 (8,669 - 104,516) | 409.25 (63.47 - 789.44) | -5.69 (-6.30 to -5.08) |
| Russian Federation | 50 (6 - 102) | 117.33 (14.11 - 240.24) | 9 (1 - 20) | 20.27 (2.47 - 44.48) | -5.34 (-6.09 to -4.59) |
| Rwanda | 106 (12 - 207) | 71.64 (8.43 - 138.96) | 23 (3 - 51) | 15.71 (1.68 - 34.25) | -4.77 (-5.51 to -4.03) |
| Saint Kitts and Nevis | 156 (21 - 311) | 137.48 (18.37 - 269.85) | 20 (2 - 41) | 19.92 (2.45 - 42.59) | -5.88 (-6.61 to -5.16) |
| Saint Lucia | 40 (5 - 91) | 27.17 (3.35 - 63.52) | 22 (2 - 52) | 11.76 (1.18 - 27.25) | -2.77 (-3.12 to -2.42) |
| Saint Vincent and the Grenadines | 0 (0 - 1) | 1.13 (0.12 - 2.74) | 0 (0 - 1) | 0.96 (0.10 - 2.49) | -0.09 (-0.46 - 0.29) |
| Samoa | 12,272 (1,401 - 27,357) | 58.27 (6.69 - 130.19) | 1,066 (111 - 2,688) | 3.98 (0.40 - 9.99) | -8.48 (-8.90 to -8.06) |
| San Marino | 291,472 (45,436 - 527,352) | 2586.65 (398.05 - 4620.68) | 48,555 (6,597 - 93,784) | 320.05 (45.52 - 609.28) | -6.68 (-7.03 to -6.33) |
| Sao Tome and Principe | 166 (18 - 397) | 2.37 (0.26 - 5.68) | 36 (4 - 93) | 0.55 (0.06 - 1.31) | -4.28 (-4.56 to -4.01) |
| Saudi Arabia | 10 (1 - 24) | 13.63 (1.32 - 33.23) | 5 (1 - 14) | 5.70 (0.54 - 14.46) | -2.10 (-2.44 to -1.76) |
| Senegal | 170,699 (23,689 - 318,697) | 2607.59 (362.41 - 4854.10) | 62,882 (8,730 - 123,027) | 695.79 (99.22 - 1345.13) | -4.53 (-4.99 to -4.06) |
| Serbia | 20 (2 - 47) | 0.90 (0.08 - 2.13) | 11 (1 - 27) | 0.18 (0.02 - 0.43) | -4.07 (-4.55 to -3.60) |
| Seychelles | 31 (3 - 75) | 0.75 (0.08 - 1.80) | 14 (1 - 37) | 0.39 (0.04 - 1.06) | -1.55 (-2.16 to -0.94) |
| Sierra Leone | 5 (1 - 12) | 0.38 (0.05 - 0.91) | 6 (1 - 16) | 0.30 (0.03 - 0.75) | -0.02 (-0.90 - 0.87) |
| Singapore | 1,430 (209 - 2,774) | 415.84 (61.89 - 836.29) | 629 (80 - 1,232) | 114.27 (15.15 - 234.15) | -3.80 (-3.95 to -3.65) |
| Slovakia | 382,493 (61,150 - 740,298) | 3336.31 (528.90 - 6608.93) | 346,693 (54,193 - 662,499) | 1426.94 (214.09 - 2785.04) | -2.98 (-3.12 to -2.83) |
| Slovenia | 532,121 (78,716 - 967,349) | 1221.72 (180.23 - 2220.86) | 175,693 (22,646 - 336,439) | 350.45 (45.27 - 677.07) | -3.82 (-4.15 to -3.48) |
| Solomon Islands | 553 (60 - 1,451) | 1.79 (0.19 - 4.73) | 227 (23 - 559) | 0.40 (0.04 - 1.00) | -4.07 (-4.39 to -3.74) |
| Somalia | 303,766 (43,859 - 582,544) | 3524.78 (511.26 - 6744.54) | 253,028 (35,306 - 498,688) | 2156.65 (298.66 - 4309.56) | -1.37 (-1.56 to -1.17) |
| South Africa | 636 (69 - 1,553) | 1.96 (0.21 - 4.76) | 765 (92 - 1,773) | 1.45 (0.18 - 3.33) | -0.44 (-0.75 to -0.13) |
| South Sudan | 16,367 (1,916 - 37,075) | 114.38 (13.50 - 259.96) | 1,448 (168 - 3,590) | 6.80 (0.81 - 16.65) | -8.53 (-9.11 to -7.94) |
| Spain | 254,711 (32,552 - 574,722) | 708.30 (90.99 - 1563.00) | 21,426 (2,026 - 61,076) | 42.37 (4.09 - 114.88) | -8.88 (-9.46 to -8.30) |
| Sri Lanka | 920 (111 - 1,833) | 223.04 (26.79 - 445.19) | 211 (24 - 454) | 42.91 (4.92 - 94.39) | -5.49 (-5.79 to -5.19) |
| Sudan | 74 (7 - 177) | 0.92 (0.09 - 2.19) | 285 (30 - 660) | 1.49 (0.16 - 3.47) | 2.86 (2.44 - 3.27) |
| Suriname | 78 (8 - 191) | 1.33 (0.14 - 3.41) | 130 (13 - 307) | 1.32 (0.12 - 3.19) | 0.53 (0.10 - 0.97) |
| Sweden | 8,380 (1,046 - 18,905) | 41.32 (5.23 - 91.69) | 555 (70 - 1,280) | 4.35 (0.55 - 10.28) | -6.68 (-7.36 to -5.99) |
| Switzerland | 2,676 (432 - 4,701) | 1480.16 (234.67 - 2602.35) | 239 (35 - 459) | 125.32 (18.13 - 246.14) | -8.02 (-8.43 to -7.61) |
| Syrian Arab Republic | 608 (63 - 1,405) | 3.63 (0.38 - 8.30) | 285 (31 - 704) | 1.32 (0.14 - 3.28) | -2.04 (-2.98 to -1.09) |
| Taiwan (Province of China) | 41,523 (5,523 - 85,665) | 434.47 (57.79 - 896.48) | 12,990 (1,623 - 29,095) | 97.72 (12.21 - 217.13) | -5.59 (-6.17 to -5.02) |
| Tajikistan | 698,507 (98,635 - 1,312,368) | 1926.59 (285.52 - 3555.91) | 268,551 (35,051 - 517,381) | 470.21 (61.41 - 910.64) | -4.52 (-4.73 to -4.30) |
| Thailand | 70,284 (6,690 - 188,132) | 157.63 (15.14 - 447.40) | 14,217 (1,636 - 32,836) | 20.05 (2.36 - 45.56) | -6.16 (-6.56 to -5.75) |
| Timor-Leste | 24,247 (4,043 - 44,890) | 1649.33 (273.51 - 3002.96) | 9,575 (1,522 - 17,018) | 393.21 (59.92 - 724.95) | -4.71 (-4.92 to -4.50) |
| Togo | 14,558 (1,759 - 31,688) | 1140.85 (138.14 - 2496.86) | 924 (102 - 2,090) | 60.29 (6.93 - 138.89) | -9.92 (-10.38 to -9.47) |
| Tokelau | 126,615 (18,537 - 234,136) | 2384.27 (349.07 - 4425.92) | 57,121 (7,749 - 115,993) | 678.13 (91.15 - 1309.84) | -3.75 (-4.07 to -3.42) |
| Tonga | 3 (0 - 6) | 190.98 (24.95 - 382.77) | 1 (0 - 3) | 130.84 (12.91 - 300.69) | -3.85 (-5.10 to -2.58) |
| Trinidad and Tobago | 40 (5 - 84) | 40.19 (5.20 - 88.16) | 23 (3 - 48) | 21.68 (2.63 - 44.94) | -1.46 (-1.91 to -1.02) |
| Tunisia | 473 (50 - 982) | 42.10 (4.45 - 87.48) | 95 (12 - 215) | 7.98 (1.03 - 18.28) | -5.88 (-6.37 to -5.39) |
| Turkey | 3,351 (423 - 7,542) | 33.64 (4.21 - 77.02) | 472 (54 - 1,123) | 4.57 (0.52 - 10.79) | -5.71 (-6.28 to -5.14) |
| Turkmenistan | 61,394 (6,529 - 139,321) | 89.73 (9.54 - 202.46) | 3,332 (410 - 7,750) | 5.03 (0.62 - 11.68) | -8.97 (-9.14 to -8.80) |
| Tuvalu | 3,392 (313 - 7,887) | 58.02 (5.35 - 134.96) | 56 (6 - 138) | 1.06 (0.11 - 2.59) | -14.48 (-15.36 to -13.60) |
| Uganda | 27 (3 - 55) | 248.58 (30.63 - 527.61) | 6 (1 - 13) | 56.56 (6.67 - 123.56) | -4.18 (-4.86 to -3.48) |
| Ukraine | 530,925 (77,455 - 1,127,572) | 1930.03 (288.52 - 4145.03) | 189,119 (25,912 - 380,153) | 434.32 (59.13 - 840.56) | -4.98 (-5.12 to -4.84) |
| United Arab Emirates | 647 (73 - 1,569) | 1.32 (0.14 - 3.19) | 554 (58 - 1,295) | 0.72 (0.08 - 1.75) | -1.37 (-2.69 to -0.03) |
| United Kingdom | 1,530 (180 - 3,619) | 4.13 (0.49 - 9.70) | 291 (32 - 696) | 1.00 (0.11 - 2.37) | -6.26 (-6.96 to -5.56) |
| United Republic of Tanzania | 161 (17 - 366) | 8.73 (0.91 - 19.67) | 129 (16 - 325) | 2.31 (0.27 - 5.79) | -2.99 (-3.39 to -2.59) |
| United States of America | 243 (26 - 543) | 8.28 (0.89 - 18.49) | 52 (6 - 124) | 1.41 (0.17 - 3.36) | -5.42 (-5.70 to -5.14) |
| United States Virgin Islands | 597 (73 - 1,441) | 0.26 (0.03 - 0.64) | 1,246 (124 - 2,968) | 0.25 (0.02 - 0.58) | 1.69 (0.74 - 2.65) |
| Uruguay | 33,973 (4,002 - 71,173) | 102.11 (12.05 - 213.64) | 464 (48 - 1,045) | 1.26 (0.13 - 2.82) | -15.10 (-15.75 to -14.44) |
| Uzbekistan | 519 (70 - 1,078) | 357.21 (47.88 - 724.49) | 288 (34 - 601) | 114.92 (13.72 - 239.57) | -3.62 (-3.74 to -3.49) |
| Vanuatu | 26,596 (3,261 - 58,725) | 112.43 (13.83 - 247.23) | 4,538 (481 - 11,154) | 18.82 (1.99 - 46.50) | -6.13 (-6.91 to -5.34) |
| Venezuela (Bolivarian Republic of) | 56,048 (6,500 - 134,343) | 76.58 (8.58 - 182.62) | 6,695 (744 - 16,278) | 7.29 (0.80 - 17.78) | -7.12 (-7.49 to -6.75) |
| Viet Nam | 14 (2 - 29) | 13.04 (1.43 - 27.80) | 4 (1 - 9) | 5.32 (0.70 - 11.65) | -2.64 (-2.70 to -2.57) |
| Yemen | 342,506 (56,153 - 651,572) | 1242.53 (204.91 - 2370.56) | 26,480 (3,461 - 62,374) | 64.67 (8.34 - 150.49) | -9.20 (-9.56 to -8.84) |
| Zambia | 310,089 (45,369 - 590,263) | 2587.65 (383.05 - 4821.88) | 95,570 (13,408 - 182,401) | 499.97 (71.11 - 961.72) | -5.40 (-5.94 to -4.86) |
| Zimbabwe | 61,215 (8,186 - 116,848) | 517.87 (64.30 - 1025.38) | 65,584 (7,934 - 137,583) | 432.38 (50.04 - 926.58) | 0.36 (-0.40 - 1.12) |

| Table S2. The DALYs and age-standardized DALYs Rate of no access to handwashing in 1990 and 2021, with Temporal Trends from 1990 to 2021 in 204 countries or territories. | | | | | |
| --- | --- | --- | --- | --- | --- |
| **location** | **Num_1990** | **DALYs_1990** | **Num_2021** | **DALYs_2021** | **EAPC_CI** |
| Afghanistan | 1,836 (247 - 3,516) | 10.15 (1.36 - 19.84) | 887 (131 - 1,837) | 1.69 (0.26 - 3.42) | -6.09 (-6.81 to -5.35) |
| Albania | 7 (1 - 15) | 0.18 (0.02 - 0.42) | 1 (0 - 1) | 0.02 (0.00 - 0.06) | -7.21 (-7.89 to -6.54) |
| Algeria | 120 (14 - 291) | 0.46 (0.06 - 1.08) | 26 (3 - 61) | 0.08 (0.01 - 0.19) | -5.06 (-5.18 to -4.94) |
| American Samoa | 0 (0 - 1) | 1.75 (0.17 - 4.20) | 0 (0 - 1) | 1.19 (0.12 - 2.81) | -0.95 (-1.45 to -0.45) |
| Andorra | 0 (0 - 0) | 0.00 (0.00 - 0.01) | 0 (0 - 0) | 0.00 (0.00 - 0.00) | -1.32 (-1.47 to -1.17) |
| Angola | 7,826 (1,216 - 14,384) | 79.40 (13.32 - 146.87) | 2,030 (272 - 3,995) | 12.50 (1.65 - 24.98) | -5.87 (-6.32 to -5.42) |
| Antigua and Barbuda | 0 (0 - 1) | 0.60 (0.07 - 1.24) | 0 (0 - 0) | 0.12 (0.01 - 0.25) | -5.34 (-6.17 to -4.51) |
| Argentina | 61 (6 - 135) | 0.19 (0.02 - 0.43) | 12 (1 - 29) | 0.02 (0.00 - 0.06) | -5.74 (-6.45 to -5.03) |
| Armenia | 32 (4 - 68) | 0.87 (0.11 - 1.87) | 0 (0 - 1) | 0.01 (0.00 - 0.02) | -16.13 (-17.03 to -15.22) |
| Australia | 2 (0 - 4) | 0.01 (0.00 - 0.02) | 2 (0 - 5) | 0.00 (0.00 - 0.01) | -1.13 (-2.21 to -0.05) |
| Austria | 0 (0 - 1) | 0.00 (0.00 - 0.01) | 3 (0 - 6) | 0.01 (0.00 - 0.03) | 4.48 (3.67 - 5.30) |
| Azerbaijan | 105 (12 - 241) | 1.22 (0.14 - 2.77) | 7 (1 - 18) | 0.10 (0.01 - 0.26) | -8.91 (-9.48 to -8.34) |
| Bahamas | 1 (0 - 1) | 0.31 (0.04 - 0.67) | 0 (0 - 0) | 0.07 (0.01 - 0.15) | -5.62 (-6.47 to -4.75) |
| Bahrain | 1 (0 - 1) | 0.21 (0.02 - 0.46) | 0 (0 - 1) | 0.05 (0.00 - 0.13) | -4.61 (-4.75 to -4.48) |
| Bangladesh | 23,239 (3,456 - 43,919) | 31.58 (4.58 - 59.39) | 5,894 (735 - 16,101) | 5.17 (0.66 - 13.97) | -5.91 (-6.01 to -5.81) |
| Barbados | 1 (0 - 1) | 0.23 (0.03 - 0.50) | 0 (0 - 1) | 0.06 (0.01 - 0.14) | -4.33 (-4.98 to -3.69) |
| Belarus | 3 (0 - 7) | 0.03 (0.00 - 0.08) | 0 (0 - 1) | 0.00 (0.00 - 0.01) | -10.59 (-11.72 to -9.45) |
| Belgium | 3 (0 - 7) | 0.02 (0.00 - 0.05) | 18 (2 - 42) | 0.06 (0.01 - 0.14) | 4.17 (3.30 - 5.05) |
| Belize | 7 (1 - 12) | 2.89 (0.40 - 5.42) | 2 (0 - 3) | 0.48 (0.06 - 0.95) | -5.69 (-6.18 to -5.20) |
| Benin | 2,382 (379 - 4,312) | 58.46 (8.76 - 108.42) | 1,272 (168 - 2,623) | 16.03 (2.11 - 31.83) | -4.14 (-4.26 to -4.02) |
| Bermuda | 0 (0 - 0) | 0.17 (0.02 - 0.37) | 0 (0 - 0) | 0.02 (0.00 - 0.05) | -6.60 (-7.42 to -5.78) |
| Bhutan | 192 (23 - 444) | 50.68 (6.22 - 119.58) | 12 (1 - 33) | 2.19 (0.25 - 6.13) | -10.57 (-10.91 to -10.23) |
| Bolivia (Plurinational State of) | 390 (48 - 815) | 5.09 (0.64 - 10.70) | 51 (6 - 122) | 0.54 (0.06 - 1.30) | -7.37 (-7.53 to -7.21) |
| Bosnia and Herzegovina | 2 (0 - 4) | 0.05 (0.00 - 0.11) | 1 (0 - 2) | 0.02 (0.00 - 0.04) | -3.46 (-4.04 to -2.87) |
| Botswana | 299 (40 - 568) | 31.51 (3.90 - 63.34) | 145 (19 - 292) | 8.47 (1.07 - 17.12) | -4.00 (-4.19 to -3.80) |
| Brazil | 5,733 (775 - 10,997) | 4.29 (0.58 - 8.23) | 408 (40 - 899) | 0.18 (0.02 - 0.40) | -10.02 (-10.38 to -9.65) |
| Brunei Darussalam | 0 (0 - 0) | 0.02 (0.00 - 0.06) | 0 (0 - 0) | 0.01 (0.00 - 0.03) | -1.34 (-1.66 to -1.03) |
| Bulgaria | 1 (0 - 3) | 0.02 (0.00 - 0.05) | 2 (0 - 5) | 0.02 (0.00 - 0.05) | 0.45 (-1.10 - 2.02) |
| Burkina Faso | 6,015 (847 - 10,941) | 63.32 (9.60 - 116.34) | 3,486 (549 - 6,189) | 19.78 (2.95 - 35.66) | -3.81 (-3.92 to -3.70) |
| Burundi | 2,117 (306 - 3,962) | 45.00 (6.46 - 86.80) | 1,523 (188 - 3,076) | 21.99 (2.63 - 44.89) | -2.34 (-2.45 to -2.22) |
| Cabo Verde | 1,502 (197 - 3,112) | 18.00 (2.33 - 37.87) | 129 (13 - 306) | 1.28 (0.13 - 3.10) | -9.01 (-9.38 to -8.65) |
| Cambodia | 3,155 (479 - 6,147) | 33.58 (5.07 - 68.24) | 2,098 (259 - 4,456) | 9.84 (1.23 - 19.67) | -3.76 (-4.17 to -3.34) |
| Cameroon | 1 (0 - 2) | 0.00 (0.00 - 0.01) | 8 (1 - 19) | 0.01 (0.00 - 0.02) | 6.81 (4.62 - 9.05) |
| Canada | 74 (11 - 134) | 18.74 (2.79 - 35.18) | 13 (2 - 26) | 2.90 (0.41 - 5.92) | -6.30 (-6.80 to -5.79) |
| Central African Republic | 1,463 (237 - 2,648) | 55.12 (9.33 - 104.15) | 1,239 (166 - 2,520) | 34.25 (4.78 - 69.73) | -1.25 (-1.38 to -1.12) |
| Chad | 6,300 (962 - 11,665) | 95.10 (14.58 - 185.97) | 7,985 (1,144 - 15,762) | 46.44 (6.61 - 84.89) | -2.29 (-2.44 to -2.13) |
| Chile | 13 (1 - 30) | 0.12 (0.01 - 0.29) | 5 (1 - 12) | 0.02 (0.00 - 0.05) | -4.62 (-5.26 to -3.98) |
| China | 11,062 (1,246 - 22,728) | 1.12 (0.13 - 2.35) | 159 (16 - 361) | 0.01 (0.00 - 0.03) | -14.79 (-15.26 to -14.31) |
| Colombia | 389 (47 - 819) | 1.19 (0.15 - 2.52) | 55 (7 - 130) | 0.11 (0.01 - 0.26) | -8.39 (-9.00 to -7.78) |
| Comoros | 99 (14 - 191) | 23.51 (3.34 - 45.98) | 42 (5 - 86) | 8.21 (0.93 - 17.44) | -3.26 (-3.46 to -3.05) |
| Congo | 588 (82 - 1,163) | 29.56 (4.06 - 58.34) | 208 (24 - 478) | 7.27 (0.87 - 16.62) | -4.31 (-4.71 to -3.91) |
| Cook Islands | 0 (0 - 0) | 0.37 (0.04 - 0.89) | 0 (0 - 0) | 0.15 (0.01 - 0.37) | -2.60 (-3.11 to -2.09) |
| Costa Rica | 12 (1 - 27) | 0.49 (0.05 - 1.09) | 3 (0 - 8) | 0.06 (0.01 - 0.15) | -6.36 (-6.93 to -5.78) |
| Côte d'Ivoire | 0 (0 - 1) | 0.01 (0.00 - 0.03) | 1 (0 - 3) | 0.02 (0.00 - 0.04) | 2.48 (0.60 - 4.40) |
| Croatia | 48 (6 - 100) | 0.52 (0.07 - 1.07) | 16 (2 - 38) | 0.09 (0.01 - 0.22) | -6.35 (-7.12 to -5.57) |
| Cuba | 1 (0 - 2) | 0.15 (0.01 - 0.38) | 1 (0 - 2) | 0.05 (0.00 - 0.13) | -3.35 (-3.69 to -3.01) |
| Cyprus | 2 (0 - 5) | 0.02 (0.00 - 0.05) | 8 (1 - 18) | 0.04 (0.00 - 0.10) | 4.06 (1.85 - 6.32) |
| Czechia | 3,275 (471 - 5,986) | 30.31 (4.49 - 55.83) | 1,700 (214 - 3,326) | 9.19 (1.20 - 17.52) | -3.36 (-3.64 to -3.08) |
| Democratic People's Republic of Korea | 2 (0 - 4) | 0.02 (0.00 - 0.05) | 7 (1 - 18) | 0.05 (0.00 - 0.13) | 3.74 (2.93 - 4.55) |
| Democratic Republic of the Congo | 119 (19 - 220) | 36.11 (5.34 - 69.06) | 61 (7 - 121) | 9.11 (1.04 - 18.94) | -4.42 (-4.59 to -4.24) |
| Denmark | 1 (0 - 1) | 0.83 (0.08 - 1.73) | 0 (0 - 0) | 0.26 (0.03 - 0.61) | -3.71 (-4.26 to -3.16) |
| Djibouti | 472 (68 - 920) | 5.65 (0.78 - 11.10) | 96 (13 - 214) | 0.96 (0.13 - 2.15) | -5.55 (-5.91 to -5.18) |
| Dominica | 13,130 (1,985 - 23,747) | 35.83 (5.86 - 64.33) | 4,865 (619 - 10,043) | 9.85 (1.30 - 20.23) | -4.17 (-5.08 to -3.26) |
| Dominican Republic | 295 (38 - 620) | 3.07 (0.40 - 6.45) | 16 (2 - 35) | 0.10 (0.01 - 0.23) | -10.80 (-11.18 to -10.42) |
| Ecuador | 3,387 (417 - 7,234) | 4.58 (0.58 - 9.65) | 171 (17 - 413) | 0.20 (0.02 - 0.49) | -9.54 (-9.98 to -9.09) |
| Egypt | 336 (39 - 679) | 5.63 (0.65 - 11.79) | 19 (2 - 47) | 0.29 (0.03 - 0.72) | -9.54 (-10.29 to -8.79) |
| El Salvador | 288 (43 - 551) | 69.00 (11.12 - 142.04) | 24 (3 - 53) | 3.65 (0.40 - 8.25) | -10.46 (-10.91 to -10.00) |
| Equatorial Guinea | 1,966 (276 - 3,612) | 76.87 (11.40 - 154.71) | 768 (91 - 1,736) | 22.20 (2.30 - 51.65) | -3.95 (-4.04 to -3.86) |
| Eritrea | 0 (0 - 1) | 0.02 (0.00 - 0.05) | 0 (0 - 0) | 0.00 (0.00 - 0.00) | -10.50 (-11.53 to -9.46) |
| Estonia | 294 (44 - 546) | 43.52 (6.69 - 83.35) | 89 (13 - 181) | 10.81 (1.46 - 23.17) | -4.15 (-4.55 to -3.74) |
| Eswatini | 27,739 (4,461 - 55,827) | 76.33 (11.13 - 156.39) | 11,519 (1,766 - 20,710) | 19.65 (2.72 - 36.63) | -4.66 (-4.85 to -4.48) |
| Ethiopia | 3 (0 - 7) | 6.41 (0.74 - 14.46) | 1 (0 - 3) | 2.47 (0.29 - 5.65) | -2.82 (-3.56 to -2.07) |
| Fiji | 17 (2 - 36) | 5.71 (0.62 - 12.74) | 13 (1 - 29) | 2.57 (0.27 - 5.88) | -2.17 (-2.56 to -1.78) |
| Finland | 1 (0 - 2) | 0.01 (0.00 - 0.03) | 2 (0 - 5) | 0.01 (0.00 - 0.03) | 0.75 (0.02 - 1.48) |
| France | 29 (3 - 66) | 0.03 (0.00 - 0.08) | 26 (3 - 59) | 0.01 (0.00 - 0.03) | -3.21 (-4.11 to -2.30) |
| Gabon | 188 (26 - 387) | 22.86 (3.37 - 47.32) | 36 (4 - 88) | 3.46 (0.41 - 8.66) | -6.02 (-6.22 to -5.83) |
| Gambia | 10 (1 - 23) | 0.23 (0.02 - 0.53) | 0 (0 - 0) | 0.00 (0.00 - 0.01) | -14.06 (-14.93 to -13.17) |
| Georgia | 14 (1 - 33) | 0.01 (0.00 - 0.03) | 57 (6 - 134) | 0.02 (0.00 - 0.06) | 5.08 (3.70 - 6.48) |
| Germany | 3,751 (637 - 6,946) | 32.75 (5.72 - 64.60) | 1,613 (234 - 3,046) | 8.68 (1.21 - 17.07) | -4.14 (-4.27 to -4.02) |
| Ghana | 0 (0 - 1) | 0.00 (0.00 - 0.00) | 2 (0 - 4) | 0.01 (0.00 - 0.01) | 4.13 (3.47 - 4.79) |
| Greece | 0 (0 - 0) | 0.02 (0.00 - 0.06) | 0 (0 - 0) | 0.01 (0.00 - 0.04) | -1.45 (-1.58 to -1.32) |
| Greenland | 1 (0 - 1) | 0.73 (0.09 - 1.41) | 0 (0 - 0) | 0.10 (0.01 - 0.23) | -6.26 (-7.04 to -5.48) |
| Grenada | 0 (0 - 1) | 0.47 (0.05 - 1.16) | 0 (0 - 1) | 0.16 (0.02 - 0.39) | -3.01 (-3.85 to -2.17) |
| Guam | 1,640 (219 - 3,170) | 23.17 (3.09 - 45.23) | 224 (28 - 480) | 1.87 (0.23 - 3.97) | -7.63 (-8.04 to -7.22) |
| Guatemala | 4,203 (678 - 8,061) | 76.88 (12.92 - 161.70) | 1,235 (158 - 2,564) | 16.59 (2.09 - 34.08) | -4.87 (-5.06 to -4.68) |
| Guinea | 529 (84 - 974) | 63.70 (9.78 - 122.66) | 172 (23 - 328) | 17.12 (2.20 - 33.02) | -4.23 (-4.60 to -3.85) |
| Guinea-Bissau | 45 (6 - 89) | 5.91 (0.76 - 11.43) | 7 (1 - 15) | 1.16 (0.16 - 2.38) | -5.17 (-5.49 to -4.85) |
| Guyana | 2,854 (404 - 5,087) | 32.57 (4.67 - 60.80) | 1,167 (172 - 2,190) | 9.59 (1.34 - 19.34) | -3.70 (-4.14 to -3.27) |
| Haiti | 334 (40 - 678) | 7.28 (0.87 - 15.19) | 66 (7 - 173) | 1.02 (0.10 - 2.60) | -6.19 (-6.43 to -5.95) |
| Honduras | 2 (0 - 4) | 0.02 (0.00 - 0.05) | 6 (1 - 15) | 0.04 (0.00 - 0.09) | 3.65 (1.53 - 5.81) |
| Hungary | 0 (0 - 0) | 0.01 (0.00 - 0.02) | 0 (0 - 0) | 0.01 (0.00 - 0.03) | 3.31 (2.34 - 4.28) |
| Iceland | 309,547 (46,156 - 582,291) | 64.71 (9.51 - 122.45) | 77,612 (9,020 - 172,741) | 8.02 (0.93 - 17.93) | -6.56 (-6.82 to -6.29) |
| India | 27,160 (3,284 - 60,308) | 24.10 (2.83 - 55.68) | 3,526 (298 - 8,932) | 2.18 (0.18 - 5.82) | -7.09 (-7.35 to -6.83) |
| Indonesia | 414 (40 - 968) | 0.70 (0.07 - 1.59) | 25 (2 - 60) | 0.04 (0.00 - 0.09) | -8.74 (-8.99 to -8.49) |
| Iran (Islamic Republic of) | 205 (21 - 478) | 0.80 (0.08 - 1.83) | 23 (2 - 56) | 0.08 (0.01 - 0.20) | -7.93 (-8.16 to -7.70) |
| Iraq | 0 (0 - 1) | 0.01 (0.00 - 0.02) | 1 (0 - 2) | 0.01 (0.00 - 0.02) | 2.34 (1.57 - 3.11) |
| Ireland | 1 (0 - 3) | 0.03 (0.00 - 0.06) | 7 (1 - 16) | 0.05 (0.01 - 0.11) | 3.20 (2.30 - 4.11) |
| Israel | 3 (0 - 7) | 0.00 (0.00 - 0.01) | 42 (5 - 99) | 0.02 (0.00 - 0.05) | 9.52 (7.55 - 11.53) |
| Italy | 40 (5 - 79) | 1.71 (0.22 - 3.40) | 8 (1 - 18) | 0.28 (0.03 - 0.60) | -5.08 (-6.16 to -3.99) |
| Jamaica | 14 (1 - 32) | 0.01 (0.00 - 0.03) | 36 (3 - 86) | 0.01 (0.00 - 0.02) | -0.72 (-1.12 to -0.31) |
| Japan | 9 (1 - 20) | 0.21 (0.02 - 0.47) | 3 (0 - 7) | 0.04 (0.00 - 0.10) | -5.38 (-5.52 to -5.24) |
| Jordan | 47 (5 - 106) | 0.26 (0.03 - 0.60) | 1 (0 - 1) | 0.00 (0.00 - 0.01) | -16.16 (-17.03 to -15.28) |
| Kazakhstan | 7,541 (1,112 - 14,094) | 40.51 (6.55 - 73.79) | 3,547 (510 - 6,654) | 12.73 (1.83 - 24.96) | -3.28 (-3.62 to -2.95) |
| Kenya | 13 (2 - 27) | 35.30 (4.70 - 75.09) | 7 (1 - 15) | 14.02 (1.68 - 30.89) | -2.67 (-2.92 to -2.41) |
| Kiribati | 1 (0 - 1) | 0.05 (0.01 - 0.10) | 0 (0 - 0) | 0.01 (0.00 - 0.02) | -3.68 (-4.67 to -2.69) |
| Kuwait | 48 (6 - 114) | 0.80 (0.09 - 1.89) | 3 (0 - 7) | 0.04 (0.00 - 0.09) | -10.66 (-11.46 to -9.86) |
| Kyrgyzstan | 1,463 (207 - 3,028) | 34.08 (4.78 - 70.24) | 131 (16 - 280) | 2.62 (0.32 - 5.91) | -8.37 (-8.59 to -8.16) |
| Lao People's Democratic Republic | 0 (0 - 1) | 0.02 (0.00 - 0.05) | 0 (0 - 0) | 0.00 (0.00 - 0.00) | -10.17 (-11.16 to -9.18) |
| Latvia | 12 (2 - 29) | 0.44 (0.06 - 1.03) | 5 (1 - 13) | 0.09 (0.01 - 0.22) | -5.06 (-5.22 to -4.89) |
| Lebanon | 829 (133 - 1,497) | 66.38 (10.44 - 125.73) | 419 (60 - 823) | 30.94 (4.38 - 62.44) | -2.09 (-2.35 to -1.84) |
| Lesotho | 1,654 (259 - 2,859) | 53.73 (8.74 - 93.35) | 510 (70 - 1,006) | 14.24 (2.02 - 27.16) | -4.75 (-5.02 to -4.48) |
| Liberia | 45 (5 - 101) | 1.02 (0.11 - 2.34) | 4 (0 - 9) | 0.09 (0.01 - 0.21) | -7.24 (-8.27 to -6.19) |
| Libya | 1 (0 - 2) | 0.03 (0.00 - 0.08) | 0 (0 - 0) | 0.00 (0.00 - 0.01) | -8.94 (-10.09 to -7.78) |
| Lithuania | 0 (0 - 0) | 0.02 (0.00 - 0.04) | 0 (0 - 1) | 0.03 (0.00 - 0.07) | 2.79 (1.99 - 3.59) |
| Luxembourg | 6,062 (957 - 10,547) | 45.98 (7.58 - 81.04) | 4,419 (569 - 8,460) | 20.99 (2.92 - 42.41) | -2.11 (-2.32 to -1.90) |
| Madagascar | 6,759 (943 - 12,326) | 69.65 (11.08 - 132.16) | 2,116 (294 - 3,835) | 21.37 (2.88 - 39.64) | -3.91 (-4.02 to -3.81) |
| Malawi | 42 (4 - 104) | 0.38 (0.04 - 0.94) | 22 (2 - 57) | 0.10 (0.01 - 0.25) | -4.22 (-4.70 to -3.73) |
| Malaysia | 13 (2 - 32) | 6.06 (0.72 - 14.86) | 0 (0 - 1) | 0.13 (0.01 - 0.32) | -11.93 (-12.78 to -11.08) |
| Maldives | 5,082 (816 - 9,625) | 67.37 (10.69 - 126.90) | 2,524 (350 - 4,935) | 18.47 (2.40 - 39.45) | -4.08 (-4.28 to -3.88) |
| Mali | 0 (0 - 0) | 0.00 (0.00 - 0.01) | 0 (0 - 0) | 0.01 (0.00 - 0.02) | 2.44 (1.61 - 3.29) |
| Malta | 1 (0 - 3) | 8.05 (0.96 - 18.45) | 1 (0 - 2) | 3.10 (0.41 - 7.18) | -2.63 (-3.11 to -2.15) |
| Marshall Islands | 605 (100 - 1,162) | 33.42 (5.19 - 62.17) | 199 (26 - 396) | 6.83 (0.90 - 13.50) | -5.06 (-5.34 to -4.79) |
| Mauritania | 2 (0 - 4) | 0.21 (0.02 - 0.52) | 1 (0 - 2) | 0.05 (0.01 - 0.11) | -3.09 (-3.60 to -2.58) |
| Mauritius | 2,689 (360 - 5,859) | 3.48 (0.46 - 7.59) | 239 (26 - 548) | 0.21 (0.02 - 0.49) | -8.34 (-9.11 to -7.56) |
| Mexico | 11 (1 - 24) | 0.28 (0.03 - 0.59) | 1 (0 - 1) | 0.02 (0.00 - 0.05) | -9.60 (-10.33 to -8.87) |
| Micronesia (Federated States of) | 0 (0 - 0) | 0.00 (0.00 - 0.01) | 0 (0 - 0) | 0.00 (0.00 - 0.01) | 0.87 (0.60 - 1.13) |
| Monaco | 32 (4 - 74) | 0.96 (0.12 - 2.18) | 3 (0 - 8) | 0.07 (0.01 - 0.21) | -8.33 (-8.54 to -8.11) |
| Mongolia | 0 (0 - 0) | 0.00 (0.00 - 0.01) | 0 (0 - 0) | 0.00 (0.00 - 0.00) | -3.84 (-4.07 to -3.61) |
| Montenegro | 1,951 (269 - 4,079) | 5.80 (0.80 - 11.83) | 75 (8 - 181) | 0.25 (0.03 - 0.60) | -9.65 (-9.84 to -9.45) |
| Morocco | 6,261 (988 - 11,837) | 48.16 (7.16 - 95.96) | 2,554 (334 - 5,189) | 14.33 (1.73 - 30.22) | -3.71 (-3.86 to -3.56) |
| Mozambique | 5,583 (686 - 12,982) | 16.63 (2.08 - 40.34) | 473 (48 - 1,047) | 1.11 (0.11 - 2.53) | -8.88 (-9.09 to -8.66) |
| Myanmar | 376 (50 - 740) | 36.30 (4.48 - 72.80) | 158 (20 - 330) | 9.29 (1.15 - 19.88) | -4.25 (-4.46 to -4.04) |
| Namibia | 0 (0 - 0) | 3.49 (0.38 - 7.75) | 0 (0 - 0) | 1.70 (0.20 - 4.28) | -2.06 (-2.34 to -1.77) |
| Nauru | 5,257 (799 - 9,745) | 35.40 (5.18 - 70.55) | 661 (92 - 1,528) | 3.43 (0.46 - 8.33) | -7.61 (-7.79 to -7.43) |
| Nepal | 1 (0 - 3) | 0.01 (0.00 - 0.02) | 9 (1 - 21) | 0.02 (0.00 - 0.05) | 4.44 (3.57 - 5.31) |
| Netherlands | 0 (0 - 1) | 0.01 (0.00 - 0.03) | 1 (0 - 3) | 0.01 (0.00 - 0.03) | 2.50 (0.78 - 4.24) |
| New Zealand | 336 (43 - 680) | 6.42 (0.82 - 13.26) | 19 (2 - 44) | 0.36 (0.04 - 0.82) | -8.90 (-9.26 to -8.54) |
| Nicaragua | 8,759 (1,383 - 15,650) | 103.00 (16.51 - 193.33) | 5,016 (699 - 9,742) | 30.36 (4.20 - 57.86) | -4.23 (-4.55 to -3.91) |
| Niger | 60,882 (9,378 - 109,298) | 58.73 (9.48 - 111.30) | 36,266 (5,057 - 64,989) | 16.44 (2.38 - 29.96) | -4.10 (-4.50 to -3.70) |
| Nigeria | 0 (0 - 0) | 2.01 (0.23 - 4.81) | 0 (0 - 0) | 0.96 (0.11 - 2.55) | -2.93 (-3.56 to -2.29) |
| Niue | 9 (1 - 22) | 0.05 (0.00 - 0.11) | 4 (0 - 9) | 0.01 (0.00 - 0.04) | -3.52 (-3.73 to -3.30) |
| North Macedonia | 5 (1 - 11) | 0.31 (0.03 - 0.67) | 1 (0 - 2) | 0.03 (0.00 - 0.09) | -6.04 (-6.85 to -5.22) |
| Northern Mariana Islands | 0 (0 - 0) | 0.82 (0.09 - 2.04) | 0 (0 - 1) | 0.73 (0.07 - 1.77) | 0.15 (-0.47 - 0.77) |
| Norway | 1 (0 - 3) | 0.01 (0.00 - 0.03) | 6 (1 - 15) | 0.05 (0.01 - 0.12) | 5.63 (4.48 - 6.80) |
| Oman | 13 (2 - 31) | 0.70 (0.08 - 1.62) | 1 (0 - 3) | 0.05 (0.01 - 0.14) | -7.96 (-8.63 to -7.28) |
| Pakistan | 25,865 (3,502 - 50,486) | 28.01 (3.98 - 62.20) | 5,502 (636 - 12,098) | 4.20 (0.51 - 9.82) | -5.87 (-6.16 to -5.58) |
| Palau | 0 (0 - 1) | 3.36 (0.39 - 7.52) | 0 (0 - 1) | 1.84 (0.20 - 4.58) | -1.53 (-2.10 to -0.95) |
| Palestine | 28 (3 - 60) | 0.98 (0.11 - 2.02) | 2 (0 - 4) | 0.05 (0.01 - 0.12) | -8.93 (-9.17 to -8.68) |
| Panama | 27 (3 - 58) | 1.29 (0.13 - 2.81) | 7 (1 - 15) | 0.16 (0.02 - 0.36) | -5.70 (-6.17 to -5.23) |
| Papua New Guinea | 645 (81 - 1,188) | 26.18 (3.44 - 51.47) | 676 (89 - 1,357) | 11.32 (1.40 - 22.28) | -2.27 (-2.49 to -2.05) |
| Paraguay | 130 (16 - 266) | 3.07 (0.40 - 6.61) | 10 (1 - 24) | 0.17 (0.02 - 0.41) | -9.61 (-10.12 to -9.10) |
| Peru | 650 (78 - 1,371) | 2.79 (0.34 - 5.90) | 58 (6 - 141) | 0.17 (0.02 - 0.42) | -9.42 (-9.92 to -8.92) |
| Philippines | 2,414 (328 - 5,179) | 4.23 (0.56 - 9.43) | 457 (50 - 1,032) | 0.57 (0.06 - 1.34) | -5.84 (-6.01 to -5.67) |
| Poland | 4 (0 - 9) | 0.01 (0.00 - 0.03) | 17 (2 - 41) | 0.03 (0.00 - 0.06) | 4.67 (2.18 - 7.22) |
| Portugal | 2 (0 - 4) | 0.02 (0.00 - 0.05) | 8 (1 - 19) | 0.03 (0.00 - 0.06) | 1.91 (1.31 - 2.50) |
| Puerto Rico | 6 (1 - 12) | 0.18 (0.02 - 0.37) | 5 (1 - 11) | 0.07 (0.01 - 0.16) | -3.65 (-4.74 to -2.55) |
| Qatar | 0 (0 - 0) | 0.09 (0.01 - 0.20) | 0 (0 - 0) | 0.02 (0.00 - 0.05) | -4.61 (-4.79 to -4.42) |
| Republic of Korea | 20 (2 - 45) | 0.13 (0.01 - 0.28) | 9 (1 - 21) | 0.03 (0.00 - 0.07) | -4.59 (-6.16 to -3.00) |
| Republic of Moldova | 65 (8 - 151) | 0.06 (0.01 - 0.13) | 9 (1 - 23) | 0.01 (0.00 - 0.01) | -8.91 (-9.59 to -8.23) |
| Romania | 2,550 (385 - 4,639) | 34.92 (5.14 - 69.96) | 790 (114 - 1,583) | 9.42 (1.29 - 21.64) | -4.99 (-5.48 to -4.50) |
| Russian Federation | 1 (0 - 2) | 2.48 (0.30 - 5.07) | 0 (0 - 0) | 0.39 (0.04 - 0.84) | -5.76 (-6.57 to -4.94) |
| Rwanda | 2 (0 - 3) | 1.49 (0.17 - 2.93) | 0 (0 - 1) | 0.19 (0.02 - 0.42) | -7.00 (-7.94 to -6.04) |
| Saint Kitts and Nevis | 2 (0 - 5) | 2.58 (0.35 - 5.00) | 0 (0 - 1) | 0.35 (0.04 - 0.73) | -6.16 (-7.00 to -5.32) |
| Saint Lucia | 1 (0 - 2) | 1.01 (0.12 - 2.45) | 0 (0 - 1) | 0.39 (0.04 - 0.92) | -3.21 (-3.63 to -2.79) |
| Saint Vincent and the Grenadines | 0 (0 - 0) | 0.00 (0.00 - 0.01) | 0 (0 - 0) | 0.00 (0.00 - 0.01) | -1.66 (-2.00 to -1.32) |
| Samoa | 141 (16 - 321) | 1.01 (0.12 - 2.26) | 11 (1 - 28) | 0.08 (0.01 - 0.23) | -7.86 (-8.18 to -7.54) |
| San Marino | 3,925 (607 - 6,957) | 55.53 (8.49 - 100.08) | 788 (106 - 1,545) | 8.23 (1.07 - 16.16) | -6.08 (-6.36 to -5.80) |
| Sao Tome and Principe | 2 (0 - 5) | 0.03 (0.00 - 0.07) | 1 (0 - 4) | 0.01 (0.00 - 0.03) | -3.37 (-3.82 to -2.93) |
| Saudi Arabia | 0 (0 - 1) | 0.35 (0.03 - 0.86) | 0 (0 - 0) | 0.14 (0.01 - 0.39) | -2.42 (-2.67 to -2.17) |
| Senegal | 2,315 (321 - 4,307) | 54.12 (7.95 - 104.30) | 980 (137 - 1,956) | 18.03 (2.60 - 35.82) | -3.71 (-4.14 to -3.28) |
| Serbia | 1 (0 - 1) | 0.03 (0.00 - 0.08) | 1 (0 - 1) | 0.01 (0.00 - 0.02) | -3.44 (-3.90 to -2.97) |
| Seychelles | 0 (0 - 1) | 0.01 (0.00 - 0.03) | 0 (0 - 1) | 0.01 (0.00 - 0.02) | -1.38 (-1.96 to -0.78) |
| Sierra Leone | 0 (0 - 0) | 0.00 (0.00 - 0.01) | 0 (0 - 1) | 0.01 (0.00 - 0.02) | 2.56 (0.48 - 4.69) |
| Singapore | 22 (3 - 44) | 14.61 (2.22 - 30.26) | 12 (2 - 25) | 4.19 (0.57 - 9.25) | -3.69 (-3.87 to -3.52) |
| Slovakia | 4,913 (776 - 9,539) | 75.85 (11.34 - 159.26) | 4,852 (740 - 9,333) | 39.74 (5.47 - 83.63) | -2.32 (-2.49 to -2.15) |
| Slovenia | 6,994 (1,032 - 13,001) | 20.48 (3.19 - 40.47) | 3,130 (380 - 6,155) | 7.24 (0.89 - 14.53) | -3.15 (-3.46 to -2.84) |
| Solomon Islands | 12 (1 - 32) | 0.06 (0.01 - 0.17) | 11 (1 - 29) | 0.01 (0.00 - 0.03) | -4.14 (-4.75 to -3.52) |
| Somalia | 4,009 (578 - 7,667) | 70.83 (10.88 - 143.24) | 3,543 (481 - 7,097) | 50.85 (7.61 - 106.43) | -0.98 (-1.10 to -0.86) |
| South Africa | 7 (1 - 18) | 0.02 (0.00 - 0.04) | 29 (3 - 68) | 0.02 (0.00 - 0.05) | 2.26 (1.83 - 2.70) |
| South Sudan | 340 (40 - 802) | 3.56 (0.40 - 8.83) | 32 (3 - 85) | 0.15 (0.01 - 0.38) | -9.54 (-10.14 to -8.94) |
| Spain | 2,904 (370 - 6,516) | 9.43 (1.22 - 20.33) | 219 (20 - 654) | 0.60 (0.05 - 1.63) | -8.68 (-9.25 to -8.11) |
| Sri Lanka | 13 (2 - 25) | 3.58 (0.43 - 7.19) | 4 (0 - 9) | 0.74 (0.08 - 1.61) | -5.16 (-5.43 to -4.88) |
| Sudan | 1 (0 - 1) | 0.00 (0.00 - 0.01) | 15 (2 - 36) | 0.05 (0.01 - 0.12) | 9.87 (8.61 - 11.15) |
| Suriname | 1 (0 - 3) | 0.01 (0.00 - 0.03) | 4 (0 - 10) | 0.02 (0.00 - 0.04) | 3.31 (2.54 - 4.08) |
| Sweden | 85 (10 - 195) | 0.52 (0.06 - 1.20) | 4 (0 - 12) | 0.05 (0.01 - 0.12) | -7.00 (-7.69 to -6.31) |
| Switzerland | 35 (6 - 62) | 26.02 (4.01 - 45.49) | 4 (1 - 8) | 3.21 (0.42 - 6.42) | -6.97 (-7.34 to -6.60) |
| Syrian Arab Republic | 7 (1 - 17) | 0.05 (0.01 - 0.12) | 1 (0 - 3) | 0.00 (0.00 - 0.01) | -7.67 (-8.73 to -6.60) |
| Taiwan (Province of China) | 459 (60 - 945) | 4.89 (0.64 - 10.03) | 143 (18 - 319) | 1.10 (0.14 - 2.42) | -5.62 (-6.21 to -5.03) |
| Tajikistan | 9,583 (1,384 - 17,776) | 44.71 (6.79 - 85.39) | 4,422 (562 - 8,636) | 12.78 (1.60 - 26.14) | -4.05 (-4.25 to -3.85) |
| Thailand | 1,521 (141 - 4,908) | 4.81 (0.43 - 16.17) | 470 (48 - 1,117) | 0.49 (0.05 - 1.16) | -7.00 (-7.46 to -6.54) |
| Timor-Leste | 310 (52 - 570) | 34.03 (5.47 - 62.35) | 146 (22 - 273) | 9.84 (1.40 - 19.47) | -4.00 (-4.15 to -3.86) |
| Togo | 177 (21 - 388) | 21.75 (2.92 - 49.15) | 14 (2 - 32) | 1.35 (0.16 - 3.33) | -9.42 (-9.88 to -8.97) |
| Tokelau | 1,620 (236 - 2,976) | 50.32 (7.38 - 96.16) | 854 (111 - 1,673) | 16.56 (2.26 - 31.70) | -3.31 (-3.59 to -3.03) |
| Tonga | 0 (0 - 0) | 6.67 (0.89 - 14.49) | 0 (0 - 0) | 2.37 (0.24 - 5.26) | -4.61 (-5.42 to -3.78) |
| Trinidad and Tobago | 1 (0 - 2) | 1.27 (0.16 - 2.95) | 0 (0 - 1) | 0.56 (0.07 - 1.31) | -2.15 (-2.68 to -1.62) |
| Tunisia | 8 (1 - 17) | 0.88 (0.10 - 1.86) | 2 (0 - 4) | 0.12 (0.01 - 0.27) | -7.05 (-7.69 to -6.40) |
| Turkey | 35 (4 - 84) | 0.45 (0.05 - 1.04) | 5 (1 - 14) | 0.05 (0.00 - 0.13) | -6.42 (-6.92 to -5.91) |
| Turkmenistan | 670 (71 - 1,520) | 1.07 (0.11 - 2.36) | 39 (5 - 100) | 0.06 (0.01 - 0.15) | -9.18 (-9.48 to -8.89) |
| Tuvalu | 38 (3 - 87) | 0.65 (0.06 - 1.51) | 1 (0 - 1) | 0.01 (0.00 - 0.03) | -14.75 (-15.66 to -13.83) |
| Uganda | 0 (0 - 1) | 8.24 (1.07 - 18.25) | 0 (0 - 0) | 2.18 (0.25 - 5.21) | -3.73 (-4.40 to -3.06) |
| Ukraine | 6,937 (1,013 - 14,839) | 44.41 (6.86 - 104.60) | 2,671 (347 - 5,433) | 11.43 (1.40 - 24.27) | -4.63 (-4.80 to -4.46) |
| United Arab Emirates | 3 (0 - 6) | 0.00 (0.00 - 0.01) | 17 (2 - 42) | 0.01 (0.00 - 0.03) | 3.99 (1.07 - 6.99) |
| United Kingdom | 12 (1 - 27) | 0.03 (0.00 - 0.07) | 1 (0 - 3) | 0.00 (0.00 - 0.01) | -9.39 (-10.50 to -8.27) |
| United Republic of Tanzania | 2 (0 - 4) | 0.15 (0.02 - 0.35) | 1 (0 - 2) | 0.04 (0.00 - 0.10) | -2.43 (-2.97 to -1.89) |
| United States of America | 4 (0 - 10) | 0.14 (0.01 - 0.30) | 2 (0 - 6) | 0.04 (0.00 - 0.09) | -3.36 (-3.76 to -2.96) |
| United States Virgin Islands | 7 (1 - 17) | 0.00 (0.00 - 0.01) | 71 (7 - 166) | 0.01 (0.00 - 0.03) | 6.62 (4.84 - 8.44) |
| Uruguay | 374 (44 - 788) | 1.15 (0.14 - 2.42) | 5 (0 - 11) | 0.01 (0.00 - 0.03) | -15.39 (-16.08 to -14.70) |
| Uzbekistan | 9 (1 - 17) | 13.40 (1.67 - 28.61) | 6 (1 - 12) | 4.28 (0.48 - 9.58) | -3.64 (-3.79 to -3.48) |
| Vanuatu | 338 (41 - 743) | 1.80 (0.22 - 3.97) | 79 (8 - 191) | 0.31 (0.03 - 0.75) | -5.99 (-6.77 to -5.21) |
| Venezuela (Bolivarian Republic of) | 918 (100 - 2,295) | 1.95 (0.23 - 5.07) | 79 (6 - 219) | 0.10 (0.01 - 0.29) | -9.33 (-9.53 to -9.14) |
| Viet Nam | 0 (0 - 0) | 0.22 (0.02 - 0.52) | 0 (0 - 0) | 0.06 (0.01 - 0.15) | -3.74 (-3.97 to -3.51) |
| Yemen | 3,877 (629 - 7,322) | 17.36 (2.67 - 34.07) | 294 (35 - 714) | 1.05 (0.12 - 2.53) | -9.08 (-9.48 to -8.69) |
| Zambia | 4,015 (588 - 7,576) | 55.38 (8.49 - 106.08) | 1,380 (193 - 2,697) | 12.49 (1.79 - 24.51) | -4.90 (-5.43 to -4.36) |
| Zimbabwe | 869 (107 - 1,741) | 12.73 (1.48 - 27.62) | 1,037 (121 - 2,250) | 10.76 (1.22 - 24.05) | 0.60 (-0.07 - 1.28) |

| Table S3. The YLLs and age-standardized YLLs Rate of no access to handwashing facility in 1990 and 2021, with Temporal Trends from 1990 to 2021 in 204 countries or territories. | | | | | |
| --- | --- | --- | --- | --- | --- |
| **location** | **Num_1990** | **YLLs_1990** | **Num_2021** | **YLLs_2021** | **EAPC_CI** |
| Afghanistan | 160,805 (21,656 - 301,959) | 857.35 (115.22 - 1638.33) | 78,191 (11,478 - 160,827) | 141.03 (20.96 - 291.34) | -6.10 (-6.84 to -5.37) |
| Albania | 567 (56 - 1,313) | 14.68 (1.44 - 33.94) | 25 (3 - 60) | 1.55 (0.17 - 3.78) | -7.93 (-8.63 to -7.22) |
| Algeria | 9,937 (1,171 - 24,617) | 28.96 (3.42 - 70.39) | 1,424 (151 - 3,336) | 3.41 (0.36 - 7.96) | -6.33 (-6.44 to -6.23) |
| American Samoa | 13 (1 - 30) | 36.66 (3.51 - 83.52) | 10 (1 - 23) | 25.07 (2.46 - 58.23) | -0.98 (-1.50 to -0.47) |
| Andorra | 0 (0 - 0) | 0.06 (0.01 - 0.14) | 0 (0 - 0) | 0.02 (0.00 - 0.06) | -2.30 (-2.47 to -2.13) |
| Angola | 607,940 (98,018 - 1,130,789) | 3768.24 (583.51 - 7001.27) | 121,534 (16,413 - 239,174) | 389.86 (51.86 - 764.75) | -7.17 (-7.71 to -6.63) |
| Antigua and Barbuda | 13 (2 - 28) | 23.06 (2.73 - 47.88) | 3 (0 - 6) | 4.14 (0.46 - 8.85) | -5.51 (-6.23 to -4.78) |
| Argentina | 3,893 (390 - 8,656) | 11.76 (1.18 - 26.16) | 294 (32 - 698) | 0.72 (0.08 - 1.70) | -7.99 (-8.59 to -7.38) |
| Armenia | 2,750 (337 - 5,858) | 74.62 (9.15 - 158.92) | 15 (1 - 35) | 0.78 (0.08 - 1.87) | -16.62 (-17.57 to -15.66) |
| Australia | 42 (4 - 98) | 0.27 (0.03 - 0.63) | 32 (3 - 80) | 0.09 (0.01 - 0.21) | -1.77 (-2.80 to -0.73) |
| Austria | 12 (1 - 27) | 0.17 (0.02 - 0.39) | 33 (3 - 76) | 0.18 (0.02 - 0.42) | 2.17 (1.30 - 3.05) |
| Azerbaijan | 9,276 (1,065 - 21,349) | 106.03 (12.18 - 243.65) | 597 (63 - 1,486) | 8.72 (0.91 - 21.88) | -9.05 (-9.62 to -8.47) |
| Bahamas | 37 (5 - 82) | 15.49 (2.05 - 34.44) | 8 (1 - 18) | 2.65 (0.29 - 6.29) | -6.22 (-7.06 to -5.37) |
| Bahrain | 53 (7 - 117) | 9.77 (1.18 - 21.45) | 12 (1 - 30) | 1.39 (0.12 - 3.42) | -5.93 (-6.14 to -5.72) |
| Bangladesh | 1,428,588 (217,743 - 2,800,449) | 1074.99 (160.29 - 2021.69) | 165,656 (22,938 - 410,510) | 121.74 (16.50 - 299.27) | -6.93 (-7.08 to -6.78) |
| Barbados | 23 (3 - 49) | 9.83 (1.08 - 21.54) | 6 (1 - 14) | 2.17 (0.23 - 5.04) | -4.86 (-5.51 to -4.20) |
| Belarus | 201 (21 - 468) | 2.64 (0.28 - 6.15) | 10 (1 - 25) | 0.14 (0.01 - 0.35) | -11.74 (-12.87 to -10.59) |
| Belgium | 64 (7 - 144) | 0.63 (0.07 - 1.43) | 230 (24 - 535) | 1.06 (0.11 - 2.45) | 2.77 (1.96 - 3.60) |
| Belize | 516 (71 - 979) | 187.44 (25.77 - 355.04) | 78 (9 - 159) | 21.52 (2.52 - 43.55) | -6.66 (-7.20 to -6.12) |
| Benin | 161,754 (26,508 - 292,288) | 2325.43 (364.92 - 4179.15) | 75,617 (10,118 - 164,223) | 531.89 (69.44 - 1083.02) | -4.62 (-4.78 to -4.47) |
| Bermuda | 3 (0 - 7) | 6.27 (0.71 - 13.59) | 1 (0 - 1) | 0.73 (0.08 - 1.70) | -7.00 (-7.85 to -6.14) |
| Bhutan | 13,077 (1,558 - 30,060) | 1830.08 (215.10 - 4268.31) | 347 (37 - 970) | 57.68 (6.20 - 159.87) | -11.59 (-11.90 to -11.27) |
| Bolivia (Plurinational State of) | 31,557 (3,917 - 66,854) | 330.14 (40.78 - 692.74) | 2,931 (332 - 6,744) | 26.11 (2.93 - 59.85) | -8.33 (-8.49 to -8.17) |
| Bosnia and Herzegovina | 94 (10 - 211) | 2.75 (0.29 - 6.20) | 17 (1 - 48) | 0.69 (0.07 - 1.72) | -4.21 (-4.77 to -3.65) |
| Botswana | 20,230 (2,908 - 36,775) | 1322.46 (176.93 - 2542.03) | 8,468 (1,135 - 16,823) | 388.58 (51.88 - 773.70) | -3.79 (-4.11 to -3.46) |
| Brazil | 460,446 (62,335 - 887,499) | 304.83 (41.25 - 586.63) | 11,927 (1,127 - 25,999) | 5.95 (0.56 - 13.10) | -12.33 (-12.53 to -12.13) |
| Brunei Darussalam | 1 (0 - 3) | 0.62 (0.07 - 1.51) | 1 (0 - 2) | 0.31 (0.03 - 0.80) | -1.52 (-1.76 to -1.28) |
| Bulgaria | 82 (9 - 191) | 1.53 (0.16 - 3.55) | 53 (5 - 129) | 1.03 (0.09 - 2.45) | -1.16 (-2.52 - 0.21) |
| Burkina Faso | 446,923 (61,320 - 803,250) | 2919.42 (417.34 - 5322.55) | 240,194 (39,047 - 442,702) | 790.08 (123.10 - 1406.75) | -4.20 (-4.37 to -4.03) |
| Burundi | 148,338 (20,535 - 281,511) | 1899.82 (273.34 - 3548.26) | 89,353 (11,458 - 189,635) | 709.61 (86.63 - 1403.90) | -2.98 (-3.21 to -2.76) |
| Cabo Verde | 108,439 (14,325 - 225,357) | 743.09 (98.14 - 1522.08) | 5,284 (559 - 12,036) | 37.09 (3.96 - 83.98) | -10.31 (-10.78 to -9.84) |
| Cambodia | 229,007 (34,185 - 451,672) | 1472.47 (225.88 - 2858.67) | 139,813 (16,549 - 317,421) | 394.79 (49.09 - 829.62) | -3.91 (-4.44 to -3.37) |
| Cameroon | 15 (2 - 36) | 0.06 (0.01 - 0.14) | 113 (12 - 274) | 0.19 (0.02 - 0.46) | 5.87 (3.92 - 7.85) |
| Canada | 4,985 (721 - 8,957) | 982.00 (141.99 - 1772.68) | 478 (73 - 947) | 104.53 (16.12 - 209.26) | -7.52 (-8.14 to -6.90) |
| Central African Republic | 113,433 (18,740 - 206,902) | 2656.80 (423.98 - 4882.91) | 86,042 (11,626 - 173,701) | 1382.18 (183.96 - 2809.33) | -1.78 (-1.93 to -1.63) |
| Chad | 466,113 (71,940 - 874,358) | 4536.89 (688.48 - 8454.91) | 619,393 (90,668 - 1,249,227) | 2171.73 (304.10 - 4195.43) | -2.28 (-2.46 to -2.11) |
| Chile | 609 (68 - 1,411) | 4.67 (0.52 - 10.82) | 95 (10 - 232) | 0.50 (0.05 - 1.24) | -5.93 (-6.63 to -5.24) |
| China | 870,366 (100,072 - 1,780,837) | 79.94 (9.18 - 163.82) | 5,875 (583 - 12,689) | 0.67 (0.07 - 1.43) | -15.90 (-16.43 to -15.36) |
| Colombia | 29,693 (3,616 - 62,432) | 74.26 (9.05 - 156.56) | 1,897 (223 - 4,422) | 4.61 (0.54 - 10.87) | -9.63 (-10.27 to -8.98) |
| Comoros | 7,320 (1,058 - 14,317) | 1099.42 (156.48 - 2105.72) | 1,994 (237 - 4,030) | 293.22 (35.34 - 586.28) | -4.19 (-4.49 to -3.89) |
| Congo | 43,357 (5,712 - 85,831) | 1345.28 (186.35 - 2658.82) | 10,975 (1,234 - 25,888) | 240.12 (27.67 - 552.08) | -5.31 (-5.78 to -4.83) |
| Cook Islands | 1 (0 - 2) | 7.38 (0.85 - 17.42) | 1 (0 - 2) | 3.08 (0.31 - 7.61) | -2.93 (-3.46 to -2.40) |
| Costa Rica | 671 (75 - 1,491) | 20.41 (2.28 - 45.98) | 87 (9 - 200) | 2.06 (0.21 - 4.86) | -7.39 (-8.04 to -6.74) |
| Côte d'Ivoire | 20 (2 - 50) | 0.64 (0.07 - 1.56) | 25 (3 - 60) | 0.62 (0.07 - 1.51) | 1.22 (-0.73 - 3.20) |
| Croatia | 2,005 (255 - 4,200) | 21.35 (2.72 - 44.82) | 362 (39 - 859) | 2.97 (0.31 - 6.97) | -6.56 (-7.29 to -5.83) |
| Cuba | 19 (2 - 47) | 3.14 (0.32 - 7.82) | 12 (1 - 31) | 0.79 (0.08 - 2.00) | -4.31 (-4.59 to -4.03) |
| Cyprus | 97 (12 - 238) | 1.36 (0.16 - 3.34) | 151 (16 - 364) | 1.33 (0.14 - 3.24) | 1.39 (-0.81 - 3.64) |
| Czechia | 254,744 (37,043 - 464,019) | 1387.44 (199.53 - 2579.60) | 112,584 (14,172 - 231,945) | 359.26 (45.27 - 697.18) | -3.79 (-4.13 to -3.44) |
| Democratic People's Republic of Korea | 33 (3 - 80) | 0.59 (0.05 - 1.41) | 94 (9 - 239) | 0.86 (0.08 - 2.20) | 2.48 (1.72 - 3.24) |
| Democratic Republic of the Congo | 9,188 (1,457 - 17,082) | 1711.28 (263.74 - 3179.11) | 3,213 (395 - 6,428) | 299.42 (35.67 - 580.87) | -5.56 (-5.82 to -5.29) |
| Denmark | 28 (3 - 57) | 35.25 (3.67 - 72.86) | 6 (1 - 14) | 13.50 (1.51 - 32.23) | -2.93 (-3.54 to -2.31) |
| Djibouti | 38,865 (5,701 - 75,841) | 392.48 (57.06 - 764.66) | 4,956 (645 - 10,804) | 48.18 (6.28 - 104.94) | -6.53 (-6.94 to -6.11) |
| Dominica | 1,013,678 (153,498 - 1,822,878) | 1673.52 (252.96 - 3042.61) | 287,062 (38,011 - 592,828) | 334.37 (42.52 - 687.98) | -5.03 (-6.05 to -4.00) |
| Dominican Republic | 21,659 (2,813 - 45,523) | 174.99 (22.67 - 367.72) | 658 (79 - 1,502) | 4.06 (0.48 - 9.27) | -12.04 (-12.46 to -11.62) |
| Ecuador | 292,885 (35,971 - 626,239) | 344.17 (42.38 - 735.66) | 12,976 (1,246 - 32,011) | 11.12 (1.09 - 26.92) | -10.45 (-10.90 to -10.00) |
| Egypt | 25,408 (3,015 - 52,240) | 346.24 (40.57 - 705.85) | 724 (70 - 1,766) | 11.90 (1.14 - 28.94) | -10.86 (-11.59 to -10.13) |
| El Salvador | 21,696 (3,204 - 41,366) | 3205.43 (485.63 - 6232.12) | 1,352 (164 - 2,973) | 114.60 (12.89 - 252.26) | -11.88 (-12.41 to -11.35) |
| Equatorial Guinea | 146,230 (20,499 - 259,994) | 3282.52 (474.40 - 6229.41) | 43,281 (5,418 - 91,035) | 735.81 (87.51 - 1645.52) | -4.78 (-4.90 to -4.67) |
| Eritrea | 20 (2 - 48) | 1.66 (0.19 - 4.07) | 1 (0 - 2) | 0.07 (0.01 - 0.16) | -11.88 (-12.80 to -10.95) |
| Estonia | 21,878 (3,311 - 40,717) | 1981.46 (297.84 - 3735.46) | 5,779 (851 - 11,843) | 498.59 (72.88 - 1025.83) | -4.26 (-4.77 to -3.75) |
| Eswatini | 1,931,364 (289,507 - 3,907,030) | 2914.65 (492.00 - 5876.32) | 629,938 (103,797 - 1,103,867) | 624.85 (96.17 - 1115.44) | -5.23 (-5.40 to -5.06) |
| Ethiopia | 157 (19 - 332) | 169.19 (20.08 - 359.47) | 35 (4 - 79) | 51.25 (5.57 - 119.73) | -3.59 (-4.55 to -2.62) |
| Fiji | 723 (82 - 1,513) | 135.39 (15.47 - 284.99) | 403 (44 - 922) | 56.68 (6.17 - 131.52) | -2.31 (-2.65 to -1.97) |
| Finland | 14 (1 - 34) | 0.24 (0.02 - 0.59) | 26 (3 - 64) | 0.24 (0.03 - 0.58) | 0.99 (0.21 - 1.77) |
| France | 485 (52 - 1,089) | 0.76 (0.08 - 1.70) | 365 (38 - 844) | 0.36 (0.04 - 0.81) | -2.47 (-3.17 to -1.76) |
| Gabon | 11,873 (1,515 - 25,136) | 949.44 (130.77 - 1961.15) | 1,697 (196 - 4,131) | 108.75 (12.41 - 260.60) | -6.76 (-7.01 to -6.50) |
| Gambia | 837 (83 - 1,927) | 19.16 (1.90 - 44.18) | 8 (1 - 19) | 0.29 (0.03 - 0.68) | -14.82 (-15.71 to -13.92) |
| Georgia | 272 (27 - 653) | 0.33 (0.03 - 0.79) | 749 (83 - 1,737) | 0.43 (0.05 - 0.97) | 3.24 (2.11 - 4.38) |
| Germany | 257,750 (42,290 - 489,059) | 1348.25 (230.07 - 2545.09) | 78,251 (11,434 - 146,981) | 265.70 (38.95 - 496.10) | -5.02 (-5.20 to -4.83) |
| Ghana | 5 (0 - 11) | 0.05 (0.00 - 0.12) | 22 (2 - 52) | 0.10 (0.01 - 0.23) | 3.40 (2.80 - 4.01) |
| Greece | 0 (0 - 1) | 0.58 (0.06 - 1.48) | 0 (0 - 0) | 0.27 (0.03 - 0.75) | -2.21 (-2.33 to -2.09) |
| Greenland | 36 (5 - 72) | 34.73 (4.34 - 69.06) | 3 (0 - 7) | 3.73 (0.44 - 8.27) | -6.78 (-7.66 to -5.89) |
| Grenada | 9 (1 - 21) | 9.75 (1.02 - 22.55) | 9 (1 - 20) | 5.00 (0.56 - 11.76) | -1.87 (-2.69 to -1.04) |
| Guam | 115,491 (15,426 - 225,768) | 1035.46 (138.05 - 2002.08) | 11,583 (1,472 - 24,984) | 82.48 (10.46 - 176.90) | -7.55 (-7.89 to -7.21) |
| Guatemala | 270,096 (44,925 - 509,877) | 3110.94 (498.80 - 5956.44) | 64,113 (8,185 - 140,967) | 510.34 (64.74 - 1062.26) | -5.61 (-5.82 to -5.39) |
| Guinea | 38,140 (6,189 - 70,910) | 2738.49 (430.66 - 5152.92) | 9,970 (1,423 - 19,319) | 543.36 (71.41 - 1035.92) | -5.28 (-5.83 to -4.72) |
| Guinea-Bissau | 3,318 (427 - 6,625) | 320.79 (41.26 - 633.89) | 329 (45 - 704) | 46.69 (6.41 - 99.54) | -5.99 (-6.24 to -5.73) |
| Guyana | 239,378 (34,197 - 426,790) | 2271.17 (320.30 - 4053.40) | 91,025 (13,755 - 169,855) | 608.24 (90.52 - 1135.06) | -3.99 (-4.47 to -3.51) |
| Haiti | 24,884 (2,958 - 49,889) | 367.44 (43.57 - 746.98) | 2,925 (294 - 7,298) | 33.46 (3.37 - 85.31) | -7.59 (-7.84 to -7.33) |
| Honduras | 99 (11 - 232) | 1.46 (0.16 - 3.38) | 132 (12 - 341) | 1.51 (0.13 - 3.87) | 1.62 (-0.44 - 3.72) |
| Hungary | 0 (0 - 1) | 0.17 (0.02 - 0.38) | 1 (0 - 3) | 0.22 (0.02 - 0.52) | 2.36 (1.46 - 3.28) |
| Iceland | 16,142,776 (2,436,552 - 29,304,824) | 2013.25 (300.95 - 3745.95) | 2,149,516 (257,494 - 4,623,207) | 191.73 (22.88 - 409.81) | -7.33 (-7.65 to -7.01) |
| India | 1,609,964 (193,154 - 3,444,789) | 908.84 (110.41 - 2007.91) | 122,978 (12,042 - 289,719) | 58.76 (5.51 - 138.73) | -8.17 (-8.39 to -7.95) |
| Indonesia | 34,397 (3,217 - 81,892) | 46.60 (4.47 - 109.25) | 860 (86 - 2,152) | 1.31 (0.13 - 3.18) | -10.26 (-10.46 to -10.06) |
| Iran (Islamic Republic of) | 17,169 (1,710 - 40,837) | 56.34 (5.64 - 131.18) | 1,680 (162 - 3,929) | 4.33 (0.42 - 10.15) | -8.60 (-8.87 to -8.33) |
| Iraq | 5 (0 - 13) | 0.16 (0.01 - 0.38) | 12 (1 - 28) | 0.17 (0.02 - 0.41) | 1.40 (0.60 - 2.20) |
| Ireland | 37 (4 - 88) | 0.77 (0.09 - 1.83) | 110 (11 - 253) | 0.90 (0.09 - 2.06) | 1.83 (1.03 - 2.63) |
| Israel | 64 (6 - 153) | 0.13 (0.01 - 0.32) | 546 (68 - 1,257) | 0.46 (0.06 - 1.04) | 7.90 (5.95 - 9.89) |
| Italy | 2,423 (316 - 4,912) | 93.25 (12.13 - 188.84) | 233 (25 - 514) | 9.76 (1.05 - 21.55) | -6.18 (-7.42 to -4.93) |
| Jamaica | 285 (30 - 670) | 0.27 (0.03 - 0.64) | 456 (41 - 1,077) | 0.17 (0.02 - 0.40) | -1.04 (-1.53 to -0.56) |
| Japan | 727 (86 - 1,619) | 12.84 (1.50 - 28.90) | 201 (22 - 465) | 2.00 (0.22 - 4.60) | -6.08 (-6.22 to -5.94) |
| Jordan | 4,014 (404 - 9,111) | 22.18 (2.23 - 50.37) | 38 (4 - 93) | 0.20 (0.02 - 0.47) | -16.81 (-17.70 to -15.92) |
| Kazakhstan | 546,491 (79,165 - 1,038,004) | 1691.19 (251.89 - 3170.00) | 200,388 (29,960 - 361,792) | 456.15 (66.45 - 837.07) | -3.72 (-4.10 to -3.35) |
| Kenya | 726 (99 - 1,491) | 975.51 (131.76 - 2030.58) | 265 (31 - 584) | 309.54 (36.12 - 682.54) | -3.39 (-3.65 to -3.14) |
| Kiribati | 46 (5 - 101) | 2.71 (0.32 - 5.96) | 9 (1 - 20) | 0.33 (0.04 - 0.76) | -4.47 (-5.47 to -3.45) |
| Kuwait | 4,193 (490 - 9,916) | 67.20 (7.86 - 158.98) | 230 (26 - 551) | 3.02 (0.34 - 7.21) | -10.72 (-11.54 to -9.90) |
| Kyrgyzstan | 113,275 (15,826 - 226,582) | 1765.47 (248.81 - 3687.25) | 7,605 (909 - 15,833) | 106.26 (12.80 - 227.67) | -9.05 (-9.33 to -8.77) |
| Lao People's Democratic Republic | 31 (3 - 73) | 1.54 (0.14 - 3.67) | 1 (0 - 3) | 0.08 (0.01 - 0.18) | -11.54 (-12.43 to -10.64) |
| Latvia | 859 (111 - 2,003) | 23.21 (3.00 - 53.56) | 164 (15 - 404) | 3.56 (0.33 - 8.84) | -6.00 (-6.24 to -5.76) |
| Lebanon | 51,608 (8,523 - 91,401) | 2775.29 (446.32 - 4979.98) | 25,612 (4,048 - 46,521) | 1449.84 (220.95 - 2715.58) | -1.89 (-2.16 to -1.62) |
| Lesotho | 131,836 (20,161 - 228,551) | 3101.38 (484.82 - 5330.98) | 33,918 (4,822 - 72,541) | 584.37 (80.37 - 1151.68) | -5.74 (-6.05 to -5.43) |
| Liberia | 3,551 (428 - 8,113) | 61.33 (7.32 - 136.61) | 164 (17 - 420) | 3.68 (0.36 - 9.57) | -8.28 (-9.42 to -7.12) |
| Libya | 73 (8 - 166) | 2.51 (0.28 - 5.71) | 4 (0 - 11) | 0.16 (0.02 - 0.40) | -10.62 (-11.55 to -9.69) |
| Lithuania | 2 (0 - 4) | 0.42 (0.05 - 0.97) | 5 (0 - 12) | 0.53 (0.05 - 1.30) | 1.79 (1.08 - 2.52) |
| Luxembourg | 472,261 (73,857 - 824,730) | 2526.40 (398.21 - 4426.24) | 314,734 (41,695 - 620,432) | 978.86 (126.45 - 1864.13) | -2.56 (-2.81 to -2.31) |
| Madagascar | 516,905 (69,349 - 939,934) | 3273.91 (470.61 - 5985.96) | 117,073 (16,722 - 215,020) | 695.41 (96.09 - 1251.69) | -5.06 (-5.16 to -4.96) |
| Malawi | 2,099 (226 - 5,176) | 12.32 (1.29 - 30.61) | 588 (57 - 1,442) | 2.27 (0.22 - 5.57) | -5.08 (-5.74 to -4.41) |
| Malaysia | 1,065 (129 - 2,572) | 306.18 (36.19 - 739.75) | 16 (2 - 38) | 4.80 (0.50 - 11.70) | -12.83 (-13.67 to -11.99) |
| Maldives | 358,038 (60,276 - 682,873) | 2772.22 (441.72 - 5221.13) | 154,004 (22,783 - 287,009) | 594.47 (79.65 - 1192.95) | -4.81 (-5.05 to -4.56) |
| Mali | 0 (0 - 1) | 0.11 (0.01 - 0.25) | 1 (0 - 3) | 0.16 (0.02 - 0.38) | 2.14 (1.32 - 2.96) |
| Malta | 69 (9 - 144) | 192.21 (23.43 - 418.19) | 26 (3 - 59) | 70.05 (8.56 - 155.65) | -2.88 (-3.44 to -2.31) |
| Marshall Islands | 41,296 (6,786 - 80,520) | 1433.24 (235.48 - 2737.99) | 11,170 (1,379 - 24,069) | 244.26 (31.29 - 487.87) | -5.66 (-6.08 to -5.23) |
| Mauritania | 83 (8 - 205) | 8.56 (0.86 - 21.03) | 19 (2 - 44) | 1.61 (0.16 - 3.83) | -3.59 (-4.12 to -3.06) |
| Mauritius | 198,090 (26,772 - 432,082) | 186.93 (25.10 - 407.44) | 8,552 (915 - 18,775) | 7.80 (0.83 - 17.12) | -9.62 (-10.24 to -8.98) |
| Mexico | 917 (101 - 1,929) | 22.89 (2.53 - 48.31) | 27 (3 - 63) | 1.32 (0.14 - 3.16) | -10.15 (-10.88 to -9.42) |
| Micronesia (Federated States of) | 0 (0 - 0) | 0.08 (0.01 - 0.19) | 0 (0 - 0) | 0.08 (0.01 - 0.21) | -0.30 (-0.57 to -0.02) |
| Monaco | 2,832 (372 - 6,576) | 83.42 (10.93 - 192.79) | 242 (21 - 690) | 6.44 (0.55 - 18.39) | -8.35 (-8.56 to -8.13) |
| Mongolia | 1 (0 - 4) | 0.29 (0.03 - 0.73) | 0 (0 - 1) | 0.06 (0.01 - 0.15) | -5.21 (-5.59 to -4.82) |
| Montenegro | 168,262 (23,180 - 350,152) | 465.84 (64.06 - 970.70) | 5,047 (533 - 12,821) | 15.98 (1.69 - 40.56) | -10.28 (-10.54 to -10.02) |
| Morocco | 470,712 (74,035 - 879,728) | 2325.79 (366.30 - 4467.12) | 160,486 (23,074 - 327,539) | 506.90 (63.92 - 1053.34) | -4.72 (-4.91 to -4.53) |
| Mozambique | 402,684 (50,336 - 948,145) | 876.71 (106.64 - 2044.85) | 21,776 (2,148 - 48,275) | 43.71 (4.33 - 96.20) | -9.73 (-9.93 to -9.53) |
| Myanmar | 24,956 (3,452 - 47,941) | 1501.23 (197.27 - 2938.61) | 9,260 (1,231 - 19,490) | 396.55 (52.41 - 830.34) | -4.09 (-4.35 to -3.82) |
| Namibia | 9 (1 - 18) | 91.97 (10.14 - 194.95) | 3 (0 - 7) | 40.69 (4.61 - 96.47) | -2.40 (-2.81 to -1.99) |
| Nauru | 361,582 (56,512 - 651,770) | 1388.06 (209.02 - 2592.22) | 22,237 (3,157 - 47,617) | 87.11 (12.21 - 189.13) | -8.82 (-9.08 to -8.56) |
| Nepal | 27 (3 - 64) | 0.19 (0.02 - 0.44) | 112 (12 - 271) | 0.38 (0.04 - 0.89) | 3.26 (2.44 - 4.08) |
| Netherlands | 13 (1 - 29) | 0.39 (0.04 - 0.89) | 16 (2 - 38) | 0.24 (0.02 - 0.59) | 1.88 (0.09 - 3.71) |
| New Zealand | 28,265 (3,665 - 57,058) | 451.70 (58.22 - 915.26) | 1,046 (118 - 2,444) | 17.32 (1.95 - 40.16) | -10.22 (-10.62 to -9.82) |
| Nicaragua | 693,252 (109,461 - 1,233,414) | 5012.90 (789.33 - 8920.19) | 346,895 (49,100 - 674,845) | 1081.18 (148.48 - 2109.33) | -5.26 (-5.61 to -4.91) |
| Niger | 4,705,836 (712,461 - 8,434,348) | 3223.45 (497.65 - 5781.93) | 2,797,643 (388,040 - 5,123,748) | 872.09 (121.94 - 1562.68) | -4.06 (-4.47 to -3.65) |
| Nigeria | 1 (0 - 3) | 49.39 (5.72 - 109.25) | 0 (0 - 1) | 33.27 (4.04 - 82.13) | -2.75 (-3.55 to -1.94) |
| Niue | 654 (60 - 1,612) | 2.81 (0.26 - 6.94) | 136 (13 - 352) | 0.67 (0.07 - 1.67) | -4.22 (-4.43 to -4.02) |
| North Macedonia | 410 (42 - 906) | 24.86 (2.56 - 55.01) | 22 (2 - 55) | 1.65 (0.15 - 4.21) | -7.55 (-8.54 to -6.54) |
| Northern Mariana Islands | 3 (0 - 8) | 14.98 (1.74 - 35.81) | 5 (0 - 12) | 12.81 (1.24 - 30.47) | 0.18 (-0.44 - 0.80) |
| Norway | 14 (1 - 34) | 0.20 (0.02 - 0.51) | 75 (9 - 178) | 0.66 (0.08 - 1.56) | 5.14 (3.99 - 6.31) |
| Oman | 1,062 (123 - 2,548) | 36.88 (4.18 - 86.81) | 74 (8 - 189) | 2.15 (0.25 - 5.45) | -8.61 (-9.38 to -7.83) |
| Pakistan | 1,740,500 (238,989 - 3,324,714) | 1152.93 (155.24 - 2251.10) | 296,941 (34,431 - 606,742) | 135.16 (15.65 - 283.36) | -6.26 (-6.62 to -5.90) |
| Palau | 9 (1 - 21) | 78.13 (8.54 - 190.23) | 5 (1 - 13) | 36.88 (4.04 - 92.57) | -2.03 (-2.57 to -1.48) |
| Palestine | 2,323 (285 - 5,045) | 61.72 (7.47 - 131.59) | 124 (13 - 286) | 2.37 (0.26 - 5.45) | -9.64 (-9.95 to -9.33) |
| Panama | 1,710 (171 - 3,805) | 66.47 (6.64 - 146.81) | 325 (35 - 729) | 8.68 (0.94 - 19.47) | -5.44 (-5.89 to -4.98) |
| Papua New Guinea | 44,519 (5,801 - 82,990) | 929.32 (117.49 - 1717.43) | 42,522 (5,868 - 87,785) | 379.30 (50.01 - 760.89) | -2.39 (-2.60 to -2.18) |
| Paraguay | 9,571 (1,211 - 19,461) | 171.57 (21.38 - 348.01) | 500 (57 - 1,242) | 8.04 (0.91 - 19.87) | -10.44 (-10.86 to -10.01) |
| Peru | 50,331 (5,827 - 104,237) | 180.35 (21.30 - 377.73) | 2,402 (231 - 5,569) | 7.16 (0.69 - 16.65) | -10.67 (-11.19 to -10.15) |
| Philippines | 184,036 (24,267 - 389,926) | 223.75 (30.22 - 478.56) | 23,780 (2,748 - 52,723) | 23.38 (2.68 - 52.67) | -6.69 (-6.85 to -6.52) |
| Poland | 222 (24 - 518) | 0.79 (0.09 - 1.84) | 310 (32 - 753) | 0.72 (0.07 - 1.76) | 1.86 (-0.64 - 4.43) |
| Portugal | 74 (7 - 171) | 1.07 (0.11 - 2.51) | 101 (10 - 244) | 0.44 (0.04 - 1.06) | -1.61 (-2.24 to -0.96) |
| Puerto Rico | 224 (25 - 470) | 6.71 (0.76 - 14.08) | 94 (10 - 200) | 2.61 (0.28 - 5.75) | -4.09 (-5.29 to -2.88) |
| Qatar | 16 (1 - 36) | 3.59 (0.33 - 8.10) | 9 (1 - 20) | 0.58 (0.05 - 1.39) | -5.49 (-5.60 to -5.38) |
| Republic of Korea | 1,649 (181 - 3,695) | 10.44 (1.14 - 23.42) | 227 (22 - 514) | 1.36 (0.12 - 3.09) | -6.88 (-8.14 to -5.61) |
| Republic of Moldova | 4,728 (577 - 11,021) | 4.36 (0.53 - 10.17) | 334 (38 - 835) | 0.30 (0.03 - 0.74) | -9.94 (-10.60 to -9.28) |
| Romania | 198,428 (29,735 - 360,561) | 1782.30 (270.49 - 3293.63) | 47,843 (7,428 - 91,801) | 360.54 (53.40 - 707.12) | -5.98 (-6.61 to -5.34) |
| Russian Federation | 47 (6 - 97) | 110.47 (13.14 - 227.44) | 6 (1 - 14) | 15.00 (1.77 - 33.39) | -6.08 (-6.92 to -5.23) |
| Rwanda | 93 (11 - 183) | 63.53 (7.37 - 123.84) | 10 (1 - 23) | 7.11 (0.74 - 15.81) | -6.95 (-7.84 to -6.06) |
| Saint Kitts and Nevis | 144 (19 - 287) | 127.40 (16.73 - 249.92) | 13 (2 - 26) | 13.38 (1.55 - 28.07) | -6.88 (-7.67 to -6.09) |
| Saint Lucia | 29 (4 - 71) | 22.08 (2.67 - 53.06) | 13 (1 - 29) | 7.65 (0.77 - 17.76) | -3.50 (-3.92 to -3.07) |
| Saint Vincent and the Grenadines | 0 (0 - 0) | 0.12 (0.01 - 0.29) | 0 (0 - 0) | 0.04 (0.00 - 0.11) | -2.36 (-2.63 to -2.08) |
| Samoa | 11,106 (1,254 - 25,003) | 52.48 (5.96 - 118.97) | 482 (47 - 1,236) | 2.18 (0.21 - 5.68) | -10.07 (-10.40 to -9.73) |
| San Marino | 284,239 (44,242 - 516,437) | 2510.98 (385.76 - 4489.54) | 40,558 (5,308 - 80,288) | 271.34 (36.80 - 541.68) | -7.05 (-7.44 to -6.65) |
| Sao Tome and Principe | 142 (15 - 351) | 2.05 (0.22 - 5.11) | 27 (3 - 73) | 0.41 (0.04 - 1.07) | -4.98 (-5.51 to -4.45) |
| Saudi Arabia | 8 (1 - 20) | 11.34 (1.06 - 28.15) | 4 (0 - 10) | 4.06 (0.36 - 10.78) | -2.66 (-3.02 to -2.31) |
| Senegal | 166,893 (23,034 - 312,395) | 2532.75 (349.39 - 4732.54) | 57,162 (7,694 - 113,167) | 632.81 (87.80 - 1265.84) | -4.71 (-5.19 to -4.22) |
| Serbia | 18 (2 - 43) | 0.83 (0.08 - 1.96) | 10 (1 - 24) | 0.15 (0.02 - 0.38) | -4.16 (-4.65 to -3.66) |
| Seychelles | 27 (3 - 66) | 0.67 (0.07 - 1.59) | 12 (1 - 33) | 0.35 (0.03 - 0.93) | -1.51 (-2.18 to -0.83) |
| Sierra Leone | 3 (0 - 8) | 0.23 (0.03 - 0.57) | 5 (1 - 12) | 0.20 (0.02 - 0.50) | 0.59 (-1.47 - 2.69) |
| Singapore | 1,340 (192 - 2,633) | 392.45 (57.16 - 790.10) | 519 (65 - 1,042) | 98.47 (12.94 - 205.68) | -4.05 (-4.21 to -3.89) |
| Slovakia | 376,750 (59,901 - 730,117) | 3280.36 (516.80 - 6521.66) | 337,615 (52,367 - 647,062) | 1386.07 (206.62 - 2717.55) | -3.01 (-3.16 to -2.86) |
| Slovenia | 497,597 (73,690 - 904,053) | 1137.79 (168.61 - 2058.24) | 153,599 (19,180 - 297,151) | 312.20 (39.24 - 606.19) | -3.93 (-4.29 to -3.57) |
| Solomon Islands | 401 (41 - 1,045) | 1.44 (0.14 - 3.92) | 174 (17 - 441) | 0.27 (0.03 - 0.67) | -4.63 (-5.14 to -4.13) |
| Somalia | 297,974 (42,825 - 571,918) | 3443.30 (496.71 - 6621.47) | 243,226 (33,568 - 478,182) | 2058.25 (282.63 - 4148.18) | -1.43 (-1.64 to -1.23) |
| South Africa | 139 (15 - 332) | 0.40 (0.04 - 0.94) | 362 (42 - 847) | 0.41 (0.05 - 0.97) | 1.44 (0.87 - 2.02) |
| South Sudan | 14,585 (1,732 - 33,117) | 104.21 (12.50 - 238.78) | 775 (75 - 2,062) | 3.61 (0.37 - 9.29) | -10.06 (-10.62 to -9.49) |
| Spain | 248,692 (31,592 - 563,238) | 688.83 (87.94 - 1532.86) | 16,532 (1,460 - 53,289) | 32.80 (2.97 - 100.87) | -9.49 (-10.12 to -8.85) |
| Sri Lanka | 882 (105 - 1,776) | 213.70 (25.44 - 431.47) | 182 (20 - 388) | 37.76 (4.09 - 82.88) | -5.77 (-6.08 to -5.46) |
| Sudan | 9 (1 - 20) | 0.06 (0.01 - 0.14) | 176 (17 - 410) | 0.70 (0.07 - 1.61) | 9.36 (8.13 - 10.60) |
| Suriname | 21 (2 - 50) | 0.28 (0.03 - 0.67) | 54 (6 - 126) | 0.31 (0.03 - 0.78) | 1.74 (1.02 - 2.48) |
| Sweden | 7,108 (861 - 16,451) | 34.82 (4.22 - 79.62) | 195 (19 - 491) | 1.85 (0.18 - 4.64) | -8.57 (-9.40 to -7.74) |
| Switzerland | 2,606 (419 - 4,595) | 1435.83 (226.39 - 2533.19) | 193 (27 - 384) | 105.63 (14.31 - 209.45) | -8.45 (-8.89 to -8.01) |
| Syrian Arab Republic | 357 (38 - 830) | 2.17 (0.23 - 5.06) | 26 (3 - 61) | 0.11 (0.01 - 0.25) | -9.06 (-10.02 to -8.09) |
| Taiwan (Province of China) | 40,449 (5,327 - 83,412) | 421.06 (55.35 - 866.61) | 12,450 (1,540 - 28,115) | 92.96 (11.48 - 208.39) | -5.66 (-6.24 to -5.08) |
| Tajikistan | 676,589 (94,740 - 1,282,421) | 1856.65 (273.15 - 3441.46) | 245,361 (31,013 - 478,340) | 431.61 (54.72 - 849.15) | -4.64 (-4.86 to -4.42) |
| Thailand | 62,298 (5,730 - 175,251) | 143.31 (13.42 - 430.43) | 9,865 (993 - 24,097) | 13.84 (1.48 - 32.78) | -7.03 (-7.48 to -6.58) |
| Timor-Leste | 23,631 (3,911 - 44,038) | 1597.68 (264.32 - 2917.08) | 8,718 (1,367 - 15,922) | 358.24 (53.69 - 670.30) | -4.89 (-5.11 to -4.66) |
| Togo | 14,319 (1,726 - 31,223) | 1118.95 (135.02 - 2450.58) | 787 (84 - 1,812) | 51.48 (5.82 - 122.21) | -10.40 (-10.86 to -9.93) |
| Tokelau | 123,048 (17,905 - 228,843) | 2306.79 (335.05 - 4285.97) | 52,094 (6,859 - 109,061) | 619.95 (80.33 - 1224.04) | -3.89 (-4.23 to -3.54) |
| Tonga | 3 (0 - 6) | 178.56 (22.57 - 359.51) | 1 (0 - 3) | 120.45 (11.80 - 278.77) | -4.33 (-5.73 to -2.91) |
| Trinidad and Tobago | 30 (4 - 65) | 31.30 (4.04 - 70.50) | 11 (1 - 25) | 11.67 (1.40 - 25.46) | -2.62 (-3.11 to -2.12) |
| Tunisia | 420 (44 - 886) | 38.01 (3.98 - 80.51) | 58 (7 - 131) | 4.95 (0.58 - 11.41) | -7.22 (-7.79 to -6.65) |
| Turkey | 2,774 (308 - 6,608) | 27.95 (3.17 - 66.13) | 186 (19 - 482) | 1.93 (0.20 - 4.94) | -7.75 (-8.27 to -7.22) |
| Turkmenistan | 56,187 (5,921 - 128,609) | 82.08 (8.66 - 186.92) | 1,626 (201 - 3,964) | 2.80 (0.34 - 6.78) | -10.68 (-10.87 to -10.48) |
| Tuvalu | 3,310 (304 - 7,667) | 56.45 (5.19 - 130.76) | 47 (5 - 114) | 0.89 (0.09 - 2.14) | -14.96 (-15.88 to -14.03) |
| Uganda | 25 (3 - 52) | 236.62 (28.56 - 501.89) | 5 (1 - 11) | 47.10 (5.41 - 104.66) | -4.55 (-5.27 to -3.83) |
| Ukraine | 515,345 (74,819 - 1,100,995) | 1860.49 (276.49 - 4030.99) | 163,158 (21,402 - 339,389) | 376.22 (48.23 - 751.50) | -5.29 (-5.45 to -5.13) |
| United Arab Emirates | 50 (5 - 111) | 0.08 (0.01 - 0.17) | 236 (22 - 566) | 0.20 (0.02 - 0.48) | 2.86 (0.07 - 5.74) |
| United Kingdom | 901 (98 - 2,081) | 2.56 (0.28 - 5.87) | 53 (6 - 124) | 0.26 (0.03 - 0.59) | -9.77 (-10.85 to -8.68) |
| United Republic of Tanzania | 119 (13 - 277) | 6.42 (0.66 - 14.71) | 48 (6 - 124) | 1.21 (0.13 - 3.13) | -3.53 (-4.06 to -3.00) |
| United States of America | 200 (21 - 447) | 6.86 (0.71 - 15.46) | 43 (5 - 100) | 1.11 (0.13 - 2.66) | -5.51 (-5.85 to -5.16) |
| United States Virgin Islands | 264 (32 - 647) | 0.12 (0.01 - 0.29) | 1,204 (120 - 2,868) | 0.23 (0.02 - 0.56) | 3.33 (2.20 - 4.47) |
| Uruguay | 32,864 (3,866 - 69,232) | 98.44 (11.60 - 207.19) | 366 (36 - 835) | 0.98 (0.10 - 2.23) | -15.86 (-16.55 to -15.17) |
| Uzbekistan | 478 (64 - 1,008) | 334.05 (44.64 - 675.85) | 235 (26 - 517) | 98.56 (11.29 - 205.46) | -3.86 (-4.01 to -3.72) |
| Vanuatu | 25,780 (3,152 - 57,045) | 108.31 (13.27 - 238.71) | 3,450 (368 - 8,538) | 14.53 (1.55 - 36.15) | -6.89 (-7.76 to -6.00) |
| Venezuela (Bolivarian Republic of) | 46,263 (4,900 - 112,910) | 64.84 (6.73 - 160.96) | 1,920 (161 - 5,109) | 2.23 (0.19 - 6.02) | -10.61 (-10.86 to -10.37) |
| Viet Nam | 9 (1 - 20) | 9.14 (0.99 - 19.93) | 2 (0 - 5) | 2.46 (0.28 - 5.84) | -3.90 (-4.22 to -3.57) |
| Yemen | 335,518 (54,412 - 643,460) | 1213.19 (196.99 - 2311.26) | 22,574 (2,748 - 55,358) | 54.91 (6.58 - 133.14) | -9.82 (-10.28 to -9.37) |
| Zambia | 303,909 (44,260 - 579,406) | 2527.45 (372.97 - 4712.95) | 86,157 (11,935 - 165,986) | 453.08 (63.30 - 880.28) | -5.60 (-6.16 to -5.03) |
| Zimbabwe | 56,665 (7,551 - 108,697) | 478.48 (58.15 - 957.93) | 61,153 (7,324 - 129,206) | 404.45 (47.17 - 876.76) | 0.47 (-0.33 - 1.28) |

| Table S4. The YLDs and age-standardized YLDs Rate ofno access to handwashing facility in 1990 and 2021, with Temporal Trends from 1990 to 2021 in 204 countries or territories. | | | | | |
| --- | --- | --- | --- | --- | --- |
| **location** | **Num_1990** | **YLDs_1990** | **Num_2021** | **YLDs_2021** | **EAPC_CI** |
| Afghanistan | 1,995 (315 - 3,817) | 12.91 (2.03 - 24.79) | 3,403 (559 - 6,855) | 7.86 (1.31 - 15.83) | -1.29 (-1.76 to -0.82) |
| Albania | 26 (3 - 62) | 0.68 (0.07 - 1.62) | 3 (0 - 8) | 0.16 (0.02 - 0.42) | -4.66 (-5.22 to -4.10) |
| Algeria | 2,219 (267 - 4,975) | 6.31 (0.75 - 14.06) | 1,409 (178 - 3,377) | 3.16 (0.40 - 7.58) | -2.12 (-2.17 to -2.06) |
| American Samoa | 3 (0 - 7) | 5.67 (0.66 - 12.81) | 4 (0 - 8) | 7.09 (0.82 - 16.45) | 0.73 (0.51 - 0.96) |
| Andorra | 0 (0 - 1) | 1.04 (0.11 - 2.58) | 1 (0 - 1) | 0.93 (0.09 - 2.31) | -0.04 (-0.50 - 0.42) |
| Angola | 7,993 (1,403 - 14,752) | 58.55 (10.35 - 107.86) | 16,117 (2,624 - 31,543) | 48.16 (8.02 - 93.14) | -0.73 (-0.83 to -0.64) |
| Antigua and Barbuda | 4 (1 - 9) | 6.57 (0.82 - 14.35) | 4 (1 - 10) | 5.36 (0.64 - 12.63) | -0.70 (-0.74 to -0.66) |
| Argentina | 1,672 (186 - 3,969) | 4.94 (0.55 - 11.72) | 172 (18 - 410) | 0.41 (0.04 - 0.98) | -6.47 (-7.53 to -5.41) |
| Armenia | 168 (21 - 383) | 4.64 (0.58 - 10.50) | 12 (1 - 29) | 0.49 (0.05 - 1.17) | -7.54 (-8.38 to -6.69) |
| Australia | 72 (8 - 178) | 0.44 (0.05 - 1.10) | 20 (2 - 50) | 0.08 (0.01 - 0.19) | -6.21 (-7.25 to -5.17) |
| Austria | 155 (17 - 370) | 2.53 (0.28 - 6.01) | 53 (6 - 127) | 0.76 (0.08 - 1.82) | -4.30 (-5.31 to -3.28) |
| Azerbaijan | 251 (30 - 592) | 2.96 (0.36 - 6.98) | 80 (7 - 200) | 0.85 (0.07 - 2.15) | -4.66 (-5.23 to -4.09) |
| Bahamas | 9 (1 - 21) | 3.27 (0.42 - 7.46) | 8 (1 - 21) | 2.28 (0.24 - 5.56) | -0.79 (-1.20 to -0.37) |
| Bahrain | 12 (2 - 27) | 2.25 (0.30 - 5.27) | 17 (2 - 43) | 1.38 (0.15 - 3.53) | -2.06 (-2.43 to -1.69) |
| Bangladesh | 66,089 (11,262 - 126,006) | 46.48 (7.98 - 88.44) | 29,761 (4,151 - 60,087) | 17.77 (2.48 - 35.78) | -2.75 (-2.84 to -2.66) |
| Barbados | 9 (1 - 21) | 3.75 (0.45 - 8.53) | 13 (2 - 31) | 5.38 (0.61 - 13.09) | 1.20 (1.05 - 1.34) |
| Belarus | 149 (17 - 351) | 1.75 (0.20 - 4.12) | 41 (5 - 109) | 0.59 (0.06 - 1.55) | -3.82 (-3.97 to -3.67) |
| Belgium | 104 (12 - 237) | 1.24 (0.14 - 2.80) | 104 (11 - 250) | 0.76 (0.08 - 1.85) | -1.43 (-2.17 to -0.69) |
| Belize | 26 (4 - 55) | 11.37 (1.54 - 23.66) | 40 (5 - 84) | 8.82 (1.09 - 18.56) | -0.44 (-0.67 to -0.21) |
| Benin | 3,878 (684 - 7,100) | 63.28 (11.32 - 116.63) | 6,899 (1,248 - 12,807) | 50.28 (9.20 - 92.72) | -0.90 (-0.97 to -0.83) |
| Bermuda | 2 (0 - 5) | 4.15 (0.50 - 9.01) | 3 (0 - 6) | 5.46 (0.59 - 12.73) | 0.61 (0.46 - 0.77) |
| Bhutan | 179 (27 - 358) | 26.35 (3.99 - 52.52) | 44 (5 - 102) | 5.98 (0.66 - 13.89) | -5.58 (-5.88 to -5.28) |
| Bolivia (Plurinational State of) | 1,230 (155 - 2,642) | 14.32 (1.81 - 30.68) | 244 (28 - 575) | 2.05 (0.24 - 4.81) | -6.80 (-7.32 to -6.28) |
| Bosnia and Herzegovina | 23 (3 - 54) | 0.60 (0.07 - 1.43) | 3 (0 - 8) | 0.15 (0.02 - 0.38) | -4.67 (-4.97 to -4.38) |
| Botswana | 1,465 (244 - 2,709) | 100.16 (16.64 - 183.87) | 1,011 (172 - 2,015) | 40.61 (6.92 - 81.21) | -3.10 (-3.23 to -2.97) |
| Brazil | 18,522 (2,933 - 40,171) | 11.59 (1.83 - 25.01) | 4,523 (496 - 10,920) | 2.23 (0.25 - 5.45) | -5.90 (-6.16 to -5.64) |
| Brunei Darussalam | 1 (0 - 2) | 0.29 (0.03 - 0.76) | 1 (0 - 1) | 0.13 (0.01 - 0.31) | -2.61 (-2.98 to -2.24) |
| Bulgaria | 14 (2 - 37) | 0.24 (0.03 - 0.62) | 3 (0 - 7) | 0.07 (0.01 - 0.17) | -3.40 (-3.56 to -3.24) |
| Burkina Faso | 8,087 (1,461 - 14,850) | 66.40 (12.12 - 121.00) | 10,778 (1,913 - 20,157) | 46.99 (8.39 - 87.65) | -1.31 (-1.54 to -1.07) |
| Burundi | 4,663 (813 - 8,444) | 66.10 (11.66 - 119.41) | 8,141 (1,428 - 15,154) | 58.37 (10.44 - 108.93) | -0.32 (-0.53 to -0.11) |
| Cabo Verde | 3,311 (459 - 6,562) | 22.77 (3.17 - 45.49) | 1,335 (178 - 2,959) | 7.82 (1.04 - 17.28) | -3.97 (-4.32 to -3.61) |
| Cambodia | 8,461 (1,484 - 15,661) | 65.14 (11.52 - 120.77) | 14,309 (2,448 - 26,976) | 42.86 (7.32 - 79.70) | -1.44 (-1.64 to -1.25) |
| Cameroon | 86 (9 - 215) | 0.34 (0.03 - 0.86) | 19 (2 - 46) | 0.06 (0.01 - 0.15) | -5.16 (-6.04 to -4.26) |
| Canada | 301 (54 - 552) | 68.94 (12.45 - 125.32) | 166 (28 - 311) | 28.96 (4.80 - 54.44) | -3.76 (-4.28 to -3.24) |
| Central African Republic | 1,750 (300 - 3,204) | 48.16 (8.27 - 88.10) | 2,548 (439 - 4,761) | 45.03 (7.83 - 83.84) | -0.25 (-0.32 to -0.17) |
| Chad | 5,760 (1,012 - 10,590) | 75.22 (13.52 - 138.51) | 12,921 (2,254 - 23,736) | 68.24 (11.95 - 124.66) | -0.24 (-0.32 to -0.17) |
| Chile | 216 (24 - 526) | 1.64 (0.18 - 3.96) | 35 (4 - 87) | 0.21 (0.02 - 0.52) | -7.23 (-8.07 to -6.38) |
| China | 63,653 (8,313 - 134,816) | 5.56 (0.73 - 11.76) | 4,443 (503 - 9,952) | 0.36 (0.04 - 0.80) | -9.47 (-9.94 to -8.99) |
| Colombia | 2,152 (327 - 4,951) | 6.02 (0.91 - 13.70) | 770 (89 - 1,878) | 1.69 (0.19 - 4.15) | -4.63 (-5.14 to -4.12) |
| Comoros | 382 (66 - 698) | 65.83 (11.50 - 120.91) | 392 (66 - 739) | 51.60 (8.75 - 97.03) | -0.97 (-1.18 to -0.75) |
| Congo | 1,427 (209 - 2,730) | 47.75 (7.03 - 91.18) | 2,094 (314 - 4,081) | 38.70 (5.81 - 75.97) | -0.48 (-0.75 to -0.21) |
| Cook Islands | 1 (0 - 3) | 6.01 (0.73 - 14.31) | 1 (0 - 3) | 8.19 (0.86 - 18.71) | 1.10 (0.82 - 1.37) |
| Costa Rica | 114 (13 - 257) | 3.89 (0.46 - 8.84) | 129 (14 - 321) | 2.85 (0.30 - 7.02) | -0.08 (-0.42 - 0.27) |
| Côte d'Ivoire | 4 (0 - 9) | 0.10 (0.01 - 0.26) | 2 (0 - 5) | 0.06 (0.01 - 0.15) | -1.66 (-1.82 to -1.50) |
| Croatia | 487 (62 - 1,102) | 4.77 (0.61 - 10.71) | 462 (51 - 1,068) | 4.68 (0.50 - 10.89) | -0.33 (-0.59 to -0.08) |
| Cuba | 12 (1 - 30) | 1.73 (0.17 - 4.13) | 15 (2 - 39) | 1.29 (0.16 - 3.27) | -0.32 (-0.70 - 0.06) |
| Cyprus | 7 (1 - 17) | 0.09 (0.01 - 0.22) | 12 (1 - 28) | 0.08 (0.01 - 0.21) | 0.32 (0.10 - 0.54) |
| Czechia | 8,478 (1,486 - 15,311) | 55.96 (9.98 - 102.41) | 12,502 (2,286 - 23,419) | 43.44 (7.89 - 81.14) | -0.86 (-0.99 to -0.73) |
| Democratic People's Republic of Korea | 46 (4 - 117) | 1.09 (0.09 - 2.76) | 68 (6 - 176) | 1.17 (0.11 - 3.02) | 0.86 (0.34 - 1.38) |
| Democratic Republic of the Congo | 333 (56 - 610) | 67.27 (11.34 - 123.92) | 616 (102 - 1,153) | 48.21 (8.05 - 89.71) | -1.34 (-1.58 to -1.10) |
| Denmark | 5 (1 - 11) | 6.36 (0.83 - 13.56) | 3 (0 - 6) | 4.48 (0.55 - 10.26) | -1.19 (-1.26 to -1.12) |
| Djibouti | 1,406 (218 - 2,891) | 15.90 (2.46 - 32.72) | 748 (113 - 1,549) | 6.87 (1.03 - 14.27) | -2.77 (-3.18 to -2.36) |
| Dominica | 24,732 (4,278 - 44,981) | 48.19 (8.39 - 87.47) | 38,870 (6,824 - 72,386) | 41.26 (7.38 - 75.80) | -0.51 (-0.66 to -0.37) |
| Dominican Republic | 2,917 (364 - 6,459) | 25.32 (3.13 - 55.86) | 371 (47 - 863) | 2.07 (0.26 - 4.81) | -9.23 (-9.95 to -8.51) |
| Ecuador | 7,756 (969 - 16,564) | 9.91 (1.24 - 21.38) | 3,128 (427 - 7,433) | 2.64 (0.36 - 6.28) | -3.99 (-4.11 to -3.87) |
| Egypt | 914 (121 - 1,958) | 14.94 (2.00 - 31.67) | 161 (17 - 384) | 2.50 (0.26 - 5.99) | -6.62 (-7.19 to -6.04) |
| El Salvador | 307 (55 - 564) | 52.93 (9.44 - 97.59) | 519 (76 - 1,024) | 34.34 (5.08 - 68.27) | -1.73 (-2.11 to -1.34) |
| Equatorial Guinea | 3,514 (601 - 6,326) | 83.00 (14.27 - 149.56) | 5,692 (983 - 10,547) | 84.99 (14.87 - 157.66) | -0.11 (-0.19 to -0.02) |
| Eritrea | 14 (2 - 37) | 1.12 (0.12 - 2.91) | 5 (1 - 13) | 0.53 (0.06 - 1.32) | -1.99 (-2.25 to -1.74) |
| Estonia | 879 (143 - 1,633) | 93.59 (15.27 - 174.59) | 701 (121 - 1,358) | 57.53 (9.87 - 110.87) | -1.60 (-1.66 to -1.55) |
| Eswatini | 48,266 (8,493 - 88,533) | 79.38 (14.23 - 145.10) | 65,089 (11,746 - 121,211) | 59.12 (10.88 - 110.43) | -1.15 (-1.22 to -1.08) |
| Ethiopia | 16 (2 - 33) | 12.42 (1.68 - 26.05) | 8 (1 - 19) | 7.96 (1.00 - 17.49) | -2.02 (-2.30 to -1.73) |
| Fiji | 116 (15 - 240) | 14.36 (1.85 - 29.41) | 84 (11 - 184) | 9.03 (1.22 - 19.85) | -1.31 (-1.60 to -1.01) |
| Finland | 53 (5 - 136) | 1.28 (0.12 - 3.21) | 24 (3 - 62) | 0.58 (0.07 - 1.49) | -2.54 (-3.34 to -1.74) |
| France | 736 (86 - 1,693) | 1.45 (0.17 - 3.38) | 1,083 (103 - 2,597) | 1.94 (0.18 - 4.73) | 1.41 (0.97 - 1.86) |
| Gabon | 548 (88 - 1,036) | 47.76 (7.69 - 89.46) | 535 (81 - 1,104) | 29.25 (4.44 - 59.94) | -1.63 (-1.73 to -1.53) |
| Gambia | 110 (11 - 273) | 2.23 (0.23 - 5.56) | 16 (2 - 40) | 0.53 (0.05 - 1.32) | -3.99 (-5.04 to -2.94) |
| Georgia | 816 (80 - 2,019) | 1.30 (0.13 - 3.22) | 517 (60 - 1,228) | 0.56 (0.06 - 1.33) | -2.04 (-3.07 to -0.99) |
| Germany | 11,418 (2,010 - 21,291) | 60.39 (10.72 - 111.53) | 13,392 (2,310 - 25,450) | 38.01 (6.58 - 71.95) | -1.92 (-2.07 to -1.76) |
| Ghana | 137 (12 - 365) | 1.63 (0.14 - 4.29) | 69 (8 - 178) | 0.95 (0.11 - 2.44) | -1.01 (-1.57 to -0.45) |
| Greece | 0 (0 - 0) | 0.13 (0.01 - 0.34) | 0 (0 - 0) | 0.04 (0.00 - 0.11) | -4.13 (-4.62 to -3.63) |
| Greenland | 10 (2 - 22) | 10.21 (1.49 - 22.11) | 10 (1 - 22) | 10.41 (1.29 - 23.34) | 0.00 (-0.23 - 0.24) |
| Grenada | 7 (1 - 17) | 4.77 (0.56 - 11.83) | 9 (1 - 22) | 6.02 (0.70 - 14.70) | 0.51 (0.18 - 0.84) |
| Guam | 3,721 (560 - 7,603) | 37.38 (5.62 - 76.46) | 1,031 (131 - 2,262) | 6.40 (0.81 - 14.08) | -6.18 (-6.43 to -5.92) |
| Guatemala | 4,815 (838 - 8,918) | 64.62 (11.34 - 118.19) | 6,723 (1,158 - 12,595) | 48.82 (8.47 - 91.74) | -1.25 (-1.40 to -1.09) |
| Guinea | 742 (127 - 1,383) | 59.25 (10.08 - 109.63) | 1,031 (179 - 1,890) | 49.72 (8.59 - 89.86) | -0.84 (-1.03 to -0.64) |
| Guinea-Bissau | 83 (12 - 171) | 9.55 (1.40 - 19.62) | 40 (5 - 88) | 5.24 (0.71 - 11.51) | -2.35 (-2.61 to -2.09) |
| Guyana | 1,251 (218 - 2,315) | 14.00 (2.45 - 25.70) | 1,168 (187 - 2,236) | 8.48 (1.37 - 16.18) | 0.03 (-1.22 - 1.31) |
| Haiti | 680 (89 - 1,468) | 11.89 (1.58 - 25.49) | 329 (43 - 737) | 3.26 (0.43 - 7.35) | -4.64 (-4.91 to -4.38) |
| Honduras | 29 (3 - 75) | 0.40 (0.05 - 1.05) | 13 (1 - 35) | 0.13 (0.01 - 0.34) | -3.67 (-4.16 to -3.19) |
| Hungary | 3 (0 - 8) | 1.24 (0.13 - 3.26) | 3 (0 - 8) | 1.10 (0.10 - 2.70) | 0.06 (-0.43 - 0.55) |
| Iceland | 489,431 (75,905 - 939,752) | 53.34 (8.27 - 102.94) | 459,454 (63,042 - 950,796) | 32.71 (4.49 - 67.51) | -1.71 (-1.79 to -1.63) |
| India | 49,130 (7,146 - 102,828) | 25.36 (3.66 - 52.96) | 17,783 (1,888 - 40,152) | 7.04 (0.75 - 15.93) | -3.89 (-4.02 to -3.75) |
| Indonesia | 6,840 (782 - 15,858) | 8.56 (0.99 - 19.77) | 1,415 (148 - 3,345) | 1.76 (0.18 - 4.18) | -5.07 (-5.42 to -4.71) |
| Iran (Islamic Republic of) | 1,707 (188 - 4,161) | 6.25 (0.69 - 15.20) | 663 (65 - 1,654) | 1.48 (0.15 - 3.67) | -4.46 (-4.79 to -4.13) |
| Iraq | 49 (5 - 128) | 1.41 (0.15 - 3.71) | 46 (5 - 110) | 1.05 (0.11 - 2.54) | 0.95 (-0.03 - 1.94) |
| Ireland | 69 (8 - 174) | 1.34 (0.15 - 3.40) | 94 (11 - 235) | 0.91 (0.10 - 2.28) | -0.83 (-1.62 to -0.03) |
| Israel | 409 (41 - 998) | 0.93 (0.09 - 2.30) | 178 (22 - 429) | 0.32 (0.04 - 0.79) | -3.17 (-3.38 to -2.95) |
| Italy | 269 (37 - 590) | 10.37 (1.44 - 22.72) | 109 (13 - 247) | 4.09 (0.47 - 9.29) | -2.59 (-2.95 to -2.22) |
| Jamaica | 996 (110 - 2,532) | 0.96 (0.11 - 2.43) | 880 (84 - 2,200) | 0.94 (0.09 - 2.38) | 0.49 (0.12 - 0.86) |
| Japan | 186 (22 - 432) | 3.52 (0.42 - 8.20) | 198 (22 - 490) | 1.59 (0.18 - 3.90) | -1.82 (-2.45 to -1.18) |
| Jordan | 184 (20 - 451) | 1.02 (0.11 - 2.51) | 35 (4 - 87) | 0.19 (0.02 - 0.47) | -5.52 (-5.73 to -5.32) |
| Kazakhstan | 24,310 (4,127 - 45,406) | 96.25 (16.52 - 177.21) | 39,979 (6,864 - 73,042) | 79.13 (13.44 - 145.08) | -0.57 (-0.63 to -0.51) |
| Kenya | 23 (3 - 48) | 29.15 (3.92 - 59.17) | 24 (3 - 48) | 19.90 (2.89 - 40.72) | -1.48 (-1.58 to -1.39) |
| Kiribati | 40 (5 - 96) | 2.11 (0.25 - 5.12) | 44 (5 - 113) | 1.14 (0.13 - 2.88) | -1.11 (-1.33 to -0.89) |
| Kuwait | 146 (16 - 354) | 2.50 (0.27 - 6.07) | 43 (5 - 108) | 0.58 (0.07 - 1.45) | -3.84 (-4.19 to -3.49) |
| Kyrgyzstan | 1,577 (224 - 3,049) | 26.45 (3.74 - 51.51) | 1,046 (153 - 2,235) | 13.74 (2.02 - 29.39) | -2.03 (-2.13 to -1.93) |
| Lao People's Democratic Republic | 26 (3 - 66) | 1.22 (0.12 - 3.10) | 5 (1 - 13) | 0.38 (0.04 - 0.96) | -3.32 (-3.56 to -3.08) |
| Latvia | 135 (17 - 303) | 3.90 (0.50 - 8.77) | 138 (15 - 356) | 2.69 (0.30 - 6.98) | -1.58 (-1.84 to -1.33) |
| Lebanon | 1,856 (336 - 3,394) | 107.98 (19.62 - 196.09) | 1,494 (263 - 2,745) | 76.16 (13.46 - 139.82) | -1.32 (-1.46 to -1.17) |
| Lesotho | 2,013 (347 - 3,659) | 62.97 (11.01 - 114.92) | 4,387 (767 - 8,197) | 76.05 (13.48 - 142.97) | 0.59 (0.33 - 0.85) |
| Liberia | 363 (44 - 814) | 6.39 (0.78 - 14.30) | 176 (21 - 435) | 2.72 (0.32 - 6.70) | -2.67 (-2.94 to -2.39) |
| Libya | 44 (5 - 107) | 1.43 (0.17 - 3.46) | 12 (1 - 32) | 0.56 (0.06 - 1.52) | -3.02 (-3.21 to -2.83) |
| Lithuania | 3 (0 - 9) | 1.10 (0.11 - 2.80) | 4 (0 - 9) | 0.61 (0.06 - 1.50) | -1.80 (-2.56 to -1.04) |
| Luxembourg | 15,351 (2,736 - 28,058) | 98.77 (17.61 - 180.21) | 28,012 (5,089 - 51,419) | 87.94 (16.03 - 163.08) | -0.26 (-0.38 to -0.14) |
| Madagascar | 8,771 (1,595 - 16,177) | 70.07 (12.69 - 129.22) | 11,655 (2,161 - 21,429) | 56.80 (10.62 - 105.22) | -0.79 (-0.98 to -0.59) |
| Malawi | 467 (47 - 1,158) | 2.34 (0.24 - 5.86) | 807 (80 - 2,148) | 2.67 (0.27 - 7.12) | 0.76 (0.33 - 1.20) |
| Malaysia | 27 (3 - 62) | 8.09 (1.01 - 18.49) | 13 (1 - 31) | 2.89 (0.29 - 7.03) | -3.40 (-3.65 to -3.15) |
| Maldives | 8,475 (1,454 - 15,472) | 79.48 (13.73 - 143.14) | 14,995 (2,622 - 28,256) | 64.96 (11.58 - 121.70) | -0.88 (-1.05 to -0.71) |
| Mali | 5 (1 - 13) | 1.65 (0.15 - 4.02) | 4 (0 - 10) | 1.18 (0.14 - 2.99) | -0.64 (-1.07 to -0.21) |
| Malta | 8 (1 - 17) | 15.06 (2.09 - 31.59) | 5 (1 - 11) | 8.73 (1.37 - 19.10) | -2.06 (-2.19 to -1.93) |
| Marshall Islands | 1,570 (275 - 2,910) | 64.65 (11.45 - 118.42) | 2,938 (478 - 5,643) | 65.56 (10.76 - 125.81) | -0.29 (-0.51 to -0.06) |
| Mauritania | 29 (3 - 71) | 2.59 (0.29 - 6.43) | 9 (1 - 20) | 0.87 (0.09 - 2.04) | -2.69 (-3.17 to -2.21) |
| Mauritius | 9,886 (1,246 - 22,535) | 10.32 (1.32 - 23.59) | 1,328 (154 - 3,164) | 1.08 (0.13 - 2.56) | -7.37 (-7.75 to -6.99) |
| Mexico | 177 (23 - 406) | 4.19 (0.54 - 9.61) | 21 (3 - 51) | 0.90 (0.10 - 2.17) | -4.65 (-5.32 to -3.96) |
| Micronesia (Federated States of) | 0 (0 - 0) | 0.95 (0.09 - 2.39) | 0 (0 - 1) | 0.87 (0.08 - 2.12) | 0.11 (-0.34 - 0.57) |
| Monaco | 160 (22 - 344) | 5.02 (0.68 - 10.81) | 18 (2 - 40) | 0.51 (0.06 - 1.11) | -6.78 (-8.01 to -5.54) |
| Mongolia | 1 (0 - 4) | 0.27 (0.03 - 0.68) | 0 (0 - 1) | 0.11 (0.01 - 0.27) | -2.38 (-2.95 to -1.80) |
| Montenegro | 4,887 (729 - 10,159) | 14.56 (2.16 - 30.27) | 853 (98 - 1,951) | 2.40 (0.28 - 5.49) | -5.79 (-6.25 to -5.34) |
| Morocco | 9,091 (1,579 - 16,440) | 51.44 (8.99 - 93.67) | 11,614 (2,053 - 21,418) | 36.13 (6.44 - 67.48) | -1.45 (-1.55 to -1.35) |
| Mozambique | 8,036 (1,134 - 17,201) | 17.02 (2.40 - 36.24) | 3,569 (432 - 7,600) | 6.49 (0.78 - 13.84) | -3.54 (-3.78 to -3.31) |
| Myanmar | 1,189 (204 - 2,199) | 76.37 (13.34 - 141.95) | 1,317 (203 - 2,731) | 52.41 (8.12 - 108.62) | -1.19 (-1.28 to -1.10) |
| Namibia | 1 (0 - 2) | 7.78 (1.04 - 16.87) | 1 (0 - 2) | 7.78 (1.05 - 17.63) | 0.01 (-0.03 - 0.05) |
| Nauru | 8,947 (1,316 - 17,369) | 35.98 (5.34 - 70.14) | 3,643 (493 - 7,505) | 11.78 (1.60 - 24.21) | -3.05 (-3.39 to -2.70) |
| Nepal | 179 (21 - 437) | 1.46 (0.17 - 3.60) | 311 (37 - 767) | 2.23 (0.27 - 5.56) | 1.85 (1.17 - 2.54) |
| Netherlands | 28 (3 - 68) | 0.86 (0.08 - 2.08) | 14 (1 - 36) | 0.26 (0.03 - 0.66) | -3.43 (-3.82 to -3.04) |
| New Zealand | 717 (96 - 1,553) | 13.71 (1.86 - 29.81) | 178 (22 - 422) | 2.65 (0.33 - 6.27) | -6.00 (-6.39 to -5.61) |
| Nicaragua | 7,974 (1,406 - 14,975) | 73.17 (13.04 - 136.07) | 14,762 (2,620 - 27,120) | 56.13 (10.05 - 103.68) | -0.91 (-1.15 to -0.66) |
| Niger | 94,843 (16,559 - 173,985) | 93.17 (16.50 - 170.98) | 106,883 (17,547 - 202,641) | 43.19 (6.99 - 81.34) | -2.73 (-2.85 to -2.60) |
| Nigeria | 0 (0 - 0) | 8.22 (0.94 - 18.17) | 0 (0 - 0) | 5.89 (0.72 - 13.40) | -0.71 (-0.93 to -0.48) |
| Niue | 368 (39 - 872) | 1.67 (0.18 - 3.97) | 1,046 (121 - 2,545) | 4.44 (0.51 - 10.88) | 4.57 (4.04 - 5.11) |
| North Macedonia | 16 (1 - 38) | 0.90 (0.09 - 2.22) | 2 (0 - 4) | 0.13 (0.01 - 0.33) | -5.81 (-6.15 to -5.48) |
| Northern Mariana Islands | 2 (0 - 4) | 4.27 (0.49 - 10.22) | 3 (0 - 8) | 6.72 (0.74 - 16.66) | 1.69 (1.37 - 2.01) |
| Norway | 38 (4 - 101) | 0.98 (0.11 - 2.63) | 49 (5 - 117) | 0.68 (0.07 - 1.64) | -1.50 (-1.72 to -1.28) |
| Oman | 156 (21 - 341) | 6.28 (0.86 - 13.60) | 62 (7 - 155) | 1.60 (0.18 - 4.01) | -4.54 (-5.05 to -4.02) |
| Pakistan | 65,199 (9,675 - 129,424) | 48.84 (7.39 - 98.25) | 45,449 (6,195 - 98,577) | 18.34 (2.54 - 40.08) | -3.04 (-3.15 to -2.93) |
| Palau | 1 (0 - 2) | 6.40 (0.83 - 14.74) | 1 (0 - 2) | 5.09 (0.55 - 12.85) | -0.93 (-1.13 to -0.73) |
| Palestine | 196 (26 - 418) | 6.49 (0.85 - 13.84) | 101 (13 - 237) | 1.77 (0.22 - 4.11) | -2.94 (-3.67 to -2.21) |
| Panama | 279 (34 - 648) | 10.61 (1.28 - 24.59) | 153 (19 - 376) | 3.63 (0.45 - 8.92) | -3.57 (-3.81 to -3.32) |
| Papua New Guinea | 1,782 (297 - 3,390) | 37.37 (6.19 - 71.21) | 3,092 (495 - 6,057) | 27.77 (4.45 - 54.23) | -1.28 (-1.37 to -1.19) |
| Paraguay | 1,740 (268 - 3,484) | 36.58 (5.70 - 72.27) | 115 (13 - 269) | 1.65 (0.19 - 3.84) | -10.89 (-11.39 to -10.39) |
| Peru | 5,298 (643 - 11,403) | 21.20 (2.57 - 45.86) | 1,210 (136 - 2,863) | 3.41 (0.38 - 8.13) | -6.32 (-6.64 to -6.00) |
| Philippines | 13,544 (1,789 - 29,621) | 17.15 (2.26 - 37.53) | 4,918 (544 - 11,839) | 4.24 (0.47 - 10.18) | -4.69 (-4.88 to -4.51) |
| Poland | 61 (7 - 158) | 0.20 (0.02 - 0.52) | 6 (1 - 14) | 0.02 (0.00 - 0.05) | -7.24 (-8.03 to -6.45) |
| Portugal | 118 (11 - 274) | 1.51 (0.14 - 3.44) | 60 (8 - 151) | 0.64 (0.08 - 1.70) | -2.06 (-2.31 to -1.80) |
| Puerto Rico | 166 (20 - 369) | 4.65 (0.57 - 10.40) | 109 (12 - 249) | 3.68 (0.40 - 8.36) | -0.43 (-0.85 - -0.00) |
| Qatar | 7 (1 - 17) | 1.54 (0.16 - 3.77) | 19 (2 - 44) | 0.93 (0.09 - 2.14) | -1.84 (-2.03 to -1.66) |
| Republic of Korea | 66 (8 - 158) | 0.38 (0.04 - 0.91) | 10 (1 - 24) | 0.08 (0.01 - 0.18) | -5.16 (-5.23 to -5.08) |
| Republic of Moldova | 2,470 (290 - 6,106) | 1.98 (0.23 - 4.85) | 522 (65 - 1,311) | 0.45 (0.06 - 1.13) | -4.28 (-4.73 to -3.83) |
| Romania | 5,771 (1,019 - 10,453) | 59.28 (10.50 - 107.65) | 6,659 (1,226 - 12,267) | 48.71 (9.08 - 89.54) | -0.74 (-0.98 to -0.50) |
| Russian Federation | 3 (0 - 7) | 6.86 (0.86 - 15.24) | 3 (0 - 6) | 5.27 (0.65 - 12.25) | -0.97 (-1.05 to -0.89) |
| Rwanda | 12 (2 - 26) | 8.11 (1.12 - 17.05) | 13 (1 - 31) | 8.60 (0.95 - 20.41) | 0.17 (0.12 - 0.22) |
| Saint Kitts and Nevis | 12 (2 - 27) | 10.08 (1.33 - 21.66) | 7 (1 - 15) | 6.54 (0.88 - 14.54) | -1.31 (-1.46 to -1.16) |
| Saint Lucia | 11 (1 - 24) | 5.10 (0.65 - 11.78) | 10 (1 - 25) | 4.11 (0.45 - 10.21) | -0.78 (-0.86 to -0.70) |
| Saint Vincent and the Grenadines | 0 (0 - 0) | 1.02 (0.11 - 2.45) | 0 (0 - 1) | 0.92 (0.09 - 2.40) | 0.08 (-0.33 - 0.48) |
| Samoa | 1,166 (136 - 2,628) | 5.79 (0.68 - 13.10) | 584 (61 - 1,500) | 1.80 (0.19 - 4.64) | -3.14 (-3.56 to -2.72) |
| San Marino | 7,233 (1,239 - 13,560) | 75.66 (12.91 - 140.47) | 7,997 (1,255 - 15,493) | 48.72 (7.73 - 93.21) | -1.57 (-1.73 to -1.41) |
| Sao Tome and Principe | 25 (3 - 58) | 0.32 (0.03 - 0.75) | 10 (1 - 23) | 0.14 (0.02 - 0.34) | -1.83 (-2.52 to -1.13) |
| Saudi Arabia | 2 (0 - 4) | 2.29 (0.21 - 5.70) | 2 (0 - 4) | 1.64 (0.18 - 4.08) | -0.28 (-0.70 - 0.14) |
| Senegal | 3,806 (655 - 7,014) | 74.84 (13.05 - 136.86) | 5,720 (1,000 - 10,627) | 62.97 (10.96 - 115.56) | -0.90 (-0.99 to -0.81) |
| Serbia | 2 (0 - 4) | 0.07 (0.01 - 0.18) | 1 (0 - 2) | 0.02 (0.00 - 0.05) | -3.32 (-3.71 to -2.92) |
| Seychelles | 4 (0 - 9) | 0.08 (0.01 - 0.20) | 2 (0 - 4) | 0.04 (0.00 - 0.10) | -1.88 (-2.02 to -1.73) |
| Sierra Leone | 2 (0 - 5) | 0.15 (0.02 - 0.38) | 1 (0 - 4) | 0.10 (0.01 - 0.27) | -0.76 (-0.96 to -0.56) |
| Singapore | 90 (15 - 179) | 23.39 (3.99 - 45.76) | 110 (16 - 234) | 15.79 (2.29 - 33.15) | -1.33 (-1.35 to -1.31) |
| Slovakia | 5,743 (989 - 10,504) | 55.95 (9.68 - 102.81) | 9,078 (1,599 - 16,729) | 40.87 (7.25 - 75.94) | -1.20 (-1.31 to -1.08) |
| Slovenia | 34,524 (5,392 - 66,411) | 83.93 (13.21 - 160.90) | 22,094 (3,250 - 45,930) | 38.25 (5.61 - 79.64) | -2.54 (-2.65 to -2.43) |
| Solomon Islands | 152 (17 - 384) | 0.35 (0.04 - 0.88) | 53 (6 - 143) | 0.14 (0.01 - 0.36) | -2.69 (-3.06 to -2.32) |
| Somalia | 5,792 (983 - 10,574) | 81.48 (13.88 - 148.75) | 9,803 (1,729 - 17,748) | 98.40 (17.65 - 178.07) | 0.62 (0.59 - 0.66) |
| South Africa | 497 (52 - 1,276) | 1.56 (0.16 - 3.97) | 403 (50 - 952) | 1.04 (0.13 - 2.45) | -0.98 (-1.27 to -0.69) |
| South Sudan | 1,783 (226 - 4,130) | 10.18 (1.29 - 23.73) | 673 (78 - 1,678) | 3.19 (0.37 - 8.01) | -3.90 (-4.09 to -3.70) |
| Spain | 6,019 (949 - 12,405) | 19.46 (3.05 - 40.22) | 4,894 (676 - 11,304) | 9.56 (1.33 - 22.00) | -2.30 (-2.98 to -1.62) |
| Sri Lanka | 39 (6 - 85) | 9.34 (1.34 - 20.36) | 29 (3 - 65) | 5.15 (0.62 - 11.46) | -1.98 (-2.03 to -1.93) |
| Sudan | 65 (6 - 158) | 0.85 (0.08 - 2.06) | 109 (12 - 275) | 0.79 (0.08 - 1.98) | 0.51 (0.25 - 0.78) |
| Suriname | 58 (6 - 155) | 1.06 (0.10 - 2.85) | 77 (7 - 186) | 1.01 (0.09 - 2.51) | 0.25 (-0.21 - 0.71) |
| Sweden | 1,272 (167 - 2,787) | 6.50 (0.86 - 14.25) | 359 (44 - 827) | 2.50 (0.30 - 5.80) | -2.99 (-3.17 to -2.80) |
| Switzerland | 70 (12 - 129) | 44.33 (7.96 - 81.53) | 46 (7 - 87) | 19.69 (3.19 - 37.45) | -2.72 (-3.22 to -2.22) |
| Syrian Arab Republic | 251 (25 - 597) | 1.46 (0.15 - 3.45) | 259 (28 - 645) | 1.21 (0.13 - 3.01) | 0.75 (0.05 - 1.46) |
| Taiwan (Province of China) | 1,073 (148 - 2,305) | 13.41 (1.85 - 28.65) | 540 (69 - 1,244) | 4.76 (0.62 - 11.00) | -3.84 (-4.24 to -3.43) |
| Tajikistan | 21,918 (3,780 - 40,670) | 69.95 (12.24 - 129.67) | 23,190 (3,695 - 44,043) | 38.60 (6.24 - 74.48) | -2.18 (-2.44 to -1.91) |
| Thailand | 7,986 (1,093 - 17,900) | 14.32 (1.96 - 32.14) | 4,351 (608 - 10,266) | 6.20 (0.84 - 14.86) | -2.55 (-2.77 to -2.33) |
| Timor-Leste | 616 (106 - 1,120) | 51.65 (8.97 - 93.79) | 857 (149 - 1,617) | 34.97 (6.13 - 65.22) | -1.60 (-1.87 to -1.34) |
| Togo | 239 (31 - 485) | 21.90 (2.85 - 44.44) | 137 (17 - 322) | 8.81 (1.09 - 20.62) | -3.08 (-3.16 to -3.00) |
| Tokelau | 3,567 (576 - 6,750) | 77.48 (12.76 - 144.89) | 5,027 (890 - 9,231) | 58.19 (10.31 - 106.32) | -1.06 (-1.14 to -0.98) |
| Tonga | 0 (0 - 0) | 12.43 (1.92 - 25.80) | 0 (0 - 0) | 10.39 (1.24 - 23.55) | -0.76 (-1.02 to -0.49) |
| Trinidad and Tobago | 10 (1 - 22) | 8.89 (1.11 - 18.83) | 12 (1 - 26) | 10.01 (1.22 - 22.25) | 0.80 (0.54 - 1.07) |
| Tunisia | 53 (6 - 120) | 4.09 (0.47 - 9.01) | 37 (5 - 88) | 3.03 (0.37 - 7.19) | -0.93 (-1.03 to -0.84) |
| Turkey | 577 (68 - 1,274) | 5.70 (0.67 - 12.55) | 286 (33 - 692) | 2.64 (0.30 - 6.37) | -1.90 (-2.22 to -1.58) |
| Turkmenistan | 5,207 (652 - 11,671) | 7.64 (0.96 - 17.01) | 1,705 (204 - 4,080) | 2.23 (0.27 - 5.38) | -3.52 (-3.90 to -3.13) |
| Tuvalu | 82 (8 - 199) | 1.57 (0.16 - 3.79) | 9 (1 - 24) | 0.17 (0.02 - 0.45) | -8.03 (-8.62 to -7.44) |
| Uganda | 1 (0 - 3) | 11.96 (1.71 - 24.43) | 1 (0 - 3) | 9.47 (1.28 - 20.79) | -0.68 (-0.95 to -0.40) |
| Ukraine | 15,579 (2,751 - 27,868) | 69.55 (12.40 - 125.33) | 25,960 (4,467 - 48,051) | 58.10 (10.09 - 107.33) | -0.52 (-0.64 to -0.39) |
| United Arab Emirates | 597 (67 - 1,474) | 1.24 (0.13 - 3.03) | 318 (34 - 781) | 0.52 (0.06 - 1.31) | -2.18 (-3.17 to -1.17) |
| United Kingdom | 629 (72 - 1,528) | 1.57 (0.18 - 3.82) | 239 (26 - 593) | 0.75 (0.08 - 1.85) | -2.89 (-3.10 to -2.68) |
| United Republic of Tanzania | 42 (5 - 101) | 2.31 (0.26 - 5.50) | 81 (11 - 211) | 1.10 (0.15 - 2.85) | -2.04 (-2.25 to -1.82) |
| United States of America | 43 (5 - 104) | 1.42 (0.16 - 3.43) | 10 (1 - 24) | 0.30 (0.03 - 0.73) | -5.19 (-6.35 to -4.01) |
| United States Virgin Islands | 333 (40 - 847) | 0.15 (0.02 - 0.37) | 42 (4 - 107) | 0.01 (0.00 - 0.03) | -8.60 (-9.96 to -7.21) |
| Uruguay | 1,110 (136 - 2,490) | 3.67 (0.45 - 8.26) | 98 (10 - 233) | 0.28 (0.03 - 0.67) | -7.93 (-8.14 to -7.73) |
| Uzbekistan | 41 (6 - 80) | 23.16 (3.45 - 45.23) | 53 (7 - 114) | 16.36 (2.12 - 35.33) | -1.31 (-1.45 to -1.18) |
| Vanuatu | 816 (105 - 1,829) | 4.12 (0.54 - 9.26) | 1,088 (134 - 2,802) | 4.29 (0.53 - 11.03) | -0.17 (-0.38 - 0.04) |
| Venezuela (Bolivarian Republic of) | 9,785 (1,348 - 21,332) | 11.73 (1.63 - 25.73) | 4,774 (545 - 11,702) | 5.06 (0.58 - 12.44) | -2.11 (-2.33 to -1.88) |
| Viet Nam | 4 (0 - 10) | 3.90 (0.44 - 8.79) | 2 (0 - 4) | 2.87 (0.36 - 6.78) | -1.00 (-1.33 to -0.67) |
| Yemen | 6,987 (1,112 - 13,476) | 29.34 (4.67 - 56.78) | 3,906 (545 - 8,013) | 9.76 (1.37 - 20.01) | -2.11 (-2.90 to -1.31) |
| Zambia | 6,180 (1,096 - 11,369) | 60.21 (10.64 - 111.35) | 9,413 (1,574 - 17,320) | 46.89 (7.93 - 87.42) | -1.03 (-1.35 to -0.71) |
| Zimbabwe | 4,550 (698 - 8,994) | 39.39 (6.04 - 78.41) | 4,430 (704 - 8,961) | 27.93 (4.42 - 56.02) | -1.16 (-1.34 to -0.98) |
